# Continuous Influx of Genetic Material from Host to Virus Populations

**DOI:** 10.1371/journal.pgen.1005838

**Published:** 2016-02-01

**Authors:** Clément Gilbert, Jean Peccoud, Aurélien Chateigner, Bouziane Moumen, Richard Cordaux, Elisabeth A. Herniou

**Affiliations:** 1 UMR CNRS 7267 Ecologie et Biologie des Interactions, Equipe Ecologie Evolution Symbiose, Université de Poitiers, Poitiers, France; 2 Institut de Recherche sur la Biologie de l’Insecte, UMR CNRS 7261, UFR des Sciences et Techniques, Université François-Rabelais, Tours, France; Fred Hutchinson Cancer Research Center, UNITED STATES

## Abstract

Many genes of large double-stranded DNA viruses have a cellular origin, suggesting that host-to-virus horizontal transfer (HT) of DNA is recurrent. Yet, the frequency of these transfers has never been assessed in viral populations. Here we used ultra-deep DNA sequencing of 21 baculovirus populations extracted from two moth species to show that a large diversity of moth DNA sequences (n = 86) can integrate into viral genomes during the course of a viral infection. The majority of the 86 different moth DNA sequences are transposable elements (TEs, n = 69) belonging to 10 superfamilies of DNA transposons and three superfamilies of retrotransposons. The remaining 17 sequences are moth sequences of unknown nature. In addition to *bona fide* DNA transposition, we uncover microhomology-mediated recombination as a mechanism explaining integration of moth sequences into viral genomes. Many sequences integrated multiple times at multiple positions along the viral genome. We detected a total of 27,504 insertions of moth sequences in the 21 viral populations and we calculate that on average, 4.8% of viruses harbor at least one moth sequence in these populations. Despite this substantial proportion, no insertion of moth DNA was maintained in any viral population after 10 successive infection cycles. Hence, there is a constant turnover of host DNA inserted into viral genomes each time the virus infects a moth. Finally, we found that at least 21 of the moth TEs integrated into viral genomes underwent repeated horizontal transfers between various insect species, including some lepidopterans susceptible to baculoviruses. Our results identify host DNA influx as a potent source of genetic diversity in viral populations. They also support a role for baculoviruses as vectors of DNA HT between insects, and call for an evaluation of possible gene or TE spread when using viruses as biopesticides or gene delivery vectors.

## Author Summary

While gene exchange is known to occur between viruses and their hosts, this phenomenon has never been studied at the level of the viral population. Here we report that each time a virus from the *Baculoviridae* family infects a moth, a large number (dozens to hundreds) and high diversity of moth DNA sequences (86 different sequences) can integrate into replicating viral genomes. These findings show that viral populations carry a measurable load of host DNA sequences, further supporting the role of viruses as vectors of horizontal transfer of DNA between insect species. The potential uncontrolled gene spread associated with the use of viruses produced in insect cells as gene delivery vectors and/or biopesticides should therefore be evaluated.

## Introduction

The genomes of large eukaryotic double-stranded DNA viruses contain high proportions of cellular genes resulting from host-to-virus horizontal transfers (HT) [1–4]. For example, at least 10% of giant virus genes and up to 30% of herpesvirus genes likely originated from eukaryote or prokaryote genomes [1, 5, 6]. Some of these genes have been shown to act as key factors in the etiology of viral diseases [7, 8]. Because cellular gene content can be quite different between closely related viruses and/or quite similar between distantly related viruses [1, 2, 9], viral co-option of host genes appears to be rather frequent during virus evolution. The cellular genes that have so far been identified in viral genomes result from relatively ancient host-to-virus HT events. From a population genetics perspective, these viral-borne host genes must have been inherited at low to intermediate frequencies over multiple rounds of viral replication until they finally reached fixation in the viral species, likely because they provided a fitness gain to the virus. In agreement with this hypothesis, many of these genes are thought to play a role in thwarting host anti-viral defenses, thus facilitating viral replication [10]. A corollary of this scenario is that many viral-borne host genes resulting from host-to-virus HT should be found at varying frequencies in viral populations. However, host-to-virus HT has never been investigated at the micro-evolutionary scale of the viral population. Therefore, the frequency of host-to-virus HT as well as the evolutionary and molecular processes underlying the capture and domestication of eukaryotic genes by viruses remain poorly understood.

Another outstanding question arising from host-to-virus HT is whether viral-borne host genes acquired from a given host individual can be transferred to the genome of another infected individual through virus-to-host HT. In other words, can viruses act as vectors of HT between their hosts? Hundreds of HT cases have been characterized in eukaryotes [11, 12]. Many of these transfers have generated evolutionary novelties and allowed receiving organisms to adapt to new environments [13–15]. Horizontal transfer of DNA is therefore increasingly appreciated as an important evolutionary force shaping eukaryote genomes. However, the mechanisms and the potential vectors involved in HT of DNA between eukaryotes remain poorly known, especially in multicellular eukaryotes. Viruses have been proposed as candidate vectors facilitating HT between eukaryotes because they are transmitted horizontally (and in some cases vertically) and they replicate inside host cells [16, 17]. In metazoans, the vast majority of HTs characterized so far are transfers of transposable elements (TEs), which constitute pieces of DNA that are capable of moving from one genomic locus to another, often duplicating themselves in the process [18]. Several studies have uncovered host TEs packaged in viral capsids or even integrated into viral genomes, suggesting that TEs can jump from host to virus during the course of a viral infection [19–24]. We discovered two such TEs from the cabbage looper moth (*Trichoplusia ni*) integrated at low frequency in genome populations of the baculovirus *Autographa californica multiple nucleopolyhedrovirus* (AcMNPV) following infection of *T*. *ni* caterpillars [23]. Importantly, these two TEs show signs of HT between several sympatric moth species that can be infected by baculoviruses in the wild. The search for *T*. *ni* sequences integrated into populations of AcMNPV was however restricted to the dozen of *T*. *ni* genes and transposable elements that were known at the time. Therefore, the number and diversity of moth TEs and non-TEs that become integrated into AcMNPV genomes during the course of an infection remains poorly characterized.

Here we report a comprehensive search for host sequences integrated in 21 genome populations of the baculovirus AcMNPV (*Baculoviridae*) following infection of caterpillars from two moth species. The *Baculoviridae* comprise large, circular dsDNA viruses infecting mainly Lepidoptera but also Hymenoptera and Diptera [25]. Most baculoviruses are transmitted as occlusion bodies (OBs), i.e. the virions are protected in a protein matrix allowing the virus to remain infectious in the environment for extended periods of time [26]. AcMNPV is a multiple nucleopolyhedrovirus, meaning that each OB typically contains dozens of virions, each enclosing multiple genomes individually packaged within nucleocapsids. This morphology allows the virus to initiate infection as a highly polymorphic population [27], and can foster the maintenance of deleterious genotypes [28]. Rather untypical for a baculovirus, AcMNPV is a generalist virus, able to infect moth species belonging to nine lepidopteran families [29]. The two moth species we used are *Trichoplusia ni* (Plusiinae) and the beat armyworm *Spodoptera exigua* (Noctuinae), which belong to the Noctuidae family and are known to be highly susceptible to AcMNPV. These agricultural pests are found in many regions of the world, and can occur in sympatry [30]. We performed *in vivo* experimental infections of both *T*. *ni* and *S*. *exigua* caterpillars to generate AcMNPV populations for deep sequencing. Our population genomics approach yields the first estimate of the frequency and spectrum of host sequences that can become integrated in the genome of a large dsDNA virus.

## Results and Discussion

### Number of moth sequences in populations of the baculovirus AcMNPV

The first AcMNPV genomic dataset we analyzed was generated by sequencing the 134-kb AcMNPV genome at 187,536X average depth after *in vivo* amplification of the virus in *T*. *ni* larvae (G0 in S1 Fig). The viral population that produced this dataset was then independently passaged in ten lines of *T*. *ni* larvae and ten lines of *S*. *exigua* larvae, each line consisting of ten successive infection cycles (G10 in S1 Fig). OBs recovered from the last infection cycle of each line were sequenced at between 9,211X and 33,783X average depth for the ten *T*. *ni* lines (total depth = 145,386X) and between 3,497X and 35,434X average depth for the ten *S*. *exigua* lines (total depth = 163,610X). Viral sequencing reads were used as queries to perform Blastn searches against sequences from both moth species. Host sequences included RNAseq data corresponding to 70,322 *T*. *ni* contigs and 96,675 *S*. *exigua* contigs [13,14], as well as 469 and 486 contigs from *T*. *ni* and *S*. *exigua*, respectively, that were assembled in this study using sequencing reads that did not map onto the AcMNPV genome (see methods). All viral reads aligning to moth contigs were then used as queries for Blastn searches against the AcMNPV consensus genome [9] to identify chimeric reads (i.e. sequences containing both AcMNPV and moth DNA), as evidence of junctions between host and viral DNA. After applying various filters to eliminate false positives, we extracted a total of 27,504 chimeric reads from all 21 AcMNPV genomic datasets. Chimeric reads were identified in the initial AcMNPV population from *T*. *ni* (n = 9,464), as well as in all ten *T*. *ni* lines (n = 460 to 1,904; total = 12,219) and all ten *S*. *exigua* lines (n = 41 to 1,684; total = 5,821) (S1 Table; S1 Dataset).

### Nature of moth sequences integrated into AcMNPV genomes

The 27,504 host-virus DNA junctions involved 38 *T*. *ni* and 48 *S*. *exigua* contigs (S1 Table; S2 Dataset). Similarity searches and structure analyses revealed that 69 of these contigs are TEs (29 in *T*. *ni* and 40 in *S*. *exigua*) belonging to both major groups of eukaryote TEs (Table 1; S1 Table): retrotransposons (three superfamilies) and DNA transposons (10 superfamilies). The remaining 17 contigs did not show any sequence or structural similarity to any known TE or protein. However, their closest Blastn hits in the GenBank whole-genome sequence database were found in Lepidoptera, suggesting these 17 contigs indeed originate from the host genomes.

**Table 1:** Numbers and frequencies of moth sequences inserted in AcMNPV baculovirus genome populations. *T*. *ni* G0: initial AcMNPV genome population sequenced after amplification of the virus in larvae of the cabbage looper (*Trichoplusia ni*). *T*. *ni* G10 and *S*. *exigua* G10: AcMNPV populations obtained after 10 infection cycles of the virus on 10 lines of *T*. *ni* and 10 lines of the beet armyworm (*Spodoptera exigua*). G10 populations were obtained by sequencing viral genomes produced during the 10^th^ infection cycle of each of the 10 *T*.*ni* and *S*. *exigua* lines. TE: transposable element.

Type of moth sequence	*T*. *ni* G0	*T*. *ni* G10	*S*. *exigua* G10
**DNA transposons**			
Harbinger	4415	11708	2037
hAT	5	5	21
Helitron	0	0	9
Mariner	2390	40	70
MuDR	2	4	0
MULE	0	0	237
P	66	23	0
Piggybac	324	45	2734
Sola	1917	173	204
Transib	178	37	0
**Retrotransposons**			
BEL/Pao	3	2	9
Copia	136	137	43
Gypsy	2	6	299
**TE undetermined**	6	12	0
**undetermined**	20	27	158
**Total**	9464	12219	5821
**Insertion frequencies**			
Mean	3.3%	7.1%	2.6%
Lowest	*NA*	3.8%	1.1%
Highest	*NA*	14.3%	7.2%

The large proportion of TEs among the host contigs found to be joined to viral DNA may indicate that transposition is the main mechanism of insertion of host DNA into viral genomes. Alternatively, junctions between host TEs and viral DNA could result from technical artifact leading to chimeric reads composed of viral and contaminating host sequences. Though we verified that the amount of contaminating host DNA was very low (if at all present) in all our samples (see ref [23] and S1 Text), we cannot totally exclude the presence of such contamination. If our samples were contaminated, and given that TEs make up the single largest fraction of eukaryote genomes [31], technical chimeras involving mainly host TEs might not be unexpected. However, the 27,504 chimeric reads correspond to 7,049 different junctions, as defined by their location in the viral genome and in host contigs. Indeed, 1,412 of these 7,049 unique junctions are covered by more than one read (two to 1,256 reads; S2 Fig). This strongly suggests the junctions we observe do not result from any kind of technical artifact. As duplicates generated by PCR during library construction were removed (S1 Text), it seems very unlikely that a technical error would generate multiple chimeras involving exactly the same virus and host DNA sequences at the same positions. Only amplification of junctions through *in vivo* viral replication provides a plausible explanation for these observations, ruling out the possibility that these junctions result from technical chimeras. To further assess the biological origin of host-virus DNA junctions, we sought to characterize the molecular mechanisms involved in the integration of host sequences in AcMNPV genomes.

### Mechanisms underlying integration of moth sequences into AcMNPV genomes

**Fig 1:**
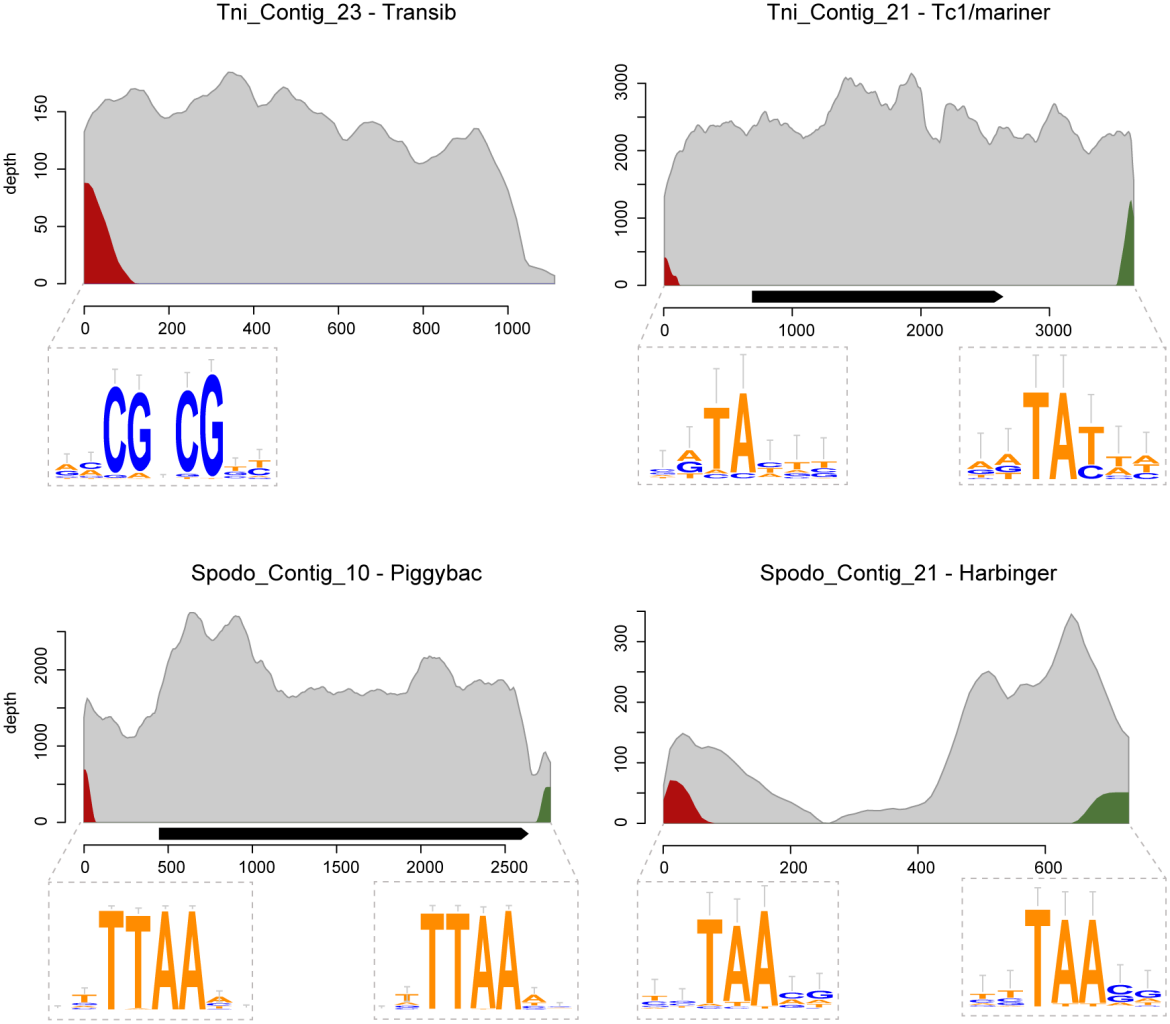
Sequencing depth (number of reads covering a position) along four host TEs found inserted into genomes of AcMNPV populations infecting *T*. *ni* or *S*. *exigua* moths. Grey curves represent sequencing depth by reads composed of host TE sequences only. Red curves represent chimeric reads whose right parts are composed of TE sequences (the left part being viral sequences) and green curves represent reads whose left parts are composed of TE sequences. Right and green curves thus respectively represent sequencing depths at junctions involving the left and right ends of a TE. The junctions at each end result from transposition at many viral sites, for which a sequence conservation logo is shown. Conserved bases correspond to known target sites of TE families (which are specified next to the host TE names). Black arrows indicate the locations and orientations of putative transposase genes along TEs. Sequencing depth of other moth contigs and sequence conservation logos of other host-virus junctions are provided in S3 and S4 Figs.

For each host contig integrated into several distinct viral sites, we located the target insertion sites both in the viral genome and in the host sequence, and examined sequence patterns in their vicinity. We found that most inserted sequences were DNA transposons, for which the junctions with the virus genome clustered immediately before the 5’ terminal inverted repeat (TIR) or immediately after the 3’ TIR, as expected in the case of transposition events (Fig 1; S3 and S7 Figs; S3 Dataset). In addition, the integration sites in the viral genome were generally characterized by short (1–5 bp) highly conserved sequence motifs (Fig 1; S4 Fig) corresponding to known TE preferred insertion sites (e.g., TTAA for the Piggybac family, TAA for harbinger, CGNCG for transib). These patterns corroborate earlier findings [19–21, 23] and indicate that many DNA transposons are indeed able to integrate into viral genomes during the course of an infection through *bona fide* transposition. Overall, we identified 19,899 host-virus junctions resulting from transposition. Counting only once all host-virus junctions covered by more than one read yields a minimum of 6,579 junctions resulting from independent transposition events, out of 7,049. The mechanism underlying the vast majority of the host-to-virus HT detected in this study is therefore transposition.

**Fig 2:**
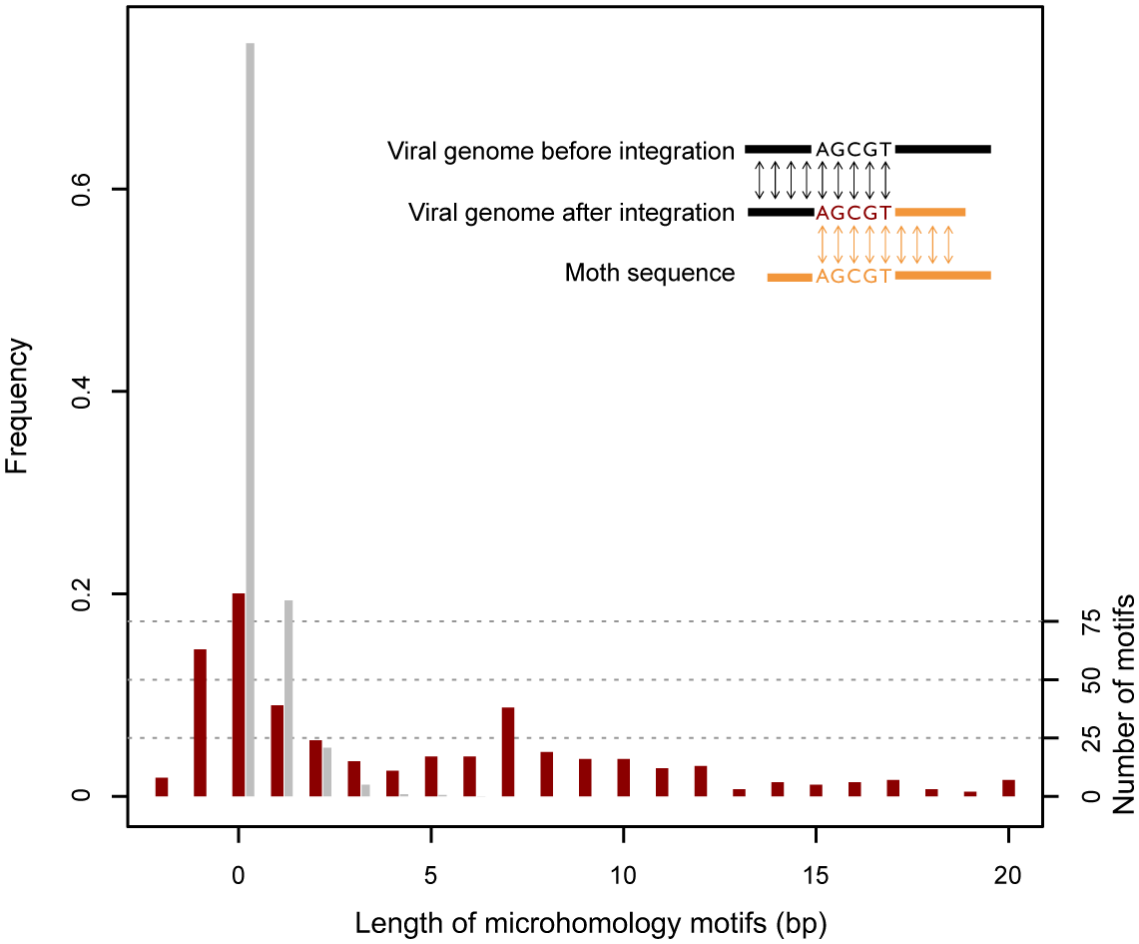
Length distribution of microhomology motifs found at 434 junctions between moth and AcMNPV baculovirus sequences. The observed distribution is shown in red and the distribution expected by chance is shown in grey. An example of a five base-pair microhomology motif between an integrated moth sequence and the AcMNPV genome is shown at the top right corner of the graph. Negative microhomology lengths correspond to junctions characterized by the presence of 1 to 2 nucleotides that did not originate from either the host or viral genomes.

Among the remaining insertions, 434 unique host-virus junctions were deemed highly unlikely to result from transposition. Contrary to the host-virus junctions resulting from transposition, which were all located at the extremities of the 5’ and 3’ TIRs, these junctions were scattered within the host sequences. A short sequence motif of 1 to 20 bp, identical between the insertion site and the host sequence, characterized 298 of these junctions (Fig 2). The length of these microhomology motifs is significantly longer than expected by chance (Khi^2^ = 4,523; *p* < 10^−15^, 20 d.f.) and argues against technical artifact as the main cause of these junctions (as technical error favoring microhomology are highly unlikely). We also note that 15 of the 434 junctions are covered by more than one read (two to 27 reads), indicating that some of these junctions were amplified through viral replication. The 158 remaining non-transposition junctions lacking microhomology could either have resulted from the ligation of blunt-ended sequences (n = 87), or were characterized by the presence of 1 to 2 nucleotides that apparently did not originate from either the host or viral genomes (n = 71, corresponding to negative microhomology lengths in Fig 2). The distribution of microhomology lengths suggests that in addition to transposition, host DNA can be integrated into viral genomes via a variety of recombination events, some of which (but not all) rely on microhomology motifs between virus and host DNA sequences. Whether such recombination events are mediated by viral factors (e.g. LEF-3, AN, PCNA [32, 33]), or by host-encoded DNA repair mechanisms [34] known to enhance baculovirus amplification [35], would be worth addressing at the functional level in the future. Most of the non-transposition junctions lie within DNA transposon sequences, which may appear intriguing. We speculate that on principle, any region of the host genome could be joined to viral DNA through microhomology-mediated recombination (including genes that may turn to be beneficial to the virus), provided that it contains a double stranded break. Yet, since DNA transposons have the capacity to excise themselves from the host genome, they may be among the most numerous extra chromosomal DNA fragments containing double stranded breaks ready to recombine with broken viral DNA.

### Distribution of moth sequences along the AcMNPV genome

**Fig 3:**
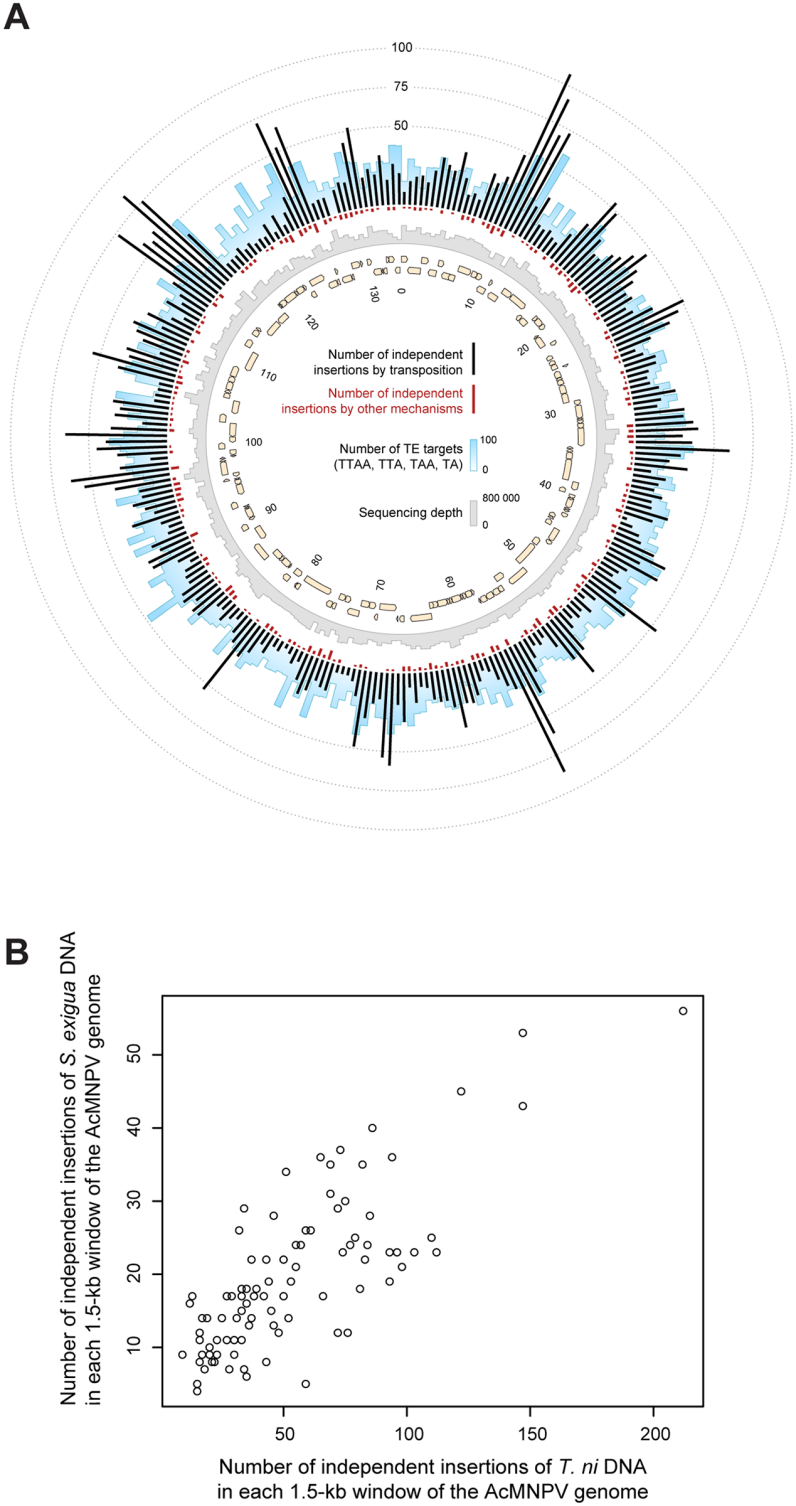
Patterns of moth DNA sequence integration along the circular AcMNPV baculovirus genome. A. Distribution of independent moth sequence integrations through transposition (black bars) and microhomology-mediated recombination (red bars) in 500-bp contiguous windows. The blue and grey bar plots respectively illustrate the number of the most frequent transposon target motifs (TTAA, TAA, TTA, TA) and the average sequencing depth in these windows. Beige arrows represent AcMNPV genes. B. Correlation between the numbers of *T*. *ni* and *S*. *exigua* sequences integrated by transposition in 1500-bp contiguous windows of the AcMNPV genome. Each point on the plot represents a window.

We then mapped the independent insertions of host sequences along the AcMNPV genomes, that is, counting only once all insertions possibly resulting from amplification through viral replication. The map (Fig 3A; S5 Fig) shows that integrations occur virtually everywhere in the viral genome and that all 151 viral genes are disrupted by host insertions at least once. Remarkably, the local densities of insertions of *S*. *exigua* TEs strongly correlate to those of *T*. *ni* TEs (Fig 3B and S2 and S3 Tables; 54% of variance explained; *p* < 10^−15^). This correlation is not explained by variation in sequencing depth or density of the preferred transposition motifs (identified above) along the viral genome (S2 and S3 Tables). Two other causes may explain this correlation: (1) varying degrees of tolerance to insertions along the viral genome and (2) varying rates of transposition along the viral genome. Cause (1) implies that insertions are more likely to be replicated through viral replication in some regions than others because their impact on viral fitness would be lower. In this case, the correlation between local densities of insertions along the viral genome should be maintained or even higher when considering all insertions that possibly result from viral replication. However, the correlation almost disappears (0.1% of variance explained, S3 Table) when all insertions (including potentially replicated ones) were considered. Hence, although the fitness impact of insertions may well vary along the viral genome, these variations cannot explain the strong correlation we initially observed. This leaves cause (2) as the only explanation for the correlation of insertion frequencies from TEs of the two species. In other words, TEs from the two moths, in spite of being different (Table 1 and S1 Table), tend to transpose preferentially into the same regions of the AcMNPV genome irrespective of the density of target sites. This suggests that the pattern of integration may be shaped by structural properties of the viral genome. We propose that, as observed in eukaryotic genomes [36–38], the distribution of transposition-mediated integrations along the AcMNPV genome may be influenced by accessibility of the viral genome to host TEs, which itself likely depends on the structure of AcMNPV chromatin, known to be dynamically remodeled during viral replication [39].

### Frequency and inheritance of moth sequences integrated into AcMNPV genomes through infection cycles

Taking into account the number of chimeric reads per library, the total number of reads in each library, the size of the AcMNPV genome and the minimum alignment size that can be returned by Blastn, we calculated that on average 4.8% viral genomes (range 1.1% to 14.3%) carry at least one host sequence among AcMNPV populations (Table 1). Though the number of host-to-virus HT events that generated these frequencies is likely to be high, we cannot infer it precisely because we cannot tell how many of the host-junctions covered by more than one read were amplified through viral replication. Indeed, the same TE may insert several times at any suitable viral site. Furthermore, it is possible that a number of host-virus junctions, which cannot be evaluated here, were generated through subsequent transposition of viral-borne TEs (not coming from the host genome) into multiple copies of the virus genome.

The relatively high frequencies of AcMNPV genomes carrying host DNA fragments at any given time raise the question of whether such host sequences are inherited over viral infection cycles. We thus tested the residual presence of *T*. *ni* sequences (inserted at G0 in S1 Fig) in viral populations, that had subsequently been passaged 10 times in *S*. *exigua* (G10 datasets in S1 Fig). Using the libraries from *S*. *exigua* G10 viruses as Blastn queries against *T*. *ni* contigs revealed 24 new insertions that were not previously found in Blastn searches against *S*. *exigua* contigs (see S1 Text for details). These insertions likely involve *S*. *exigua* sequences homologous to *T*. *ni* but absent from the *S*. *exigua* contigs. None of these insertions were identical (in terms of position in the virus genome and host contig) to any found in the G0 virus population. The persistence of a given host DNA fragment in virus populations thus appears to be low, likely because of the deleterious effects large insertions have on the viral genome carrier. Although many new host sequences become integrated into AcMNPV genomes at each viral infection cycle, they are thus purified out of the viral population after only few infection cycles. Hence there is a high turnover of host sequences inserted into the viral genome each time the virus replicates in a host. Under natural settings, continuous host-to-virus flow of genetic material generates a significant proportion of recombinant viruses (Table 1). At the viral population scale, this represents a gene reservoir that could fuel host-virus coevolutionary arms race through co-option of a host sequence favoring the virus in a given environment. Our findings thus shed light on the first evolutionary steps underlying viral co-option of cellular genes.

### Virus-mediated horizontal transfer of moth transposable elements

Under the hypothesis that viruses can act as vectors of HT of TEs, once inserted in a viral genome, viral-borne TEs should then be able to jump from the viral genome to the genome of a new host organism. To evaluate the possibility that AcMNPV can shuttle TEs between insects, we first checked whether some TEs found integrated in our AcMNPV genome datasets have retained the structural features necessary for transposition. Among the 41 contigs inserted in at least 10 different viral sites, 11 correspond to TEs for which we recovered both TIRs and that encode a putative full-length intact transposase gene (Fig 1; S3 Fig). Provided that these TEs can be transcribed in a new host, they should thus be able to jump from the viral genome into the genome of this new host. However, it is important to note that the transfer would only be effective if the host survived viral infection in the first place. This is more likely to happen if the host shows resistance to the virus or if the virus harbors deleterious mutations.

**Fig 4:**
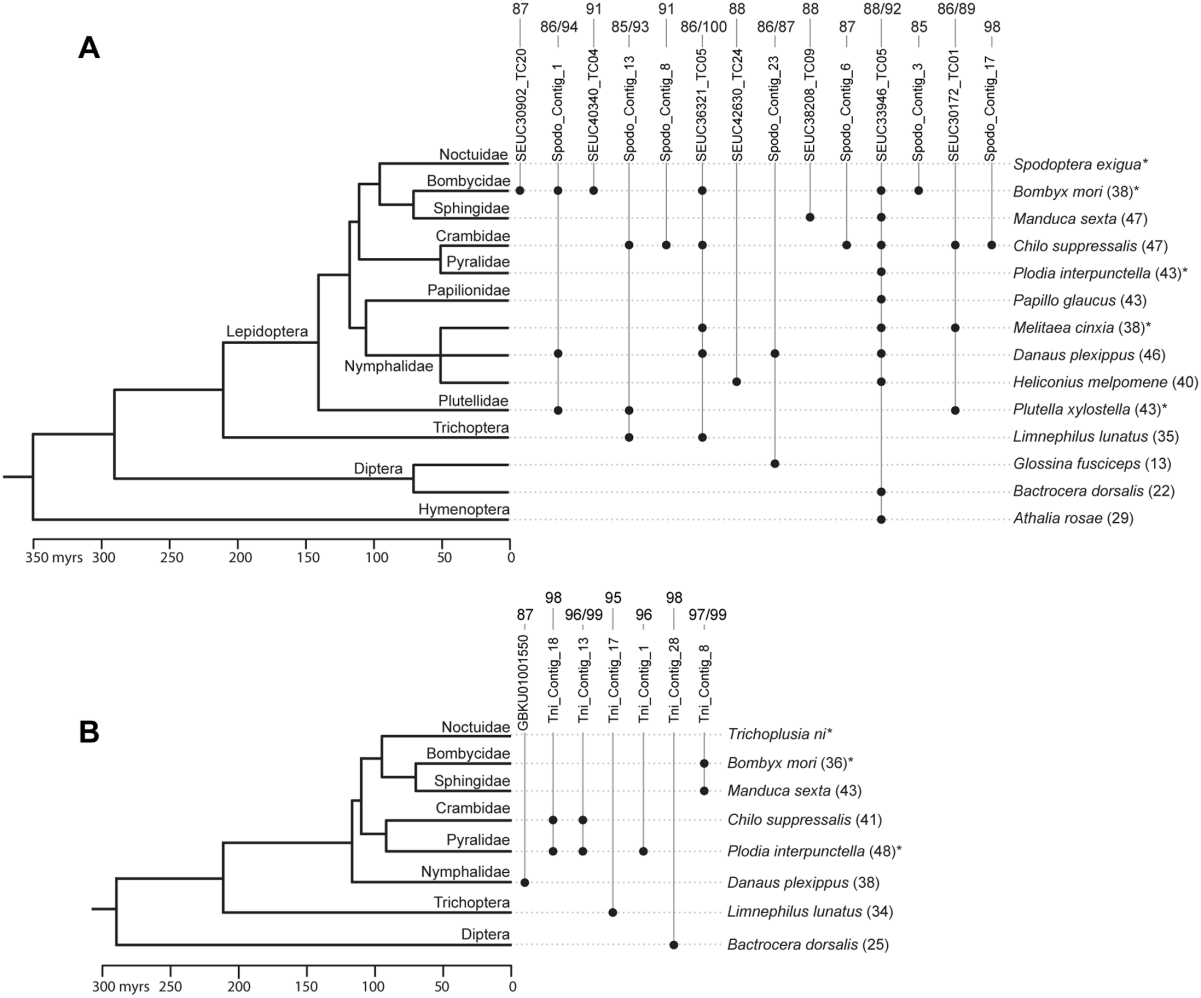
Timetree of various insect species in which we found evidence for horizontal transfer of *Spodoptera exigua* (A) or/and *Trichoplusia ni* (B) transposable elements (TEs) found integrated in one or more AcMNPV populations. Names of contig containing TEs correspond to those in S4 and S5 Tables. Black dots indicate that we have found a Blastn hit aligning with at least 85% nucleotide identity over at least 100 bp to a *S*. *exigua* or *T*. *ni* TE. For example, the figure shows that the *S*. *exigua* contig called Spodo_Contig_23 (which is a *piggybac* TE according to S4 Table) was horizontally transferred between *S*. *exigua*, *Danaus plexipus* and *Glossina fusciceps*. Numbers on top of contig names indicate the level (or range) of nucleotide identity between each *S*. *exigua* or *T*. *ni* TE and their Blastn hit(s) in other species (in percentages). Numbers between brackets at the right of taxa names are the average percent similarities for 11 conserved genes between *S*. *exigua* or *T*. *ni* and the other species. These percent similarities are derived from synonymous distances (dS) calculated for each gene and are equal to (1 –dS) × 100. All distances are provided in S4 and S5 Tables. Divergence times were taken from refs [44–46]. Divergence times between Nymphalidae species are unknown and were set arbitrarily at 50 million years for illustrative purposes. *Species known to be susceptible to AcMNPV [29].

We then reasoned that if AcMNPV is able to act as vector of HT of TEs between its insect hosts, the TEs we found integrated in the AcMNPV genome populations might have been horizontally transferred relatively recently between insects of various susceptibility to AcMNPV. To test this hypothesis, we assessed whether some of the TEs uncovered in this study have been horizontally transferred between *T*. *ni* and/or *S*. *exigua* and other insect lineages. We used the 69 TE sequences as queries to perform Blastn searches against the 144 non-Noctuidae insect genomes available in GenBank as of March 2015. For 21 of these TEs (14 *S*. *exigua* and seven *T*. *ni* TEs), we found highly similar copies (>85% nucleotide identity) in the genome of one or more other insects (Fig 4). The 21 TEs show a combination of features that are typically indicative of HT [40–42]. They have a patchy distribution in the insect phylogeny and, importantly, the between-species nucleotide identity calculated for each of these TEs is much higher (91% identity on average) than synonymous nucleotide identities calculated for 11 conserved genes between the same species (37% identity on average, Fig 4; S4 and S5 Tables). We conclude that at least 21 of the 69 TEs found integrated in AcMNPV have undergone one or multiple horizontal transfers between *T*. *ni* or *S*. *exigua* and one or several other insect lineages. Nucleotide identity between some *T*. *ni* or *S*. *exigua* TEs and those uncovered in other insects is very high (up to 99%), suggesting some of HT events took place very recently. Among the other insects involved in HT, we found four lepidopteran species known to be susceptible to AcMNPV infection (Fig 4) [43]. Our study therefore provides further compelling support for the role of baculoviruses as potential vectors of TEs between lepidopterans [19, 20, 23]. Indeed, given that on average 4.8% of baculovirus genomes harbor a host sequence and that a caterpillar typically becomes infected by ingesting thousands of baculovirus genomes, each non-lethal infection represents an opportunity for between-host baculovirus-mediated transfer of DNA.

### Conclusions

In this study, we have shown that each time the baculovirus AcMNPV infects a caterpillar host, a large number of host TEs can transpose into its genome. Many TEs and other host sequences can also integrate into AcMNPV genomes through microhomology-mediated recombination events. Our work also demonstrates that the density of transposition events is not homogenous along the AcMNPV genome and that, while the influx of host sequences integrated into AcMNPV is continuous, each newly integrated host sequence is rapidly purged out of AcMNPV populations. Together, these observations are reminiscent of the well-known gene exchanges that take place between bacteria and bacteriophages [47], and indicate that such exchanges may also occur on a regular basis between eukaryotes and eukaryotic viruses. Our results also raise a number of questions worth addressing in future experiments. In particular, it would be interesting to monitor the evolution of the frequency of viral replicates carrying any given host sequence across successive infection cycles, as the host DNA sequence retention time affects the likelihood of such sequence being horizontally transferred between hosts. Furthermore, since the rate of host-to-virus HT is measurable at the population level, it would be worth investigating whether this phenomenon influences the within-host replication dynamics of AcMNPV. Another exciting question is whether this phenomenon is limited to moth-AcMNPV interactions or whether it also takes place in other host-virus systems. Finally, it is noteworthy that AcMNPV and other baculoviruses are used as biopesticides and developed as vectors for several biomedical applications such as gene or vaccine delivery [48, 49]. Our results therefore call for an evaluation of the risk of gene or TE spread through uncontrolled virus-mediated HT potentially generated by these approaches, which rely on mass production of the viruses in insect cells or *in vivo*.

## Materials and Methods

### Viral population genomics data sets

The GenBank accession numbers of the 21 AcMNPV genomic datasets analyzed in this study are: SRS533250, SRS534469, SRS534534, SRS534575, SRS534677, SRS534587, SRS534590, SRS534631, SRS534673, SRS536572, SRS536571 and SRS534470, SRS534499, SRS534514, SRS534536, SRS534537, SRS534543, SRS534542, SRS536937, SRS534588 and SRS534589 [23]. They consist of 101-bp paired sequences (reads), except for dataset SRS533250, which consists in 151-bp paired reads.

These datasets were produced through experimental evolution, which consisted in generating ten *per os* infection cycles on ten lines of *T*. *ni* and *S*. *exigua* larvae using 2500 occlusion bodies from an AcMNPV stock derived from a viral sample originally isolated from a single Alfalfa looper (*Autographa californica*) individual collected in the field. The full experiment is described in Gilbert, Chateigner [23] and in S1 Fig. The AcMNPV DNA samples used to produce the 21 sequencing datasets were all extracted using the QIAamp DNA Mini Kit (Qiagen) after purification of AcMNPV occlusion bodies by a percoll-sucrose gradient, Na_2_CO_3_ dissolution and enzymatic removal of host DNA [23].

Data analyses were performed in R [50], unless another tool is mentioned. All Blastn searches were carried out under default settings.

### Assembly of non-viral sequences

To identify host DNA sequences integrated in genomes of the AcMNPV baculovirus, we used viral reads as queries to perform Blastn searches on *T*. *ni* and *S*. *exigua* transcripts generated by Pascual, Jakubowska [51] and Chen, Zhong [52]. In addition, to recover as many insertions of host DNA as possible, we assembled non-viral DNA elements present in the viral genomic libraries. These elements may represent inserted host DNA sequences absent from (or incomplete in) the available transcriptomes of both moth species.

We applied the following procedure on genomic libraries obtained from each moth species. We aligned all reads on the AcMNPV genome using the end-to-end mapping strategy of Bowtie 2 [53]. We used Samtools view on resulting alignment files to extract read pairs for which at least one read did not align. These unmapped reads were trimmed off low quality score bases with Trimmomatic [54], and assembled with SOAP deNovo 2 [55] using a kmer length of 71 bases, which showed good assembly statistics compared to other lengths.

We checked assembly quality by performing Blastn homology searches of assembled contigs against themselves, and found that many contigs differed only at one or both of their ends but were otherwise identical. Blastn searches of the contigs against the AcMNPV genome revealed that the contig ends that differed were similar to parts of the viral genome. We assumed that mostly similar contigs resulted from a genetic element inserted at different sites of the AcMNPV genome, and that these viral sites had been partly included into contig ends during the assembly process. We thus trimmed contigs from these viral regions, and reassembled them using the assembly feature included in Geneious 4.5 [56], allowing a maximum mismatch of 10% in overlapping regions. This yielded 469 contigs for genomic libraries generated from *T*. *ni* lines and 486 contigs for *S*. *exigua* lines. These contig sequences were added to known transcriptome sequence of the corresponding moth species [51, 52], which we hereafter simply refer to as “transcripts”, in order to constitute the host databases for the Blastn searches designed to identify junction between moth and viral DNA.

### Detection of junctions between host and virus DNA

Blastn searches with default parameters [57] were carried out using the 21 AcMNPV genomic datasets as queries to identify similarities of at least 28 nucleotides (as defined by the default settings) between reads obtained from each viral line and the sequence database corresponding to its host. Each read showing similarities was then blasted against the AcMNPV reference genome, together with the other read of its pair (mate) so as to detect junctions occurring between paired reads.

For a given read listed in a blast output, we retained the alignment with the best score, randomly choosing between alignments of identical scores. This selection was done separately for alignments with transcripts and for alignments with contigs, in order to help selecting between homologous contigs and transcripts (see below). For a read to be considered as a junction between host and virus DNA, we imposed minimum lengths of alignment with the virus genome *only*, and with the host genome *only*, of 16 bp each (S6 Fig). Furthermore, at least 95 nucleotides of the read had to align with virus and host sequences (130 bp for 151-bp reads). The overlap between these alignments was set to involve at most 20 bp and at least -2 bp. These filters excluded reads from virus regions having similarities with host contigs, in which case the region aligning to a host contig would be included in that aligning to the virus genome.

To detect junctions that occurred between two paired reads (mates), we selected read pairs meeting the following conditions: (i) one mate must have a similarity with the virus genome of at least 95 bp (130 bp for 151-bp reads) and present no similarity with any host contig, and (ii) the other mate must have a similarity to a host contig of at least 95 bp (130 bp for 151-bp reads), and present no similarity with the virus genome.

We found that the sensitivity of our approach to detect junctions between host and virus DNA was highly dependent on the quality of the assembly of the host sequences that were used as reference. Thus, it will be important that future studies dedicate substantial effort to generate a high quality and comprehensive set of host sequences in order to find all possible host-virus chimeras.

### Selection of host sequences

We discarded all alignments (junctions) involving contigs or transcripts not meeting the following criteria. To ensure that a contig we assembled represented moth DNA, it had to be partly similar to a transcript of the corresponding host species, as determined by Blastn searches of contigs against host transcripts, or to align with at least one read of a pair that also aligned with a known host transcript, as determined by the Blastn search of reads against the contig and transcript databases.

Because the contigs we assembled are, as expected, partly similar to some host transcripts, a chimeric read may have similarities to a contig and to a transcript (after selecting the best alignment in each category, as explained above). In other words, contigs and transcripts can be candidates for the same insertions. To minimize redundancy between contigs and transcripts, any transcript sharing at least one chimeric read with a contig was discarded. We therefore retained transcripts that did not share any junction with any assembled contig. Finally, we discarded host contigs or transcripts having similarities with less than three chimeric reads (i.e., potentially inserted in the AcMNPV genome less than three times) or having a cumulative alignment length of less than 75 bp with chimeric reads.

For junction counts shown in S1 Table, we removed duplicates that may have resulted from PCR amplification of the same junction during library preparation (S1 Text). Junctions sequenced in both directions and appearing in two overlapping paired reads were counted only once.

### Frequency of insertions of host DNA into viral genomes

Defining *P*_*j*_ as the average number of junctions per virus genome involved in the construction of a genomic library, the probability for a read from that library to cover a junction between a host sequence and the viral genome can be approximated as *P*_*j*_ × *L*_*r*_/*L*_*g*_, where *L*_*r*_ is the read length and *L*_*g*_ is the length of the virus genome. For a read to be chimeric under our criteria, a junction has to be at least 28 bp away from the read ends (S6 Fig). However, the overlap between alignments at the junction (S6 Fig), the mean length of which we denote as *Ov*, allows the junction to be slightly closer from the read end and to yield a 28-bp region of sequence similarity detectable by blast, so that the probability for a read to be chimeric is $P_{j}\;\times \frac{L_{r}-56+Ov}{L_{g}}$.

Since this probability can be approximated as the ratio of the number of chimeric reads *N*_*c*_ over the number of viral reads *N* of a sequence library, we obtain $P_{j}≅\frac{N_{c}}{N}\;\times \;\frac{L_{g}}{L_{r}-56+Ov}$.

*N* was estimated by running samtools view [58] on alignment files obtained by mapping reads from each virus line on the AcMNPV consensus genome, using the local sensitive settings of Bowtie 2 [53]. *N*_*c*_ includes technical duplicates (PCR duplicates and overlapping paired reads, see above) because they contribute to *N* as much as they do to *N*_*c*_.

We derived the proportion of virus genomes carrying at least one host DNA fragment by assuming that the number of inserted fragments per virus genome follows a Poisson distribution of mean *P*_*j*_/2, as one insertion of host DNA into the circular AcMNPV genome should yield 2 junctions.

### Characterization of junctions and insertion types

For simplification, we hereafter refer to contigs assembled in this study and to previously assembled host transcripts as “contigs”.

We identified each junction producing a chimeric read by the offset it involves between the virus genome coordinates and the host contig coordinates (S1 Text). Reads having the same offset, involving the same viral DNA strand, host contig, and coming from the same genomic library in the case of *S*. *exigua* lines (which do not share a ancestor on this host) were considered likely to come from viral amplification of the same original insertion of host DNA. In the following analyses, we selected only one chimeric read per original junction, favoring the read with best alignment score on the host contig.

These reads were mapped onto their corresponding contig by inserting gaps of appropriate length before their sequence, based on alignment coordinates reported by blast, to produce multifasta alignment files. Visualization of these files in Geneious [56] and BioEdit [59] (example shown in S7 Fig) showed that junctions clustered at one or two positions in a contig likely representing the end(s) of a TE. For many contigs, this was further supported by the presence of terminal inverted repeats (TIRs), which are typical of class II DNA transposons, the presence of long terminal repeats (LTRs), which are typical of LTR retrotransposons and by similarities to known TE protein motifs returned by blastx on the GenBank non-redundant protein database.

For each cluster of at least 10 junctions, which likely represent insertions of the same TE end, we analyzed sequence conservation at insertion sites in the virus genome. This was done by computing insertion coordinates based on the previously obtained offset (S1 Text), and by building sequence conservation logos [60–62] of 30-bp sequences around insertion sites. Sequence logos are provided in S4 Fig.

Some junctions did not form clusters (according to our criteria defined in S1 Text) and were scattered along host contigs (example shown in S7 Fig), suggesting that different fragments of these contigs were inserted. This concerned 434 junctions, most of which presented similarities between host and virus sequences at insertion points (Fig 2, yielding to the overlap shown in S6 Fig). To check whether these similarities were overall longer than expected by chance, we extracted from each chimeric read resulting from this type of junction the last 20 bp that aligned to the host contig (next to the junction point), and computed the lengths of similarities this 20-bp sequence had with 20 random 20-bp regions of the AcMNPV consensus genome. This allowed comparing the distribution of expected and observed identity lengths with a Khi-square test.

### Patterns of insertions of host DNA along the virus genome

We explained the number of junctions in 1500-bp windows of the AcMNPV genome, combining all virus lines from *S*. *exigua*, with a generalized linear model including three covariates: the average sequencing depth in that window, the number of common TE targets found in *S*. *exigua* lines, and the number of junctions found in virus lines from *T*. *ni*, without considering interactions between terms.

Sequencing depth was estimated by running samtools mpileup [58] on mapping files obtained previously. We modified mpileup to allow greater depth than 8000. We counted the following frequent targets of *S*. *exigua* TEs, based on the logos we established previously (S2 Fig): “TTAA” (for piggybac TEs), “TTA”, “TAA” (for Harbinger TEs), and “TA” (for Mariner TEs).

A Poisson distribution was assumed for the number of junctions per genome window. We selected the best model on the basis of corrected Akaike Information Criterion (AICc) returned by the dredge function of the R package MuMIn [63] (S2 Table), and we submitted it to an analysis of deviance (S3 Table). We fitted this model twice: considering all junctions (including viral replicates), and only independent junctions based on their identifiers. In the latter case, the most likely model only included the number of junctions per window in *T*. *ni* as a covariate (S2 Table).

### Computing sequencing depth of host contigs

Sequencing depth of host contigs (Fig 1; S3 Fig) was computed by using alignments coordinates from results of Blastn search of reads against host databases (see above), using the same criteria to select a single alignment for reads having similarities with several contigs/transcripts. Depth was averaged over 20-bp sliding windows overlapping by 10 bp.

### Detection of horizontal transfers of transposable elements across insects

We assessed whether *T*. *ni* and *S*. *exigua* TEs found integrated in the AcMNPV genome underwent HT between insects. We used the *T*. *ni* and *S*. *exigua* TEs we identified as queries to perform Blastn searches against the 144 non-Noctuidae insect whole genome sequences available in GenBank as of March 17^th^ 2015. We identified candidate HT events when a *T*. *ni* or *S*. *exigua* TE aligned to a sequence from another insect genome with at least 85% nucleotide similarity over at least 100 bp. To assess the level of neutral genetic distance expected under vertical inheritance between *T*. *ni*/*S*. *exigua* and all insect species in which we found Blastn hits meeting the above criteria, we calculated synonymous distances for 11 conserved genes between *T*. *ni*/*S*. *exigua* and those insect species using the non-corrected Nei-Gojobori method in MEGA 6 [64], following Gilbert, Chateigner (23). Overall we calculated 143 pairwise synonymous gene distances between *S*. *exigua* and 13 other insect species and 55 pairwise synonymous gene distances between *T*. *ni* and five other insect species.

## Supporting Information

S1 Table Numbers of chimeric reads found in each AcMNPV sample. Accession number of contigs from *Trichoplusia ni* start with “GBKU” while *Spodoptera exigua* accession numbers start with “SEUC”. Contigs assembled in this study have names starting by either “Tni” or “Spodo”. Contigs marked with the same letter in the column “Contig redundancy” contain an identical sequence found in chimeric reads (i.e. integrated in the AcMNPV genome). These contigs are however not identical over their entire length. The number of different moth sequences given in the main text takes into account the redundancy, i.e. two contigs containing an identical sequence are counted only once. The numbers of chimeric reads given in this table include all chimeric reads covering the same host-virus junction, i.e. all insertions potentially amplified through viral replication after integration. *Indicates moth transposable elements for which we found evidence for horizontal transfer in insects (S6 Fig).
(DOCX)

S2 Table Selection of models explaining the number of junctions by transposition of *S*. *exigua* DNA in 1500-bp genomic windows along the AcMNPV genome. The upper half considers only independent transpositions; the lower half considers all transpositions, including viral replicates. An “x” in a cell indicates that a term is used in the model. The models retained correspond to the rows shown in bold.
(DOCX)

S3 Table Analyses of deviance of models explaining the number of junctions by transposition of *S*. *exigua* DNA in 1500-bp genomic windows along the AcMNPV genome. The upper half considers only independent transpositions; the lower half considers all transpositions, including viral replicates.
(DOCX)

S4 Table Gene and Transposable Element (TE) similarities between *Spodoptera exigua* and other insect species. TE similarities between *S*. *exigua* and the other insect species are given in percent nucleotide identity. Numbers in brackets next to percent nucleotide identities are the numbers of copies (or fragments of copies) longer than 100 bp found by Blastn in each insect genome and the length of the longest copy or fragment of copy in base pairs. TE numbers correspond to those in Fig 4A. Divergence times were taken from refs [44–46].
(DOCX)

S5 Table Gene and Transposable Element (TE) similarities between *Trichoplusia ni* and other insect species. TE similarities between *T*. *ni* and the other insect species are given in percent nucleotide identity. Numbers in brackets next to percent nucleotide identities are the numbers of copies (or fragments of copies) longer than 100 bp found by Blastn in each insect genome and the length of the longest copy or fragment of copy in base pairs. TE numbers correspond to those in Fig 4B. Divergence times were taken from refs [44–46].
(DOCX)

S1 Fig Overview of the experimental evolution setup, sequencing and similarity-based searches carried out in order to identify moth sequences integrated into populations of the AcMNPV baculovirus genome. (PDF)

S2 Fig Histogram showing the distribution of the number of reads covering a given junction. While most of the junctions (5,637) are covered by one read, 1,412 are covered by two to 1,256 reads.
(PDF)

S3 Fig Sequencing depth (number of reads covering a position) along all moth sequences found inserted into genomes of AcMNPV populations. Grey curves represent sequencing depth by reads composed of host sequences only. Red curves represent chimeric reads whose right parts are composed of host sequences (the left part being viral sequences) and green curves represent reads whose left parts are composed of host sequences. Black arrows indicate the locations and orientations of putative transposase (and Myb-like) genes along TEs. Note that for two contigs (Spodo_Contig_4 and 14) corresponding to Gypsy retrotransposons, chimeric reads fall within the contig sequence rather than on each extremity. This is likely due to missassembly of the retrotransposons, the contigs including only one long terminal repeat within the sequence rather than one on each extremity.
(PDF)

S4 Fig Sequence conservation patterns (logos) at insertion sites of moth sequences in the AcMNPV baculovirus genome. For each logo we indicate the name of the contig (starting with “SEUC” for *Spodoptera exigua* transcripts and “GBKU” for *Trichoplusia ni* transcripts), its nature, the position within each contig of a given end of moth sequence found integrated in the AcMNPV genome as well as the number of junctions involving this given end, i.e., the number of different integration sites of the sequence along the AcMNPV genome.
(PDF)

S5 Fig Detailed map of insertions of moth DNA along the AcMNPV genome. Arrows represent genes. Insertions of the same sequence at the same position were counted only once. Insertions of *Trichoplusia ni* sequences are in blue while those of *Spodoptera exigua* are in black. The orientation of the insertions is the same as that of the genes represented on the same side of the viral genome (sense: top; antisense: bottom).
(PDF)

S6 Fig Criteria used to define chimeric reads that represent junctions between host and virus DNA. This example shows an actual chimeric read having similarities to both the virus genome (right part of read) and a host contig (left part of read). We tolerated only minimal overlap between these similarities so as to discard reads from virus regions that happen to be similar with some host contig (in which case there would be no region aligning with the host contig *only*) or vice versa. Virus and host regions that have no similarity with the read are shown in grey. Above or below a type of alignment (or overlap) is a bar plot representing the numbers of reads observed from all possible alignment (overlap) lengths. The top left bar plot shows that most of the regions over which chimeric reads align only on host contig are longer than 16 bp. The top right bar plot shows that most of the regions over which chimeric reads align only to the viral genome are longer than 16 bp. The middle bar plot indicates that the regions including the host-virus junction over which the chimeric reads align both to virus and the host contig are mostly between -2 (2-bp deletion) and 20 bp long. The bottom bar plot indicates that most chimeric reads align either to the virus or to a host contig over 101 bp. These plots only include reads having similarities to both a host contig and the virus genome and belonging to genomic libraries generated from virus lines grown in *S*. *exigua*, which consist in 101-bp reads.
(PDF)

S7 Fig Visualization of an alignment between a host contig and chimeric reads in geneious (modified). The black segments represent regions of mismatch between the reads and the contig and grey segments represent similarities. Note the clustering of reads at the contig ends and the fact that, for each end, the same part of the reads (right OR left) aligns with the host contig. The other reads scattered between the two ends do not represent junctions by transposition.
(PDF)

S8 Fig Coordinates of alignments between a chimeric read and the virus genome and host transcriptome, as returned by blast. These were used to compute an offset that identifies a junction between host and virus DNA (see S1 Text).
(PDF)

S1 Dataset Moth sequences found integrated into AcMNPV genomes. Contig names starting with “SEUC” are from *Spodoptera exigua* while those starting with “GBKU” are from *Trichoplusia ni*.
(XLSX)

S2 Dataset Blastn score and coordinates of all 27,504 chimeric reads on the AcMNPV genome and on moth contigs. The “lineage” column indicates which population genomics dataset a given chimeric reads belongs to (TniG0: *Trichoplusia ni* G0; Tni1-10: *Trichoplusia ni* G10; Spodo1-10: *Spodoptera exigua* G10; see S1 Fig). The column “chimera_type” indicates whether the host-virus junction identified in a chimeric read pair lies within a read (intra-read) or between the two mates of a pair (inter-read). “Score” in column names refer to blast scores of reads against virus or host sequences. *Sv*, *Srv*, *Erc*, *Ec*, *Ev*, *Src* and *Sc* are positions of starts and ends of alignments returned by Blastn, as illustrated in S8 Fig. Asterisks in the “PCR” column indicate host-virus junctions that we were able to recover by PCR/Sanger sequencing.
(FAS)

S3 Dataset Alignments of chimeric reads and moth contigs for all host-virus junctions involving a moth contig inserted in at least 10 different positions along the viral genome. Contig names starting with “SEUC” or “Spodo” are from *Spodoptera exigua*.
(RAR)

S1 Text Supplementary material and methods. (DOCX)
