# Supplementary material for: Continuous Influx of Genetic Material from Host to Virus Populations
Source: PLoS Genet. 2016 Feb 1;12(2):e1005838. doi: 10.1371/journal.pgen.1005838 (PMC4735498; doi:10.1371/journal.pgen.1005838)
Supplement: S1 Table — Accession number of contigs from Trichoplusia ni start with “GBKU” while Spodoptera exigua accession numbers start with “SEUC”. Contigs assembled in this study have names starting by either “Tni” or “Spodo”. Contigs marked with the same letter in the column “Contig redundancy” contain an identical sequence found in chimeric reads (i.e. integrated in the AcMNPV genome). These contigs are however not identical over their entire length. The number of different moth sequences given in the main text takes into account the redundancy, i.e. two contigs containing an identical sequence are counted only once. The numbers of chimeric reads given in this table include all chimeric reads covering the same host-virus junction, i.e. all insertions potentially amplified through viral replication after integration. *Indicates moth transposable elements for which we found evidence for horizontal transfer in insects (S6 Fig). (DOCX) [file pgen.1005838.s001.docx]

Continuous influx of genetic material from host to virus populations

**Table S1. Numbers of chimeric reads found in each AcMNPV sample.** Accession number of contigs from *Trichoplusia ni* contig start with “GBKU” while *Spodoptera exigua* contigs start with “SEUC”. Contigs marked with the same greek letter in the column “Contig redundancy” contain an identical sequence found in chimeric reads (i.e. integrated in the AcMNPV genome). These contigs are however not identical over their entire length. The number of different moth sequences given in the main text takes into account the redundancy, i.e. two contigs containing an identical sequence are counted only once. The numbers of chimeric reads given in this table include all chimeric reads covering the same host-virus junction, i.e. all insertions potentially amplified through viral replication after integration. *Indicates moth transposable elements for which we found evidence for horizontal transfer in insects (Fig. S4).

|  |  |  |  | ***Trichoplusia ni* G10** | | | | | | | | | |
| --- | --- | --- | --- | --- | --- | --- | --- | --- | --- | --- | --- | --- | --- |
| **Accession number of host contig** | **Contig redundancy** | **Nature of contigs** | ***T. ni* G0** | **1** | **2** | **3** | **4** | **5** | **6** | **7** | **8** | **9** | **10** |
| GBKU01069730 |  | TE undetermined | 0 | 6 | 0 | 0 | 4 | 0 | 0 | 0 | 2 | 0 | 0 |
| GBKU01065423 | a | TE Piggybac | 3 | 0 | 0 | 0 | 0 | 0 | 0 | 0 | 0 | 0 | 0 |
| GBKU01065422 | a | TE Piggybac | 3 | 0 | 0 | 0 | 0 | 0 | 0 | 0 | 0 | 0 | 0 |
| GBKU01043456 |  | TE Piggybac | 3 | 0 | 0 | 0 | 0 | 0 | 0 | 0 | 0 | 0 | 0 |
| GBKU01040065 |  | undetermined | 0 | 1 | 0 | 0 | 2 | 0 | 0 | 0 | 0 | 0 | 0 |
| GBKU01035396 |  | TE Harbinger | 0 | 0 | 0 | 0 | 1 | 0 | 0 | 0 | 2 | 0 | 0 |
| GBKU01033694 |  | undetermined | 6 | 0 | 0 | 0 | 0 | 0 | 1 | 0 | 0 | 1 | 0 |
| GBKU01026194 |  | TE BEL | 3 | 0 | 0 | 0 | 0 | 0 | 0 | 2 | 0 | 0 | 0 |
| GBKU01024125 |  | undetermined | 2 | 0 | 0 | 0 | 0 | 2 | 0 | 0 | 0 | 0 | 0 |
| GBKU01024123 |  | undetermined | 1 | 0 | 0 | 0 | 0 | 1 | 0 | 0 | 0 | 0 | 1 |
| GBKU01009804 |  | undetermined | 3 | 0 | 0 | 0 | 0 | 0 | 0 | 0 | 0 | 0 | 0 |
| GBKU01001550* |  | TE Gypsy | 1 | 0 | 0 | 0 | 0 | 0 | 2 | 0 | 0 | 0 | 0 |
| Tni_Contig_1* |  | undetermined | 1 | 0 | 0 | 0 | 0 | 0 | 1 | 0 | 0 | 0 | 1 |
| Tni_Contig_2 |  | TE MuDR | 2 | 0 | 0 | 0 | 0 | 0 | 0 | 0 | 4 | 0 | 0 |
| Tni_Contig_3 |  | TE undetermined | 6 | 0 | 0 | 0 | 0 | 0 | 0 | 0 | 0 | 0 | 0 |
| Tni_Contig_4 |  | TE Transib | 13 | 0 | 0 | 0 | 0 | 0 | 6 | 0 | 1 | 0 | 0 |
| Tni_Contig_5 |  | TE Gypsy | 1 | 0 | 0 | 1 | 0 | 0 | 0 | 0 | 3 | 0 | 0 |
| Tni_Contig_6 |  | TE Harbinger | 29 | 2 | 11 | 1 | 8 | 5 | 9 | 2 | 0 | 0 | 0 |
| Tni_Contig_7 |  | undetermined | 4 | 0 | 0 | 0 | 0 | 0 | 2 | 2 | 0 | 1 | 1 |
| Tni_Contig_8* |  | TE Mariner | 26 | 0 | 0 | 11 | 1 | 1 | 5 | 0 | 0 | 0 | 0 |
| Tni_Contig_9 |  | TE Mariner | 65 | 0 | 3 | 4 | 3 | 2 | 6 | 0 | 3 | 0 | 1 |
| Tni_Contig_10 |  | TE Sola | 7 | 1 | 0 | 0 | 0 | 0 | 0 | 0 | 0 | 0 | 0 |
| Tni_Contig_11 | f | TE Harbinger | 2098 | 819 | 156 | 374 | 479 | 837 | 938 | 561 | 348 | 594 | 266 |
| Tni_Contig_12 |  | TE Piggybac | 147 | 1 | 0 | 4 | 0 | 0 | 0 | 2 | 8 | 3 | 0 |
| Tni_Contig_13* | f | TE Harbinger | 2270 | 1080 | 257 | 519 | 547 | 673 | 1143 | 639 | 310 | 786 | 307 |
| Tni_Contig_14 |  | TE Sola | 539 | 15 | 5 | 8 | 11 | 20 | 45 | 22 | 7 | 6 | 9 |
| Tni_Contig_15 |  | TE Copia | 136 | 7 | 3 | 9 | 3 | 12 | 92 | 3 | 3 | 0 | 5 |
| Tni_Contig_16 |  | TE Piggybac | 155 | 0 | 2 | 7 | 0 | 0 | 0 | 2 | 7 | 2 | 0 |
| Tni_Contig_17* |  | TE Mariner | 72 | 0 | 0 | 0 | 0 | 0 | 0 | 0 | 0 | 0 | 0 |

**Table S1 (continued)**

|  |  |  |  | ***Trichoplusia ni* G10** | | | | | | | | | |
| --- | --- | --- | --- | --- | --- | --- | --- | --- | --- | --- | --- | --- | --- |
| **Accession number**  **of host contig** | **Contig redundancy** | **Nature of contigs** | ***T. ni* G0** | **1** | **2** | **3** | **4** | **5** | **6** | **7** | **8** | **9** | **10** |
| Tni_Contig_18* |  | undetermined | 0 | 0 | 0 | 1 | 1 | 1 | 1 | 1 | 0 | 0 | 0 |
| Tni_Contig_19 |  | TE hAT | 5 | 0 | 0 | 1 | 0 | 0 | 1 | 3 | 0 | 0 | 0 |
| Tni_Contig_20 |  | TE Harbinger | 18 | 0 | 3 | 4 | 2 | 7 | 10 | 3 | 2 | 0 | 3 |
| Tni_Contig_21 |  | TE Mariner | 2227 | 0 | 0 | 0 | 0 | 0 | 0 | 0 | 0 | 0 | 0 |
| Tni_Contig_22 |  | TE Sola | 37 | 0 | 0 | 2 | 0 | 0 | 0 | 0 | 2 | 0 | 0 |
| Tni_Contig_23 |  | TE Transib | 99 | 0 | 6 | 1 | 1 | 0 | 0 | 0 | 0 | 0 | 1 |
| Tni_Contig_24 |  | TE P | 66 | 0 | 6 | 4 | 0 | 3 | 6 | 3 | 1 | 0 | 0 |
| Tni_Contig_25 |  | TE Transib | 66 | 0 | 4 | 4 | 4 | 1 | 4 | 1 | 1 | 1 | 1 |
| Tni_Contig_26 |  | undetermined | 3 | 0 | 0 | 0 | 0 | 0 | 1 | 1 | 0 | 0 | 3 |
| Tni_Contig_27 |  | TE Sola | 1334 | 2 | 3 | 1 | 0 | 0 | 0 | 2 | 0 | 0 | 12 |
| Tni_Contig_28* |  | TE Piggybac | 13 | 0 | 1 | 2 | 1 | 0 | 2 | 0 | 0 | 1 | 0 |
|  |  |  |  |  |  |  |  |  |  |  |  |  |  |
|  |  |  |  |  |  |  |  |  |  |  |  |  |  |
|  |  |  |  |  |  |  |  |  |  |  |  |  |  |
|  |  |  |  |  |  |  |  |  |  |  |  |  |  |
|  |  |  |  |  |  |  |  |  |  |  |  |  |  |
|  |  |  |  |  |  |  |  |  |  |  |  |  |  |
|  |  |  |  |  |  |  |  |  |  |  |  |  |  |
|  |  |  |  |  |  |  |  |  |  |  |  |  |  |
|  |  |  |  |  |  |  |  |  |  |  |  |  |  |
|  |  |  |  |  |  |  |  |  |  |  |  |  |  |
|  |  |  |  |  |  |  |  |  |  |  |  |  |  |
|  |  |  |  |  |  |  |  |  |  |  |  |  |  |

**Table S1 (continued)**

|  |  |  | ***Spodoptera exigua* G10** | | | | | | | | | |
| --- | --- | --- | --- | --- | --- | --- | --- | --- | --- | --- | --- | --- |
| **Accession number**  **of host contig** | **Contig redundancy** | **Nature of contigs** | **1** | **2** | **3** | **4** | **5** | **6** | **7** | **8** | **9** | **10** |
| SEUC10095_TC01 |  | TE Piggybac | 2 | 0 | 3 | 0 | 0 | 0 | 0 | 0 | 0 | 0 |
| SEUC19736_TC01 |  | undetermined | 0 | 3 | 3 | 0 | 0 | 0 | 0 | 0 | 0 | 0 |
| SEUC26615_TC01 |  | TE hAT | 1 | 8 | 1 | 0 | 0 | 2 | 0 | 1 | 0 | 1 |
| SEUC27063_TC12 |  | undetermined | 6 | 6 | 2 | 0 | 0 | 1 | 0 | 0 | 1 | 1 |
| SEUC30172_TC01* |  | TE Piggybac | 0 | 1 | 1 | 0 | 1 | 0 | 0 | 0 | 0 | 0 |
| SEUC30902_TC20* |  | TE Harbinger | 1 | 0 | 0 | 0 | 0 | 0 | 0 | 2 | 0 | 0 |
| SEUC32160_TC07 |  | TE Piggybac | 0 | 0 | 0 | 0 | 17 | 0 | 0 | 0 | 0 | 0 |
| SEUC33946_TC05* |  | TE Piggybac | 1 | 0 | 3 | 0 | 0 | 0 | 0 | 0 | 0 | 0 |
| SEUC36321_TC05* |  | TE hAT | 0 | 7 | 0 | 0 | 0 | 0 | 0 | 0 | 0 | 0 |
| SEUC37551_TC01 |  | TE MULE | 5 | 2 | 5 | 0 | 1 | 1 | 0 | 4 | 1 | 0 |
| SEUC37551_TC02 | b | TE MULE | 17 | 13 | 36 | 5 | 26 | 26 | 3 | 72 | 13 | 3 |
| SEUC37551_TC03 | b | TE MULE | 2 | 0 | 0 | 0 | 0 | 0 | 0 | 2 | 0 | 0 |
| SEUC38208_TC09* |  | TE Mariner | 13 | 16 | 18 | 4 | 5 | 1 | 1 | 3 | 2 | 4 |
| SEUC39640_TC01 |  | undetermined | 0 | 3 | 0 | 0 | 0 | 0 | 0 | 0 | 0 | 0 |
| SEUC40004_TC01 | c | TE Sola | 1 | 10 | 12 | 0 | 2 | 2 | 1 | 7 | 1 | 2 |
| SEUC40004_TC02 |  | undetermined | 1 | 1 | 1 | 0 | 0 | 0 | 0 | 0 | 0 | 0 |
| SEUC40004_TC03 | c | TE Sola | 17 | 17 | 14 | 0 | 2 | 3 | 1 | 4 | 4 | 5 |
| SEUC40004_TC05 |  | TE Sola | 8 | 17 | 18 | 1 | 7 | 7 | 4 | 16 | 9 | 12 |
| SEUC40340_TC04* | d | TE Harbinger | 7 | 9 | 5 | 0 | 0 | 0 | 0 | 8 | 0 | 0 |
| SEUC40340_TC07 | e | TE Harbinger | 8 | 18 | 6 | 0 | 0 | 0 | 0 | 13 | 0 | 6 |
| SEUC40340_TC08 | e | TE Harbinger | 21 | 17 | 17 | 0 | 0 | 0 | 0 | 13 | 0 | 8 |
| SEUC40340_TC09 | d | TE Harbinger | 18 | 27 | 15 | 0 | 0 | 0 | 0 | 25 | 0 | 4 |
| SEUC41948_TC13 |  | TE Gypsy | 0 | 18 | 0 | 0 | 0 | 0 | 0 | 0 | 0 | 0 |
| SEUC42258_TC01 |  | TE Pao | 1 | 0 | 1 | 0 | 2 | 1 | 0 | 3 | 1 | 0 |
| SEUC42445_TC05 |  | TE Helitron | 1 | 1 | 2 | 0 | 1 | 0 | 0 | 0 | 1 | 0 |
| SEUC42630_TC24* |  | TE Helitron | 1 | 1 | 1 | 0 | 0 | 0 | 0 | 0 | 0 | 0 |
| SEUC44746_TC22 |  | undetermined | 0 | 4 | 1 | 0 | 0 | 0 | 0 | 0 | 0 | 0 |
| SEUC46157_TC01 |  | TE Mariner | 0 | 2 | 0 | 0 | 1 | 0 | 0 | 0 | 0 | 0 |
| Spodo_Contig_1* |  | TE Harbinger | 2 | 1 | 1 | 1 | 2 | 2 | 2 | 0 | 7 | 2 |
| Spodo_Contig_2 |  | undetermined | 6 | 2 | 5 | 0 | 0 | 0 | 0 | 1 | 0 | 0 |
| Spodo_Contig_3* |  | TE Harbinger | 0 | 0 | 0 | 0 | 1 | 1 | 0 | 2 | 4 | 4 |
| Spodo_Contig_4 |  | TE Gypsy | 6 | 15 | 7 | 0 | 0 | 2 | 0 | 1 | 0 | 0 |
| Spodo_Contig_5 |  | TE Piggybac | 512 | 99 | 107 | 12 | 14 | 4 | 2 | 29 | 22 | 10 |
| Spodo_Contig_6* |  | TE Piggybac | 7 | 14 | 10 | 2 | 1 | 4 | 0 | 5 | 6 | 3 |
| Spodo_Contig_7 |  | TE Harbinger | 1 | 7 | 56 | 4 | 6 | 24 | 1 | 3 | 896 | 1 |
| Spodo_Contig_8* |  | TE Harbinger | 0 | 0 | 11 | 0 | 0 | 0 | 0 | 0 | 0 | 0 |
| Spodo_Contig_9 |  | TE Harbinger | 73 | 1 | 0 | 0 | 0 | 2 | 0 | 0 | 0 | 0 |
| Spodo_Contig_10 |  | TE Piggybac | 198 | 1131 | 220 | 1 | 8 | 8 | 2 | 9 | 25 | 52 |

**Table S1 (continued)**

|  |  |  | ***Spodoptera exigua* G10** | | | | | | | | | |
| --- | --- | --- | --- | --- | --- | --- | --- | --- | --- | --- | --- | --- |
| **Accession number**  **of host contig** | **Contig redundancy** | **Nature of contigs** | **1** | **2** | **3** | **4** | **5** | **6** | **7** | **8** | **9** | **10** |
| Spodo_Contig_11 |  | TE Piggybac | 15 | 15 | 13 | 0 | 1 | 2 | 0 | 1 | 1 | 1 |
| Spodo_Contig_12 |  | TE Copia | 21 | 8 | 9 | 0 | 1 | 2 | 0 | 0 | 1 | 1 |
| Spodo_Contig_13* |  | TE Harbinger | 4 | 50 | 16 | 2 | 19 | 3 | 2 | 18 | 20 | 37 |
| Spodo_Contig_14 |  | TE Gypsy | 25 | 7 | 7 | 0 | 1 | 8 | 2 | 4 | 5 | 4 |
| Spodo_Contig_15 |  | TE Harbinger | 9 | 19 | 10 | 0 | 5 | 10 | 3 | 5 | 10 | 12 |
| Spodo_Contig_16 |  | TE Piggybac | 9 | 17 | 11 | 2 | 1 | 6 | 0 | 7 | 6 | 3 |
| Spodo_Contig_17* |  | TE Harbinger | 13 | 1 | 0 | 0 | 0 | 0 | 0 | 0 | 0 | 0 |
| Spodo_Contig_18 |  | TE Gypsy | 18 | 28 | 83 | 0 | 0 | 4 | 2 | 18 | 9 | 25 |
| Spodo_Contig_19 |  | undetermined | 20 | 7 | 5 | 0 | 0 | 1 | 0 | 0 | 0 | 0 |
| Spodo_Contig_20 |  | TE Harbinger | 0 | 15 | 95 | 6 | 5 | 1 | 0 | 32 | 52 | 0 |
| Spodo_Contig_21 |  | TE Harbinger | 5 | 6 | 4 | 0 | 7 | 4 | 48 | 26 | 20 | 25 |
| Spodo_Contig_22 |  | TE Harbinger | 0 | 2 | 25 | 1 | 2 | 0 | 0 | 11 | 10 | 1 |
| Spodo_Contig_23* |  | TE Piggybac | 7 | 8 | 46 | 0 | 5 | 0 | 0 | 10 | 0 | 1 |
| Spodo_Contig_24 |  | undetermined | 22 | 30 | 10 | 0 | 3 | 4 | 0 | 2 | 3 | 3 |
