# Supplementary material for: Continuous Influx of Genetic Material from Host to Virus Populations
Source: PLoS Genet. 2016 Feb 1;12(2):e1005838. doi: 10.1371/journal.pgen.1005838 (PMC4735498; doi:10.1371/journal.pgen.1005838)
Supplement: S2 Table — The upper half considers only independent transpositions; the lower half considers all transpositions, including viral replicates. An “x” in a cell indicates that a term is used in the model. The models retained correspond to the rows shown in bold. (DOCX) [file pgen.1005838.s002.docx]

**Table S2. Selection of models explaining the number of junctions by transposition of *S. exigua* DNA in 1500-bp genomic windows along the AcMNPV genome.** The upper half considers only independent transpositions; the lower half considers all transpositions, including viral replicates. An “x” in a cell indicates that a term is used in the model. The models retained correspond to the rows shown in bold.

| Sequencing depth | Number of targets | Number of transpositions of *T. ni* DNA | d.f. | log(likelihood) | AICc | Weight |
| --- | --- | --- | --- | --- | --- | --- |
|  |  | **x** | **2** | **-320.488** | **645.1** | **0.383** |
| x | x | x | 4 | -318.708 | 645.9 | 0.26 |
| x |  | x | 3 | -320.108 | 646.5 | 0.192 |
|  | x | x | 3 | -320.256 | 646.8 | 0.165 |
| x | x |  | 3 | -415.701 | 837.7 | 0 |
|  | x |  | 2 | -428.996 | 862.1 | 0 |
|  |  |  | 1 | -449.99 | 902 | 0 |
| x |  |  | 2 | -449.989 | 904.1 | 0 |
|  |  |  |  |  |  |  |
| **x** | **x** | **x** | **4** | **-4789.728** | **9587.9** | **0.98** |
| x |  | x | 3 | -4795.026 | 9596.3 | 0.015 |
| x | x |  | 3 | -4795.98 | 9598.2 | 0.006 |
|  | x | x | 3 | -4806.516 | 9619.3 | 0 |
| x |  |  | 2 | -4807.636 | 9619.4 | 0 |
|  | x |  | 2 | -4809.98 | 9624.1 | 0 |
|  |  | x | 2 | -4842.321 | 9688.8 | 0 |
|  |  |  | 1 | -4858.364 | 9718.8 | 0 |
