# Supplementary material for: Continuous Influx of Genetic Material from Host to Virus Populations
Source: PLoS Genet. 2016 Feb 1;12(2):e1005838. doi: 10.1371/journal.pgen.1005838 (PMC4735498; doi:10.1371/journal.pgen.1005838)
Supplement: S3 Table — The upper half considers only independent transpositions; the lower half considers all transpositions, including viral replicates. (DOCX) [file pgen.1005838.s003.docx]

**Table S3. Analyses of deviance of models explaining the number of junctions by transposition of *S. exigua* DNA in 1500-bp genomic windows along the AcMNPV genome.** The upper half considers only independent transpositions; the lower half considers all transpositions, including viral replicates.

| Terms | d.f. | Deviance | Percentage explained | *p*(>Chi) |
| --- | --- | --- | --- | --- |
| Null model | 89 | 478.82 | - | - |
| Number of transpositions of *T. ni* DNA | 1 | 259 | 54% | < 2.2 × 10^-16^ |
|  |  |  |  |  |
| Null model | 89 | 9272 | - | - |
| Sequencing depth | 1 | 101.45 | 1.1% | < 2.2 × 10^-16^ |
| Number of targets | 1 | 23.3 | 0.2% | 1.38 × 10^-6^ |
| Number of transpositions of *T. ni* DNA | 1 | 12.5 | 0.1% | 4.06 × 10^-4^ |
