# Supplementary material for: Continuous Influx of Genetic Material from Host to Virus Populations
Source: PLoS Genet. 2016 Feb 1;12(2):e1005838. doi: 10.1371/journal.pgen.1005838 (PMC4735498; doi:10.1371/journal.pgen.1005838)
Supplement: S4 Table — TE similarities between S. exigua and the other insect species are given in percent nucleotide identity. Numbers in brackets next to percent nucleotide identities are the numbers of copies (or fragments of copies) longer than 100 bp found by Blastn in each insect genome and the length of the longest copy or fragment of copy in base pairs. TE numbers correspond to those in Fig 4A. Divergence times were taken from refs [44–46]. (DOCX) [file pgen.1005838.s004.docx]

**Table S4. Gene and transposable element (TE) similarities between *Spodoptera exigua* and other insect species.** TE similarities between *S. exigua* and the other insect species are given in percent nucleotide identity. Numbers in brackets next to percent nucleotide identities are the number of copies or fragments of copies longer than 100 bp found by blastn in each insect genome and the length of the longest copy or fragment of copy in base pairs. TE numbers correspond to those in Fig. 3A. Divergence times were taken from Wiegmann et al. (2011); Wahlberg et al. (2013); Misof et al. (2014).

|  | |  | |  | ***Plutella xylostella* (Plutelidae)** | ***Melitaea cinxia* (Nymphalidae)** | ***Chilo suppressalis* (Crambidae)** | ***Glossina fusciceps* (Diptera)** | ***Bombyx mori* (Bombycidae)** | ***Manduca sexta* (Sphingidae)** | ***Danaus plexippus* (Nymphalidae)** | ***Plodia interpunctella* (Pyralidae)** | ***Papilio glaucus* (Papilionidae)** | ***Limnephilus lunatus* (Trichoptera)** | ***Heliconius melpomene* (Nymphalidae)** | ***Athalia rosae* (Hymenoptera)** | ***Bactrocera dorsalis* (Diptera)** |
| --- | --- | --- | --- | --- | --- | --- | --- | --- | --- | --- | --- | --- | --- | --- | --- | --- | --- |
| **Gene similarity between *S. exigua* and other species (1 - gene synonymous distance) x 100** | | | **Actin** | | 74 | 80 | 83 | 30 | 79 | 80 | 82 | 80 | 76 | 68 | 79 | 62 | 51 |
|  |  |  | **AMP** | | 57 | 43 | 55 | NA | 45 | 49 | 48 | 43 | 53 | 43 | 23 | 4 | 26 |
|  |  |  | **ecdy** | | 26 | 25 | 34 | 2 | 21 | 28 | 33 | 32 | 35 | 26 | 21 | 11 | 16 |
|  |  |  | **elong** | | 60 | 63 | 59 | 26 | 59 | 68 | 71 | 68 | 58 | 49 | 57 | 40 | 40 |
|  |  |  | **enol** | | 33 | 35 | 38 | 3 | 26 | 42 | 32 | 25 | 27 | 30 | 34 | 26 | 34 |
|  |  |  | **Gprot** | | 39 | 48 | 50 | 4 | 30 | 47 | 54 | 49 | 38 | 28 | 49 | 15 | 14 |
|  |  |  | **HSP** | | 52 | 27 | 57 | 12 | 46 | 49 | 44 | 36 | 48 | 44 | 21 | 43 | 6 |
|  |  |  | **nucl** | | 46 | 24 | 37 | 17 | 14 | 34 | 23 | 35 | 27 | 20 | 28 | 16 | 10 |
|  |  |  | **trans** | | 32 | 31 | 48 | 9 | 38 | 40 | 40 | 37 | 36 | 28 | 39 | 17 | 16 |
|  |  |  | **USP** | | 30 | 23 | 25 | 18 | 31 | 34 | 38 | 26 | 46 | 22 | 33 | NA | 10 |
|  |  |  | **WING** | | 27 | 19 | 26 | NA | 30 | 35 | 32 | 40 | 31 | 33 | 35 | 12 | 12 |
| **Average gene similarity (%)** | | | | | 43.3 | 38.0 | 46.5 | 13.4 | 38.2 | 46.9 | 46.3 | 43.1 | 43.4 | 35.0 | 40.1 | 28.8 | 21.9 |
| **Div. Time (Myrs)** | | | | | 140 | 117 | 110 | 360 | 88 | 94 | 117 | 110 | 117 | 230 | 117 | 325 | 290 |
| **contig name** | **TE length (bp)** | | **TE type** | |  | | | | | | | | | | | | |
| **SEUC30902_TC20** | 2219 | | Harbinger | | - | - | - | - | 87 (6; 148) | - | - | - | - | - | - | - | - |
| **Spodo_Contig_8** | 398 | | Harbinger | | - | - | 91 (2; 309) | - | - | - | - | - | - | - | - | - | - |
| **SEUC40340_TC04** | 961 | | Harbinger | | - | - | - | - | 91 (5; 252) | - | - | - | - | - | - | - | - |
| **Spodo_Contig_1** | 397 | | Harbinger | | 86 (1; 400) | - | - | - | 88 (2; 354) | - | 94 (1; 211) | - | - | - | - | - | - |
| **Spodo_Contig_13** | 807 | | Harbinger | | 93.5 (3; 184) | - | 93 (2; 315) | - | - | - | - | - | - | 85 (5; 575) | - | - | - |
| **SEUC36321_TC05** | 2389 | | hAT | | - | 100 (1; 1534) | 88 (3; 541) | - | 89.5 (3; 794) | - | 86 (4; 548) | - | - | 90 (6; 735) | - | - | - |
| **SEUC42630_TC24** | 736 | | helitron | | - | - | - | - | - | - | - | - | - | - | 88 (1; 102) | - | - |
| **SEUC38208_TC09** | 819 | | Mariner-Tc1 | | - | - | - | - | - | 88 (7; 273) | - | - | - | - | - | - | - |
| **Spodo_Contig_23** | 1990 | | Piggybac | | - | - | - | 86 (3; 769) | - | - | 87 (1; 1072) | - | - | - | - | - | - |
| **SEUC30172_TC01** | 2077 | | Piggybac | | 89 (10; 1828) | 87 (3; 2079) | 86 (1; 682) | - | - | - | - | - | - | - | - | - | - |
| **SEUC33946_TC05** | 2535 | | Piggybac | |  | 91 (2; 2135) | 88 (1; 447) | - | 91 (3; 1516) | 92 (1; 681) | 90.5 (2; 1257) | 91 (1; 543) | 90 (4; 943) | - | 91 (4; 2128) | 91 (2; 1595) | 90 (3; 414) |
| **Spodo_Contig_3** | 435 | | TE Harbinger | | - | - | - | - | 85 (>100; 435) | - | - | - | - | - | - | - | - |
| **Spodo_Contig_6** | 1071 | | TE Piggybac | | - | - | 87 (4; 650) | - | - | - | - | - | - | - | - | - | - |
| **Spodo_Contig_17** | 750 | | TE Harbinger | | - | - | 98 (4; 749) | - | - | - | - | - | - | - | - | - | - |

# References

Misof B Liu S Meusemann K Peters RS Donath A Mayer C Frandsen PB Ware J Flouri T Beutel RG et al. 2014. Phylogenomics resolves the timing and pattern of insect evolution. *Science* **346**(6210): 763-767.

Wahlberg N, Wheat CW, Pena C. 2013. Timing and Patterns in the Taxonomic Diversification of Lepidoptera (Butterflies and Moths). *Plos One* **8**(11).

Wiegmann BM, Trautwein MD, Winkler IS, Barr NB, Kim J-W, Lambkin C, Bertone MA, Cassel BK, Bayless KM, Heimberg AM et al. 2011. Episodic radiations in the fly tree of life. *Proc Natl Acad Sci USA* **108**(14): 5690-5695.
