# Supplementary material for: Continuous Influx of Genetic Material from Host to Virus Populations
Source: PLoS Genet. 2016 Feb 1;12(2):e1005838. doi: 10.1371/journal.pgen.1005838 (PMC4735498; doi:10.1371/journal.pgen.1005838)
Supplement: S1 Fig — (PDF) [file pgen.1005838.s006.pdf]

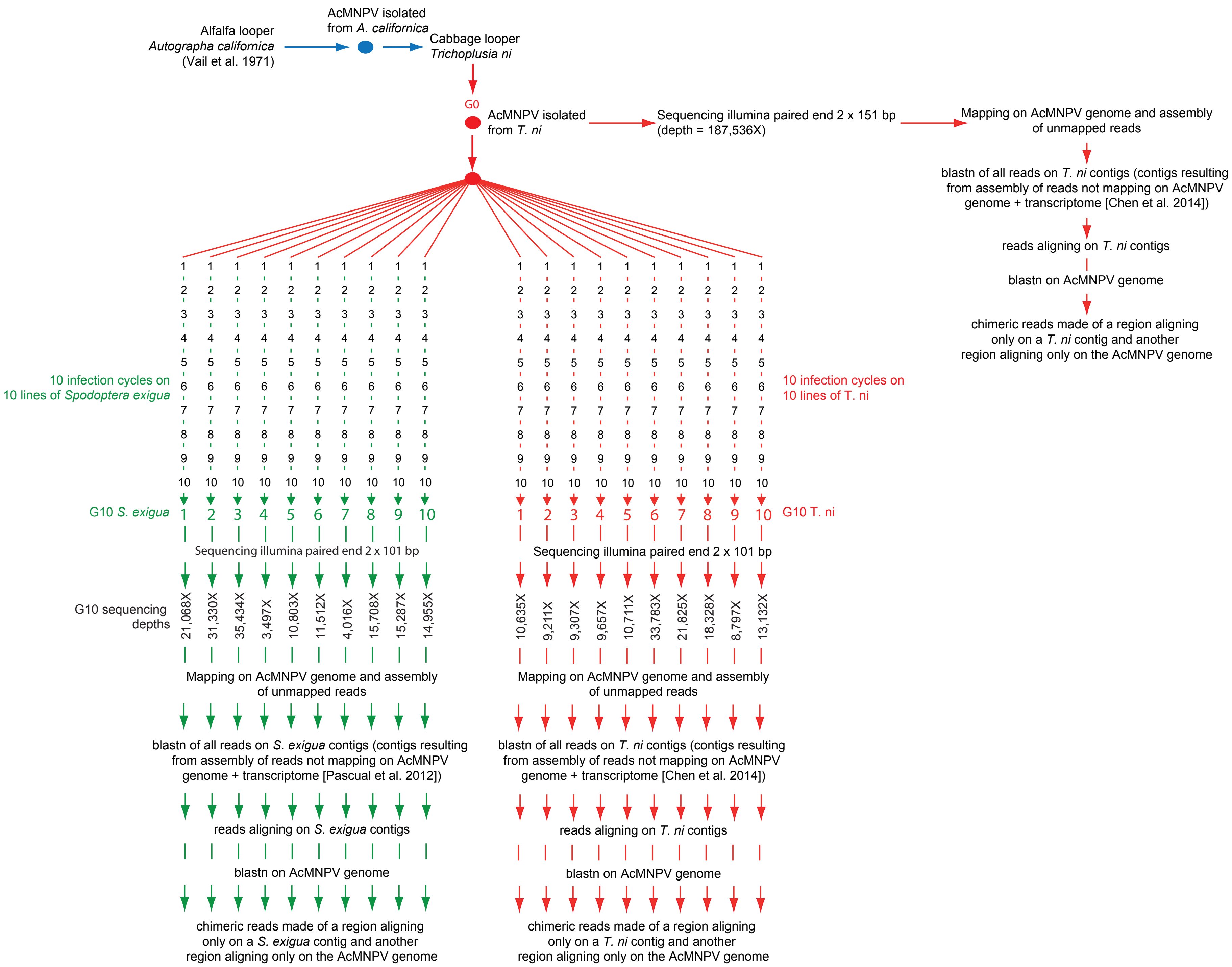

Fig. S1. Overview of the experimental evolution setup, sequencing and homology-based searches carried out in order to identify moth sequences integrated into populations of the AcMNPV baculovirus genome.
