# Supplementary material for: Continuous Influx of Genetic Material from Host to Virus Populations
Source: PLoS Genet. 2016 Feb 1;12(2):e1005838. doi: 10.1371/journal.pgen.1005838 (PMC4735498; doi:10.1371/journal.pgen.1005838)
Supplement: S2 Fig — While most of the junctions (5,637) are covered by one read, 1,412 are covered by two to 1,256 reads. (PDF) [file pgen.1005838.s007.pdf]

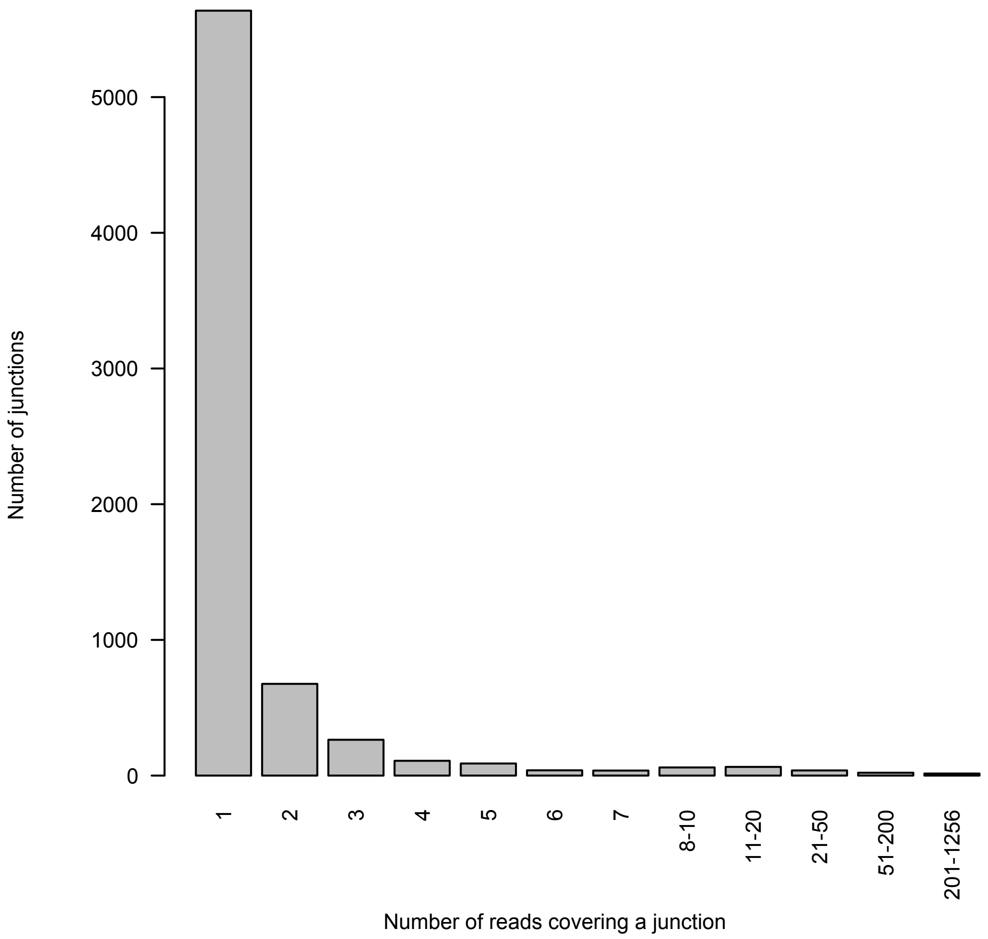

Fig. S2. Distribution of the number of reads covering a given junction.
While most of the junctions (5,637) are covered by one read, 1412 are covered by more two to 1,256 reads.
