# Supplementary material for: Continuous Influx of Genetic Material from Host to Virus Populations
Source: PLoS Genet. 2016 Feb 1;12(2):e1005838. doi: 10.1371/journal.pgen.1005838 (PMC4735498; doi:10.1371/journal.pgen.1005838)
Supplement: S3 Fig — Grey curves represent sequencing depth by reads composed of host sequences only. Red curves represent chimeric reads whose right parts are composed of host sequences (the left part being viral sequences) and green curves represent reads whose left parts are composed of host sequences. Black arrows indicate the locations and orientations of putative transposase (and Myb-like) genes along TEs. Note that for two contigs (Spodo_Contig_4 and 14) corresponding to Gypsy retrotransposons, chimeric reads fall within the contig sequence rather than on each extremity. This is likely due to missassembly of the retrotransposons, the contigs including only one long terminal repeat within the sequence rather than one on each extremity. (PDF) [file pgen.1005838.s008.pdf]

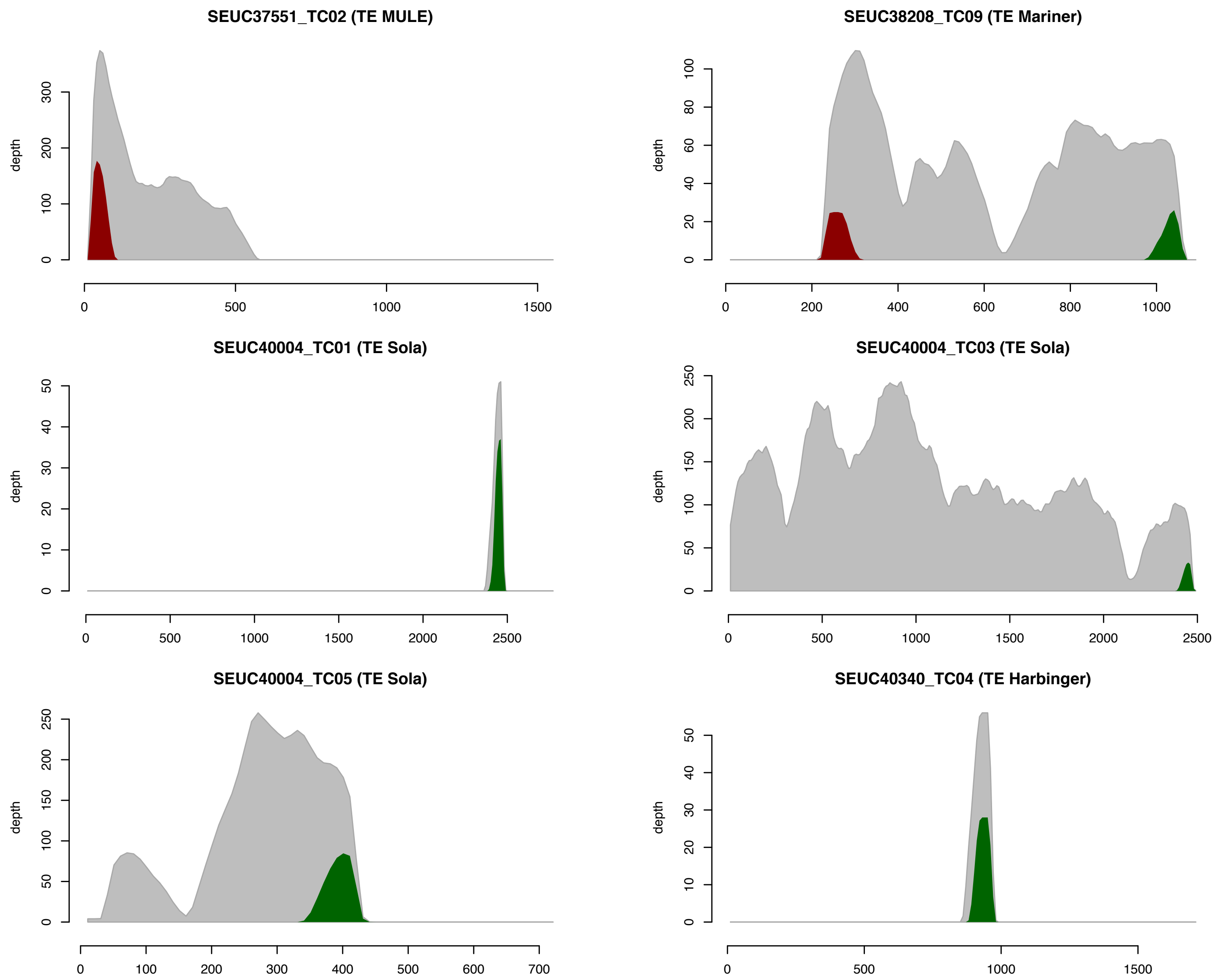

Fig. S3. Sequencing depth (number of reads covering a position) along all moth sequences found inserted into genomes of AcMNPV populations. Grey curves represent sequencing depth by reads composed of host sequence only. Red curves represent chimeric reads whose right parts are composed of host sequences (the left part being viral sequences) and green curves represent reads whose left parts are composed of host sequences. Black arrows indicate the locations and orientations of putative transposase (and Myb-like) genes along TEs. Note that for two contigs (Spodo_Contig_4 and 14) corresponding to Gypsy retrotransposons, chimeric reads fall within the contig sequence rather than on each extremity. This is likely due to missassembly of the retrotransposons, the contigs including only one long terminal repeat within the sequence rather than one on each extremity.

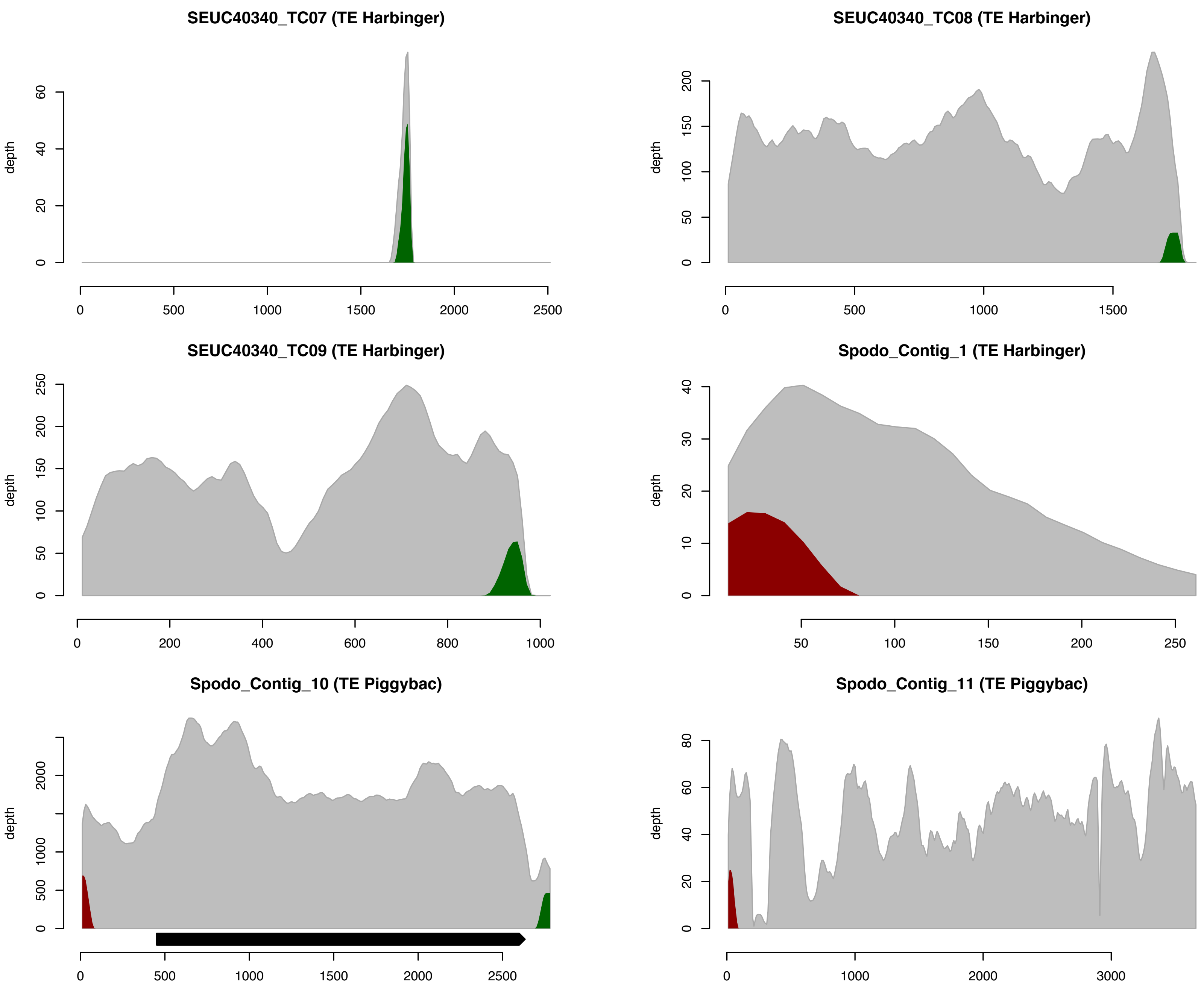

Fig. S3 (continued)

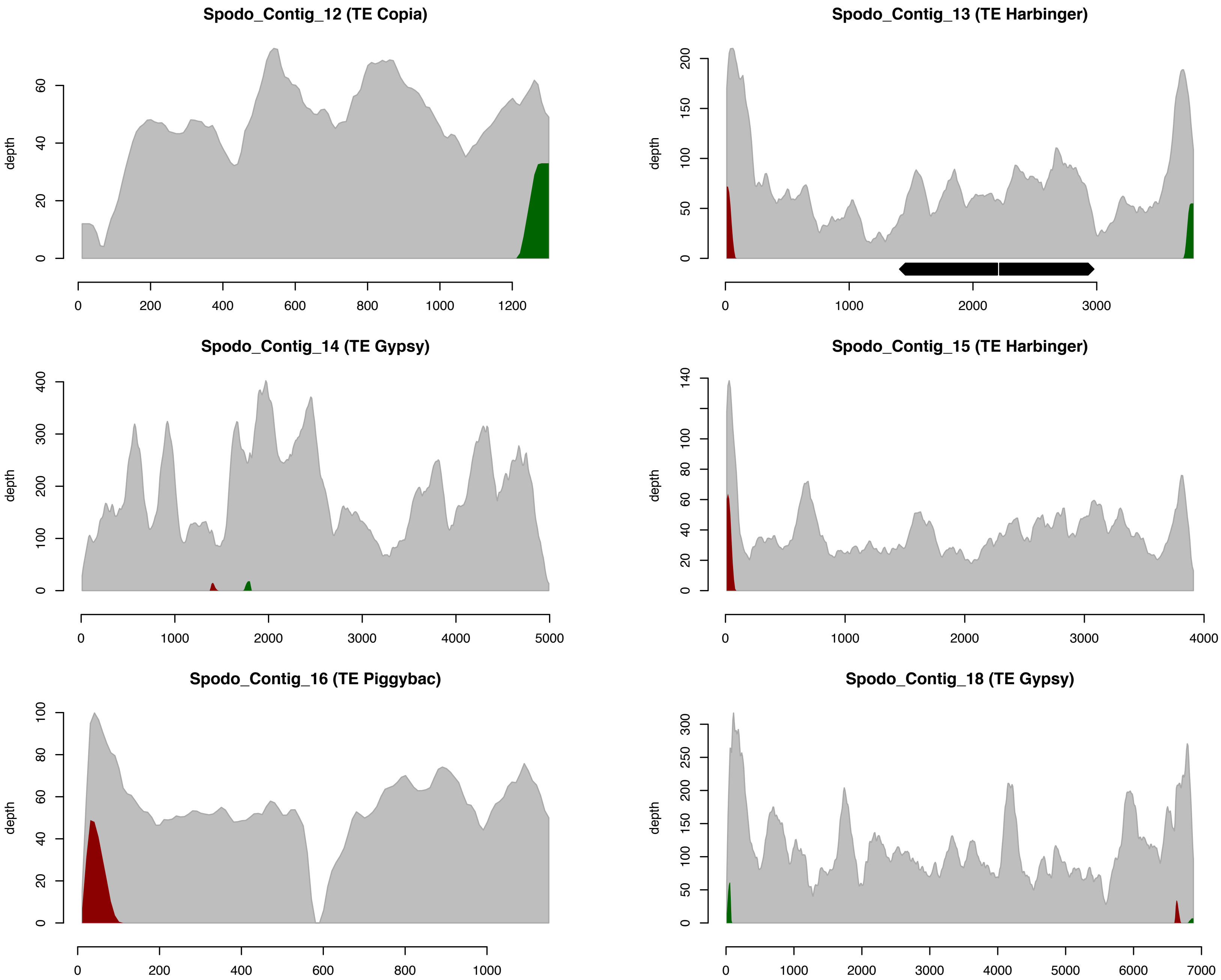

Fig. S3 (continued)

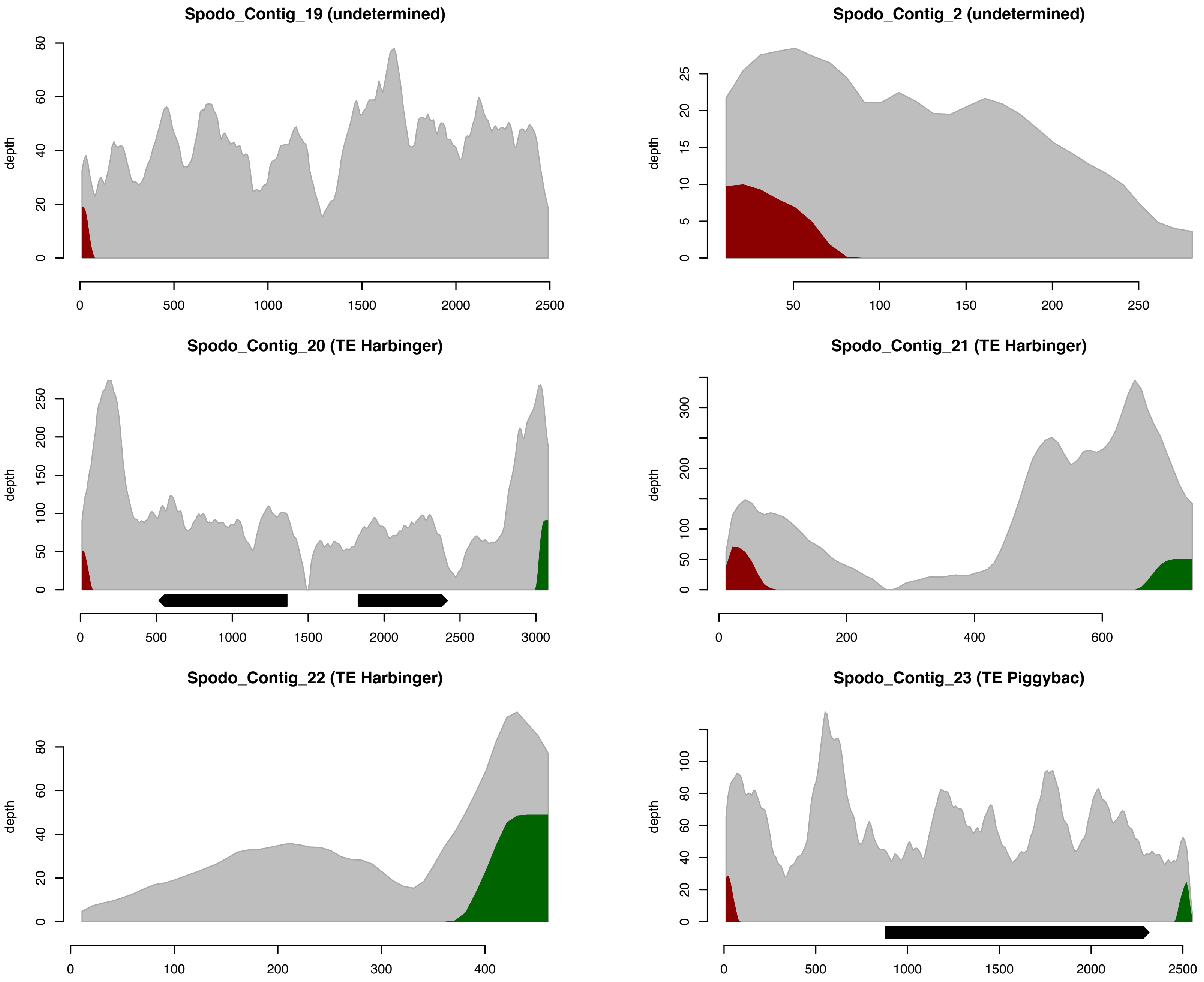

Fig. S3 (continued)

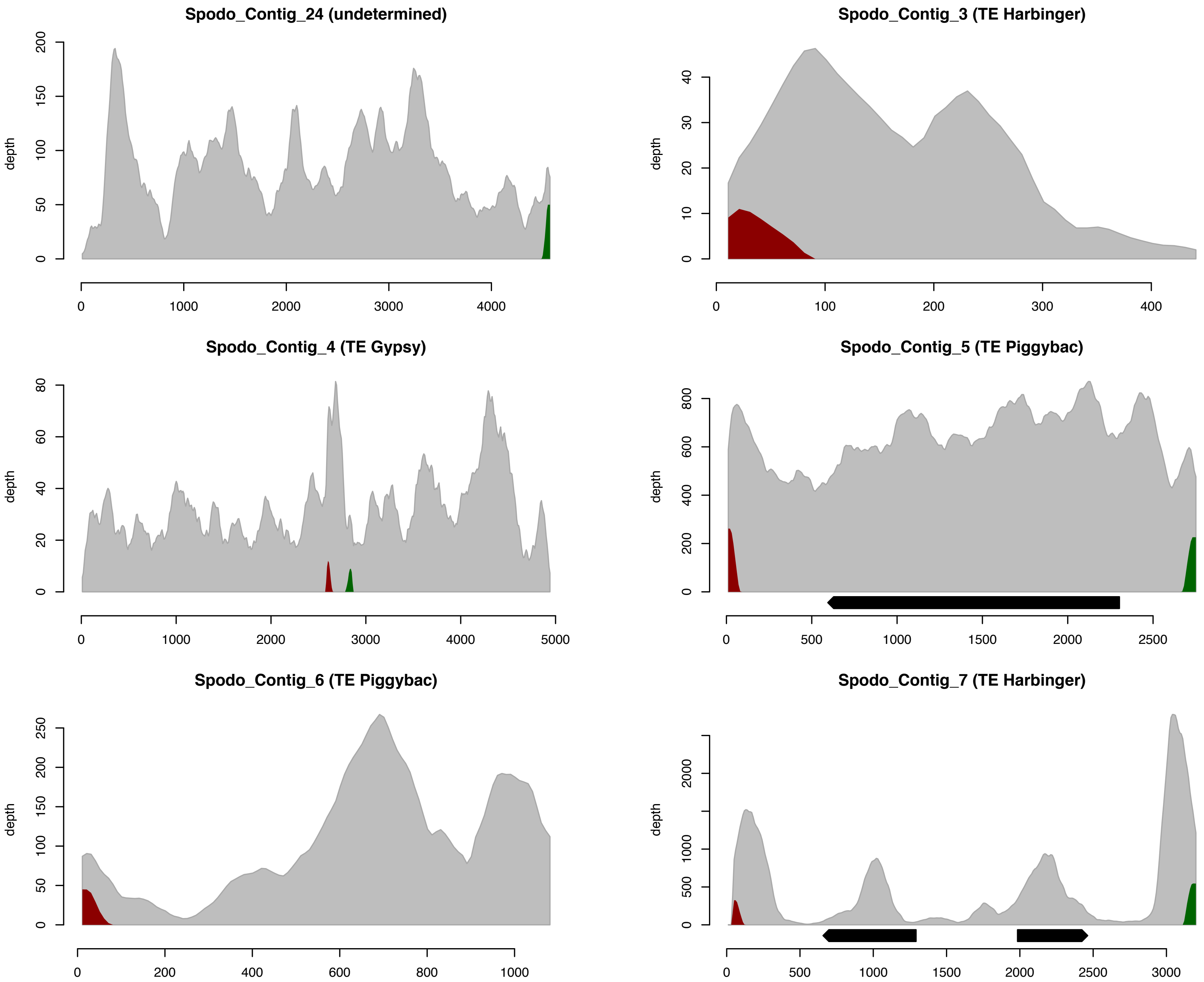

Fig. S3 (continued)

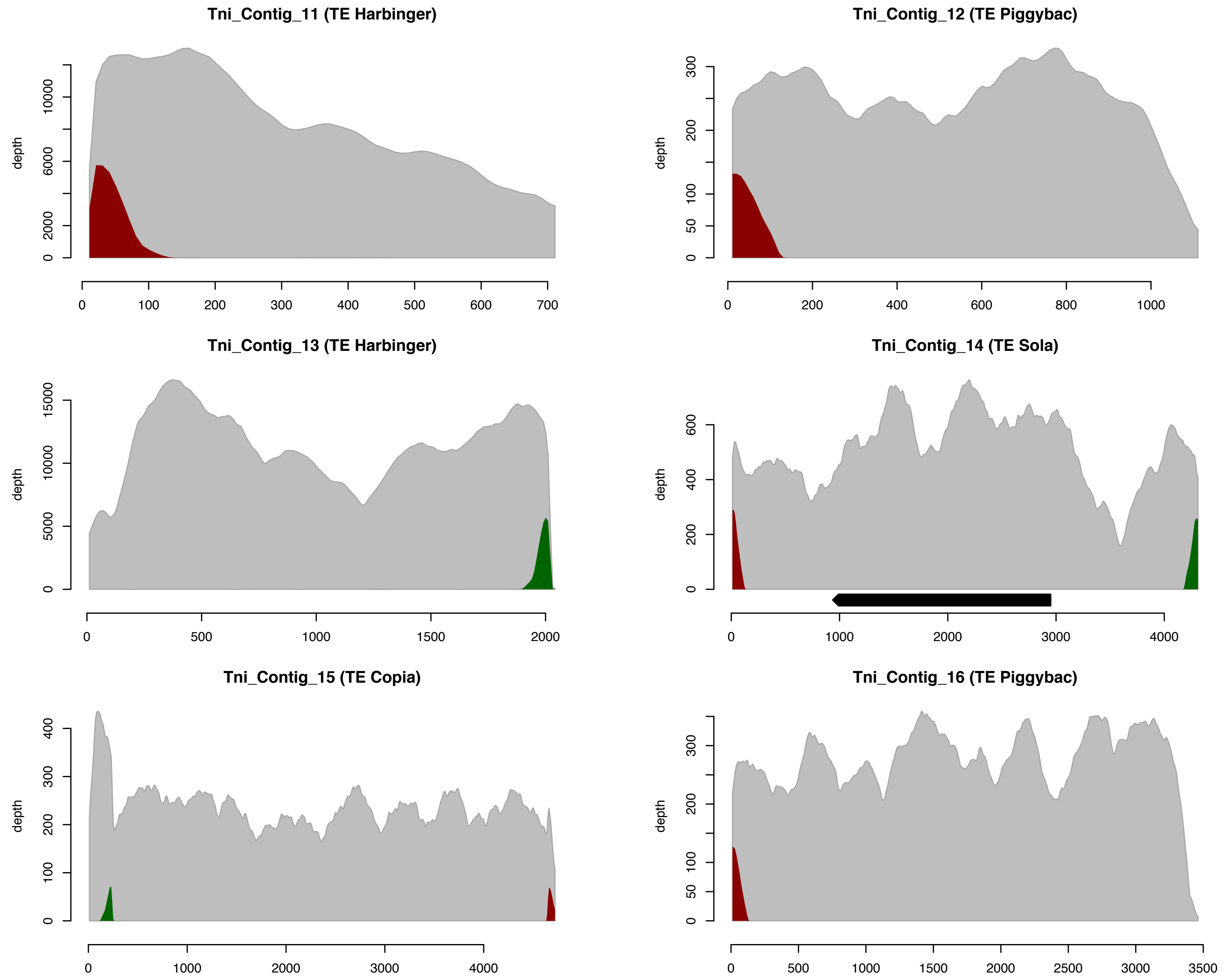

Fig. S3 (continued)

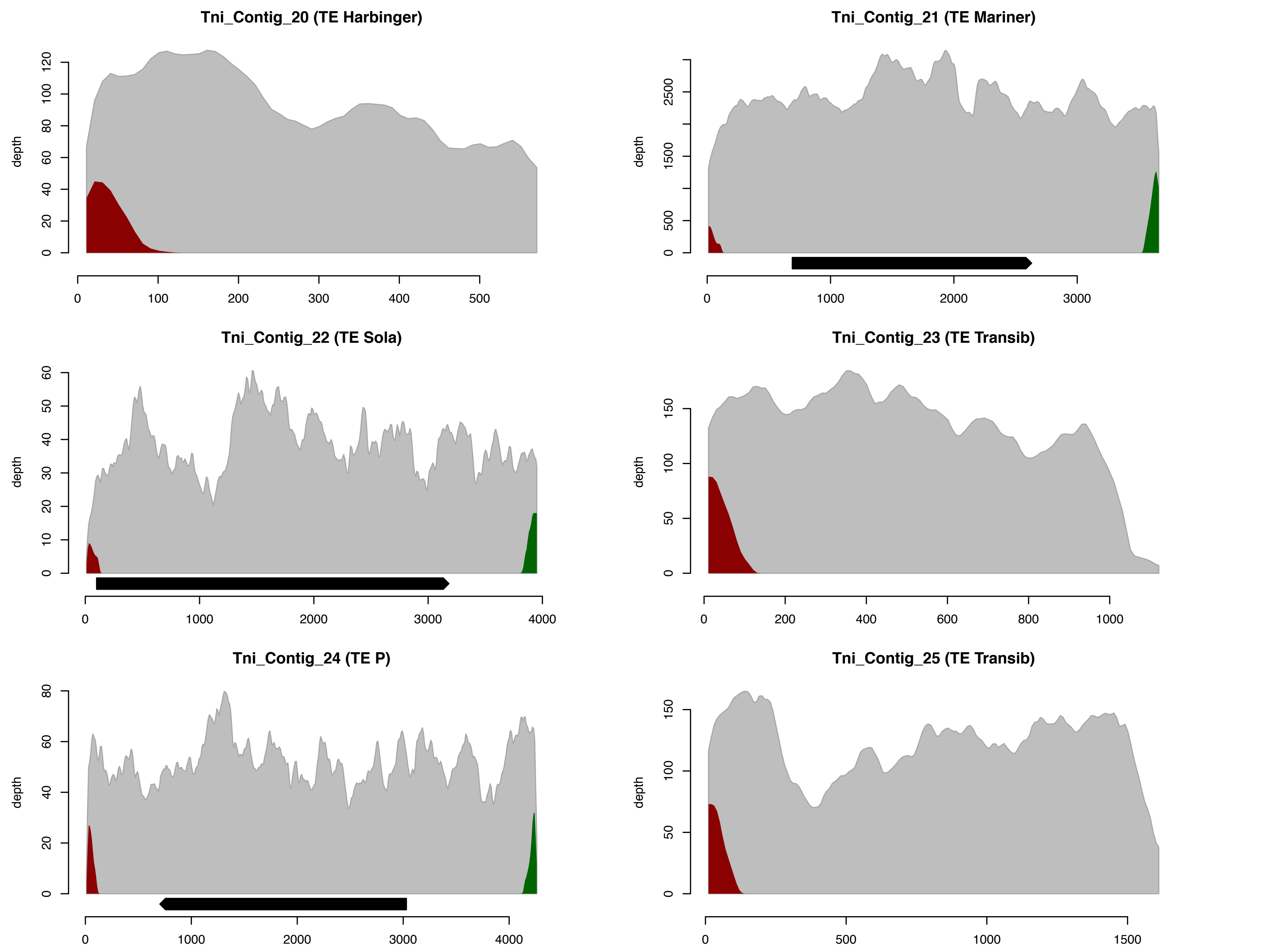

Fig. S3 (continued)

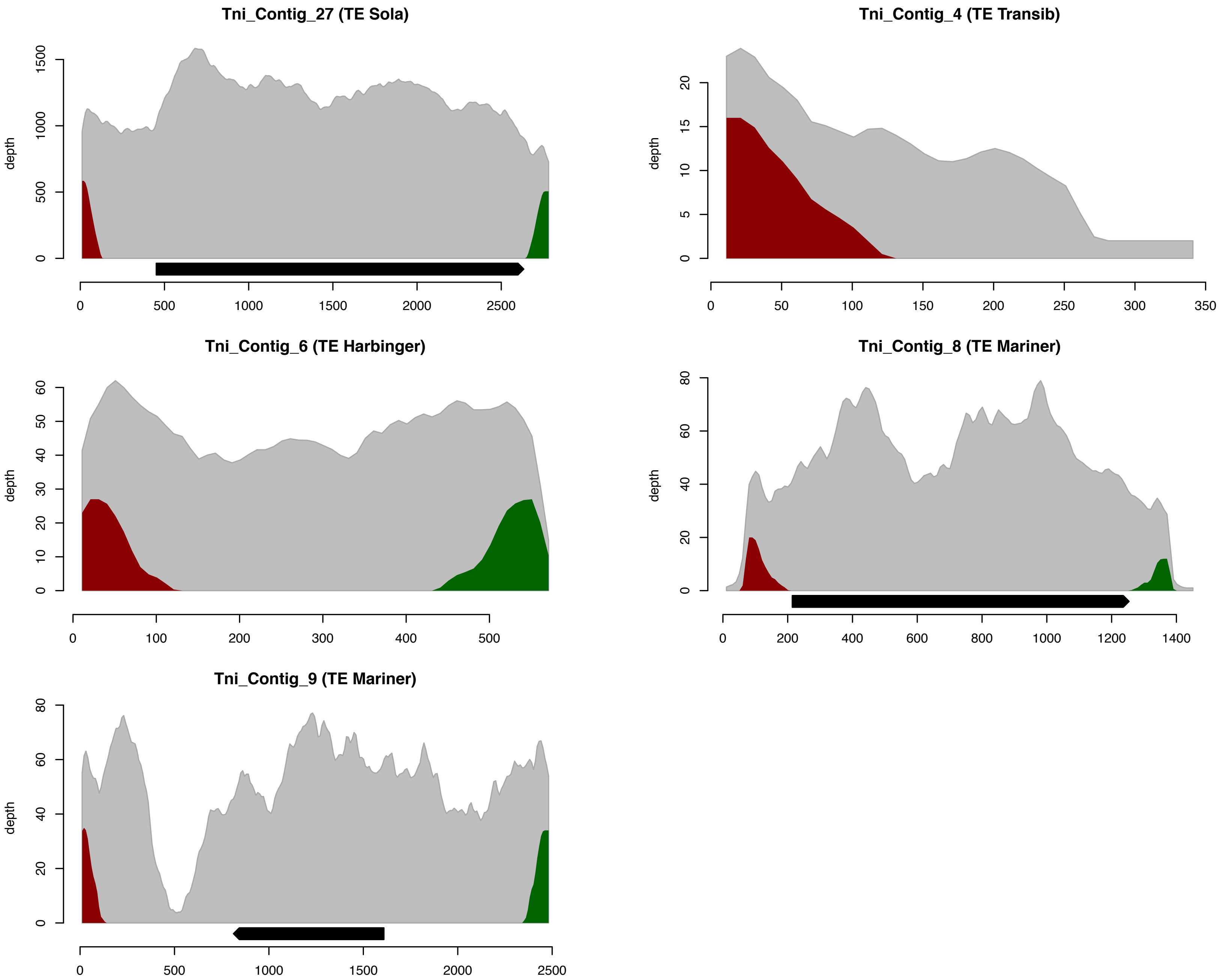

Fig. S3 (continued)
