# Supplementary material for: Continuous Influx of Genetic Material from Host to Virus Populations
Source: PLoS Genet. 2016 Feb 1;12(2):e1005838. doi: 10.1371/journal.pgen.1005838 (PMC4735498; doi:10.1371/journal.pgen.1005838)
Supplement: S4 Fig — For each logo we indicate the name of the contig (starting with “SEUC” for Spodoptera exigua transcripts and “GBKU” for Trichoplusia ni transcripts), its nature, the position within each contig of a given end of moth sequence found integrated in the AcMNPV genome as well as the number of junctions involving this given end, i.e., the number of different integration sites of the sequence along the AcMNPV genome. (PDF) [file pgen.1005838.s009.pdf]

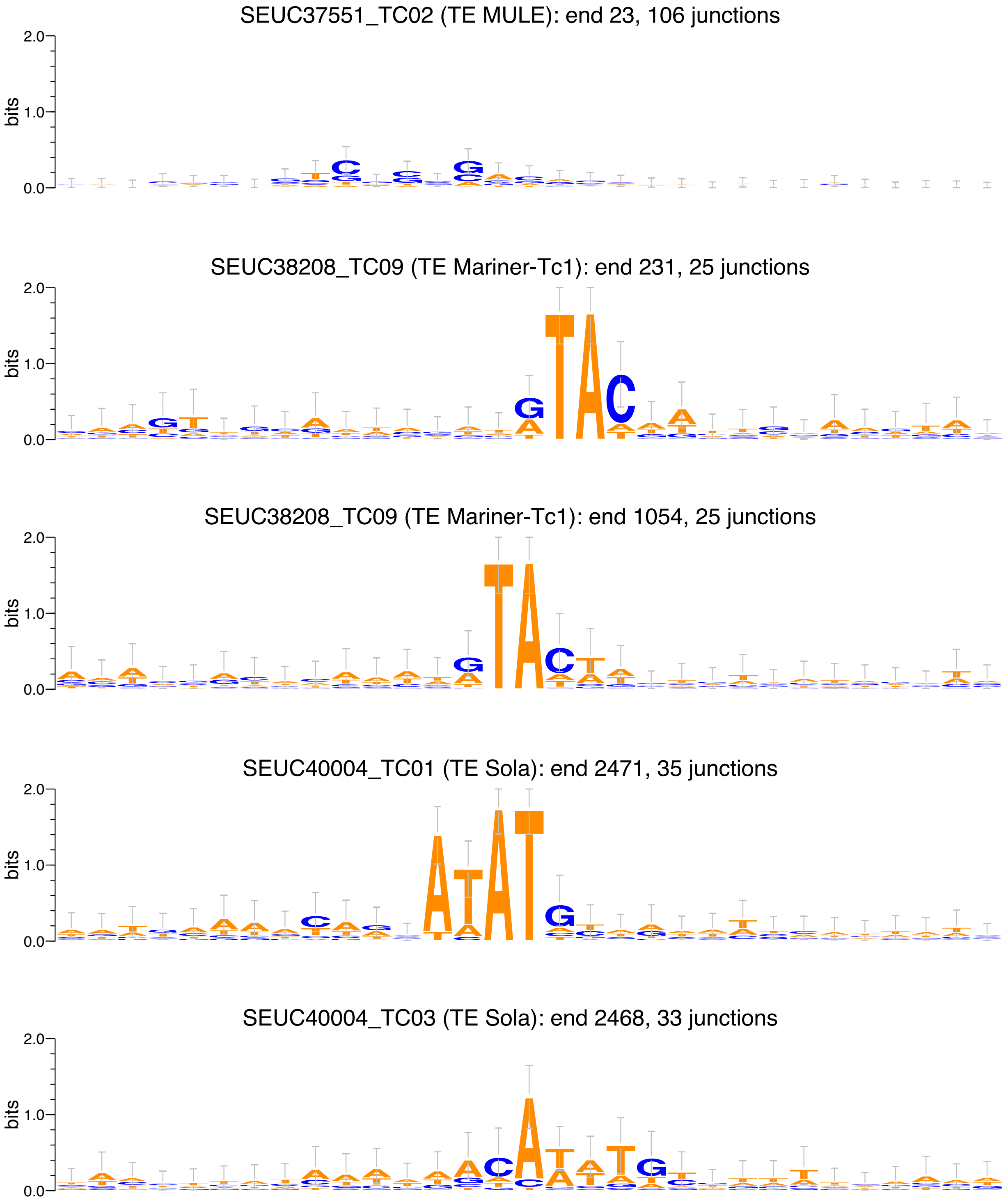

Fig. S4. Sequence conservation patterns (logos) at insertion sites of moth sequences in the AcMNPV baculovirus genome. For each logo we indicate the name of the contig (starting with “SEUC” for *Spodoptera exigua* and “GBKU” for *Trichoplusia ni*), the position within each contig of a given end of moth sequence found integrated in the AcMNPV genome as well as the number of junctions involving this given end, i.e., the number of different integration sites of the sequence along the AcMNPV genome.

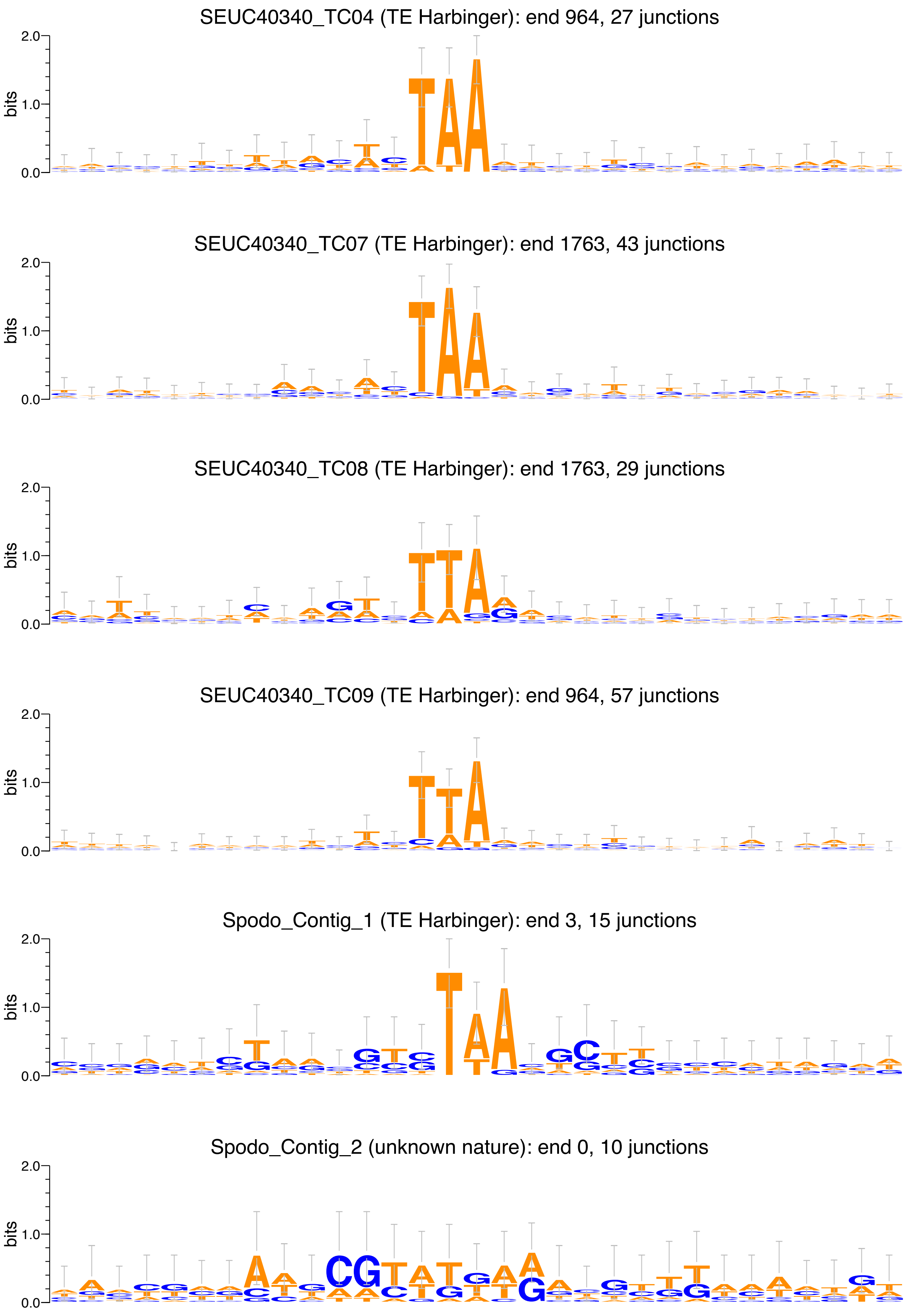

Fig. S4 (continued)

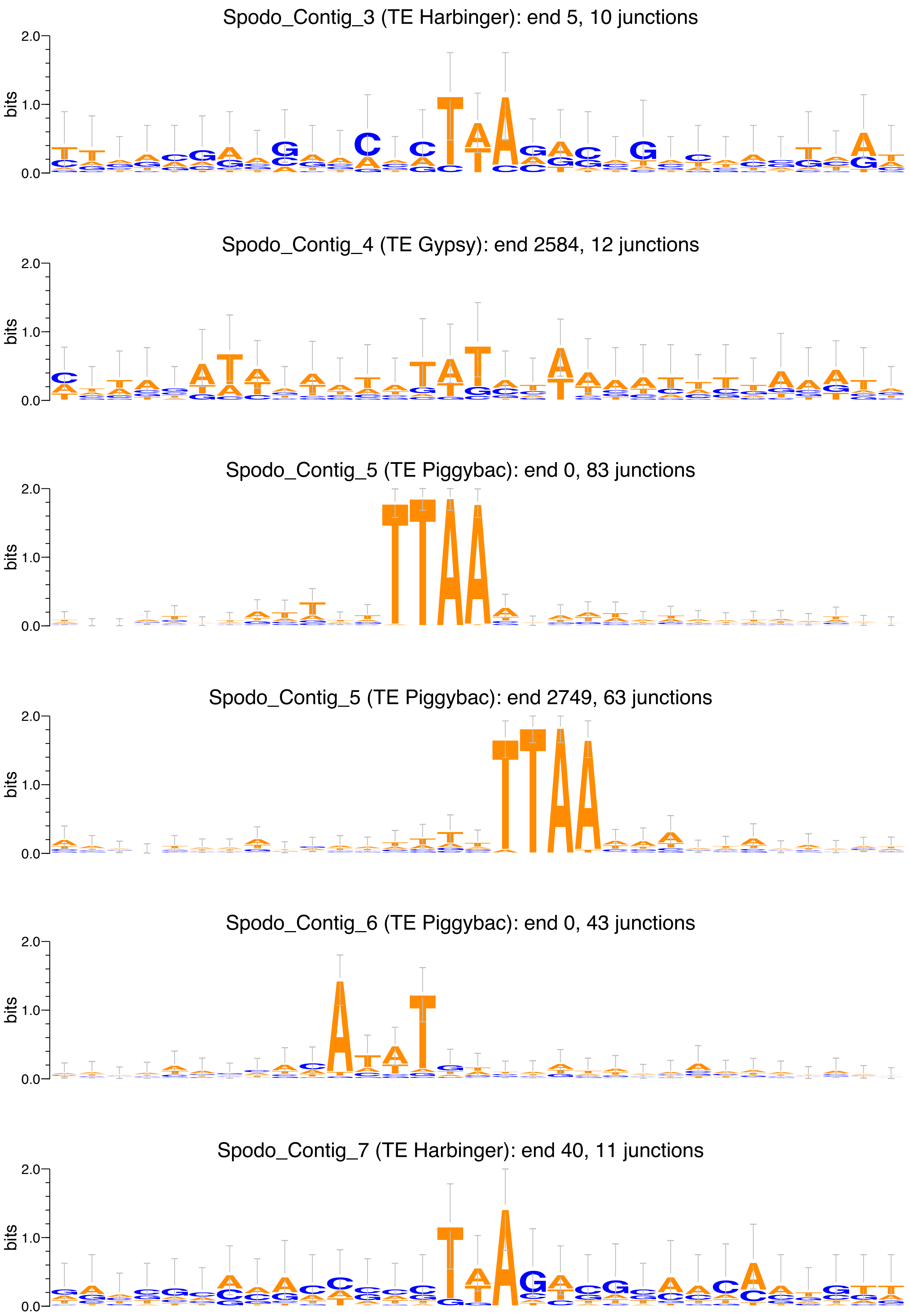

Fig. S4 (continued)

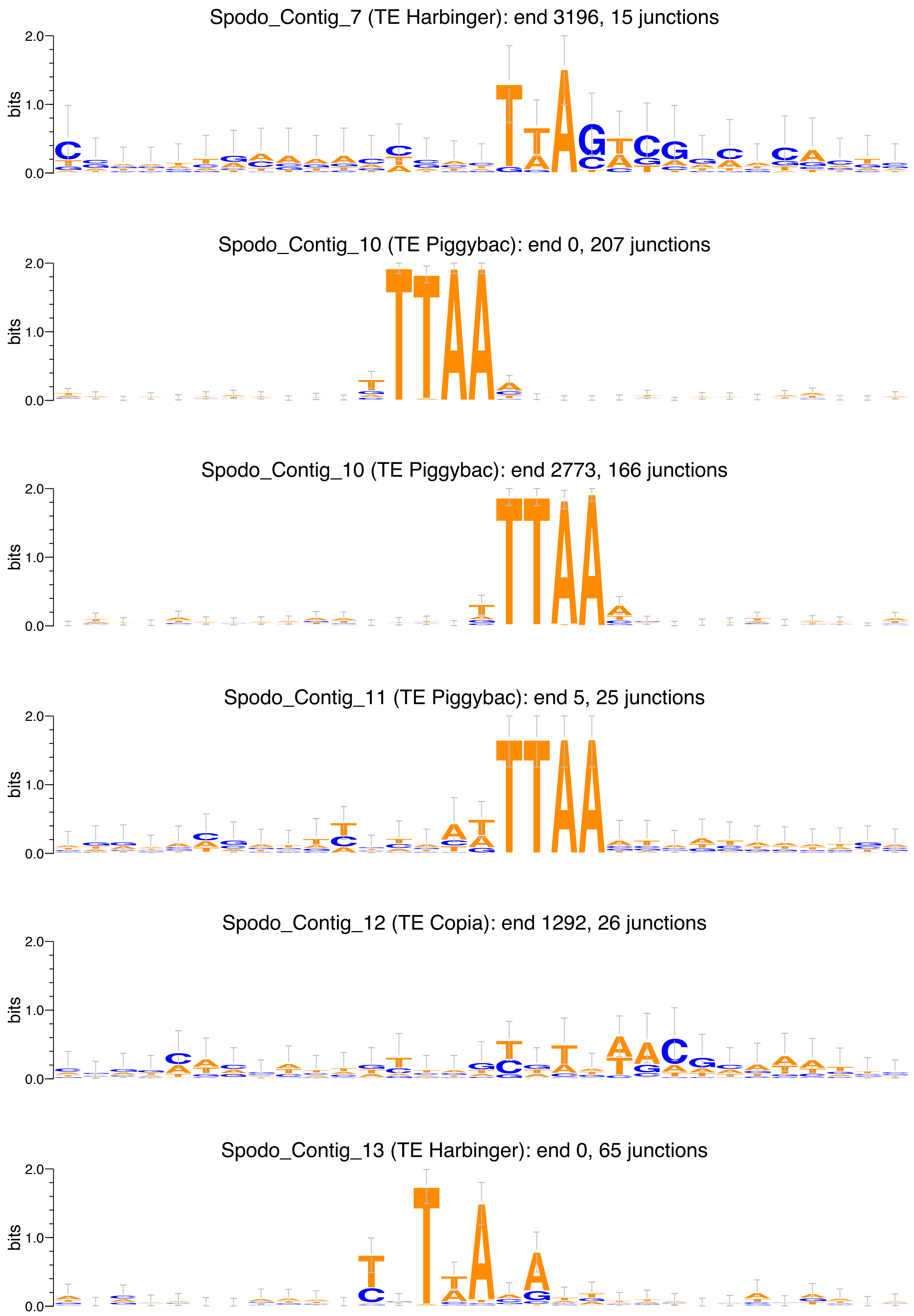

Fig. S4 (continued)

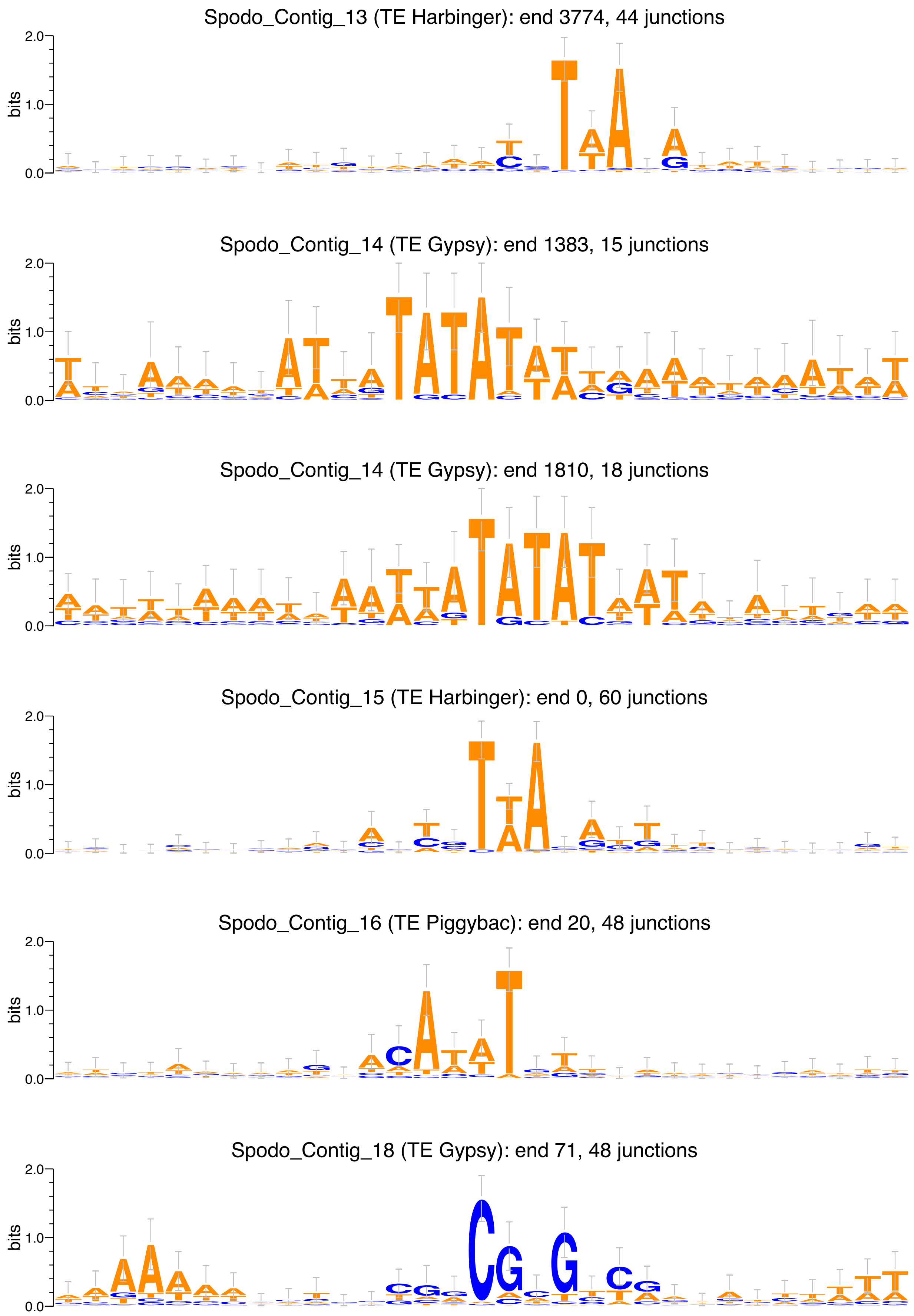

Fig. S4 (continued)

Spodo_Contig_18 (TE Gypsy): end 6616, 32 junctions

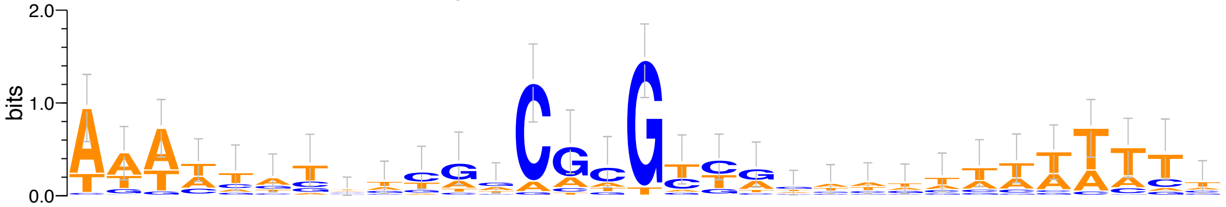

Spodo_Contig_19 (unknown nature): end 0, 13 junctions

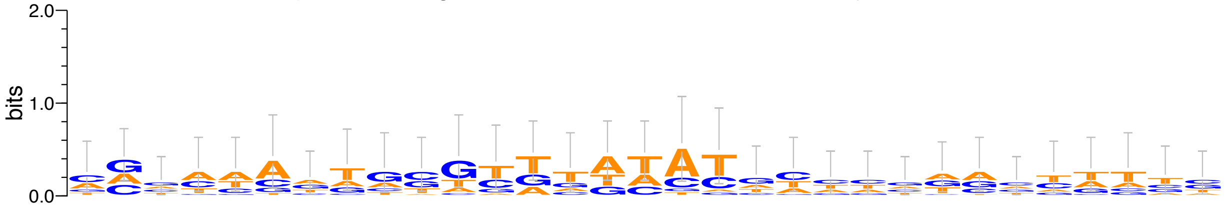

Spodo_Contig_20 (TE Harbinger): end 0, 15 junctions

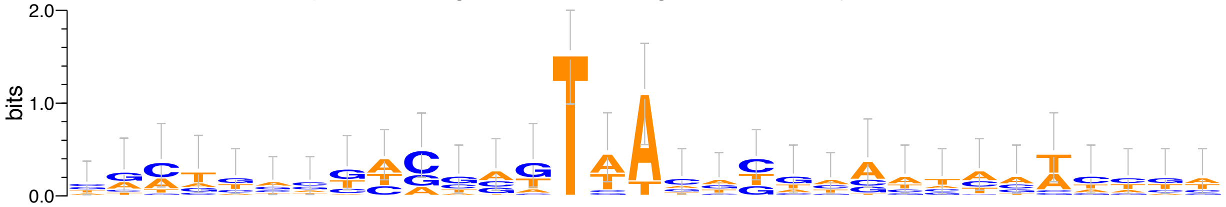

Spodo_Contig_20 (TE Harbinger): end 3074, 33 junctions

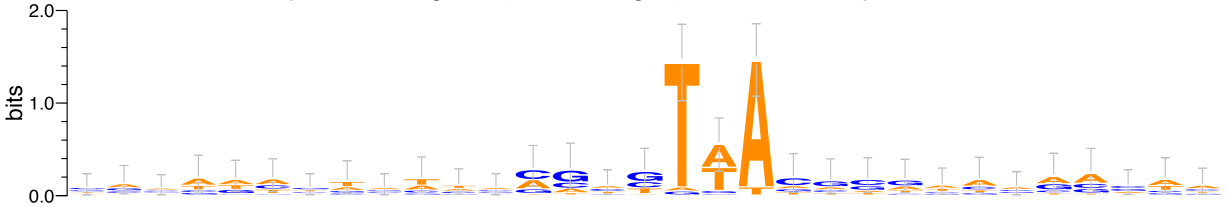

Spodo_Contig_21 (TE Harbinger): end 9, 34 junctions

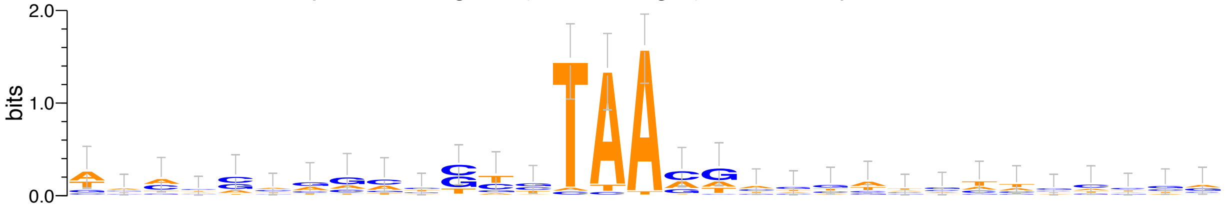

Spodo_Contig_21 (TE Harbinger): end 732, 25 junctions

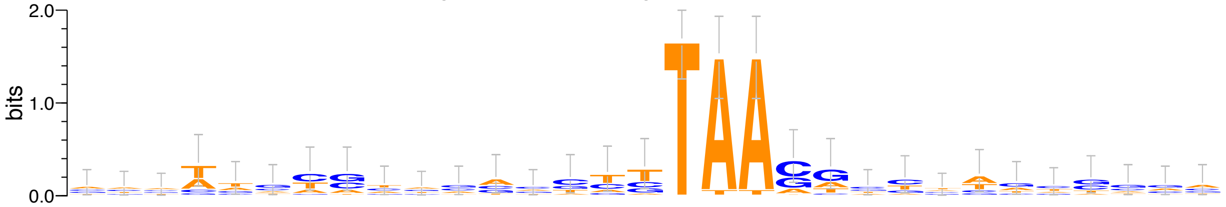

Fig. S4 (continued)

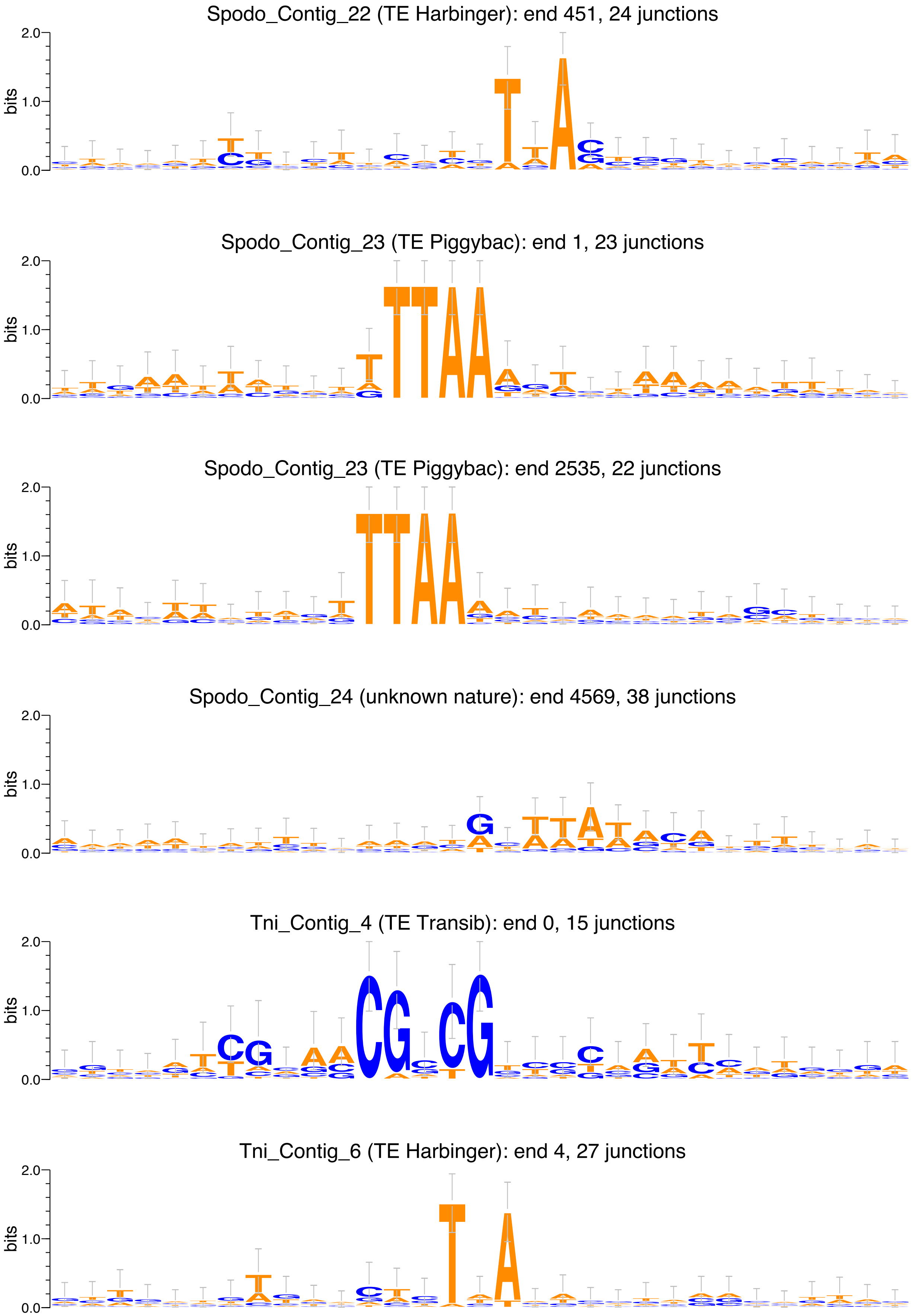

Fig. S4 (continued)

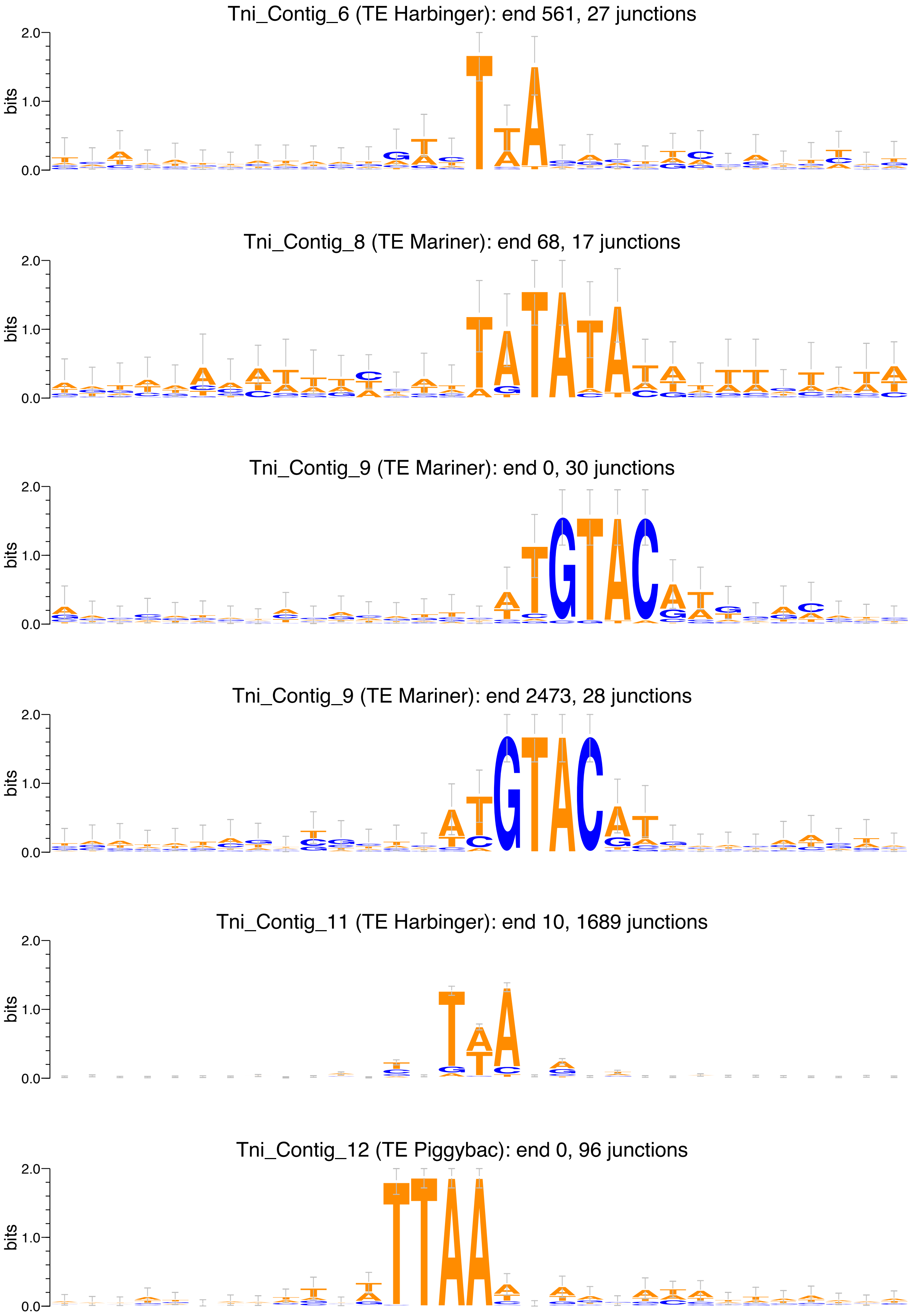

Fig. S4 (continued)

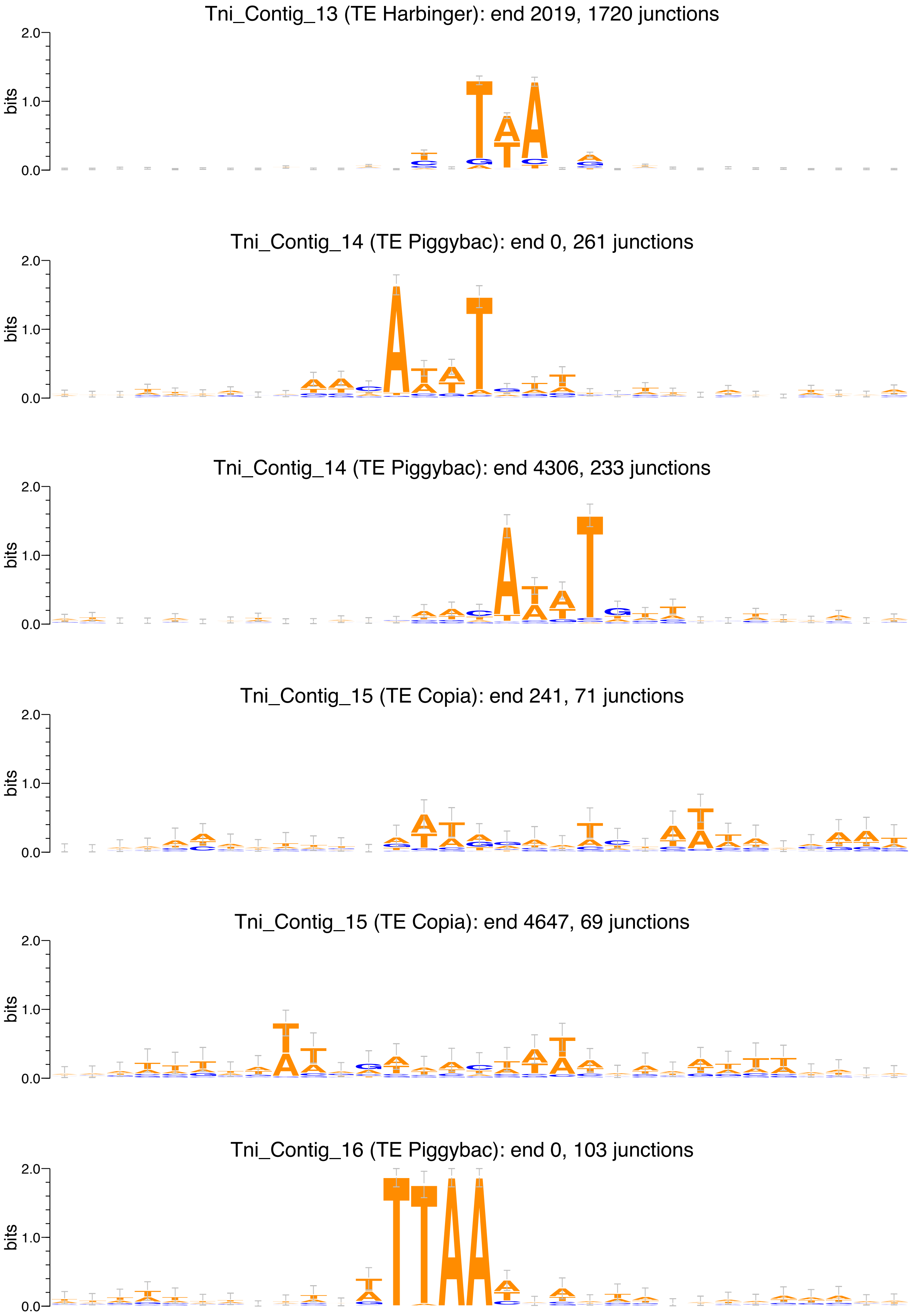

Fig. S4 (continued)

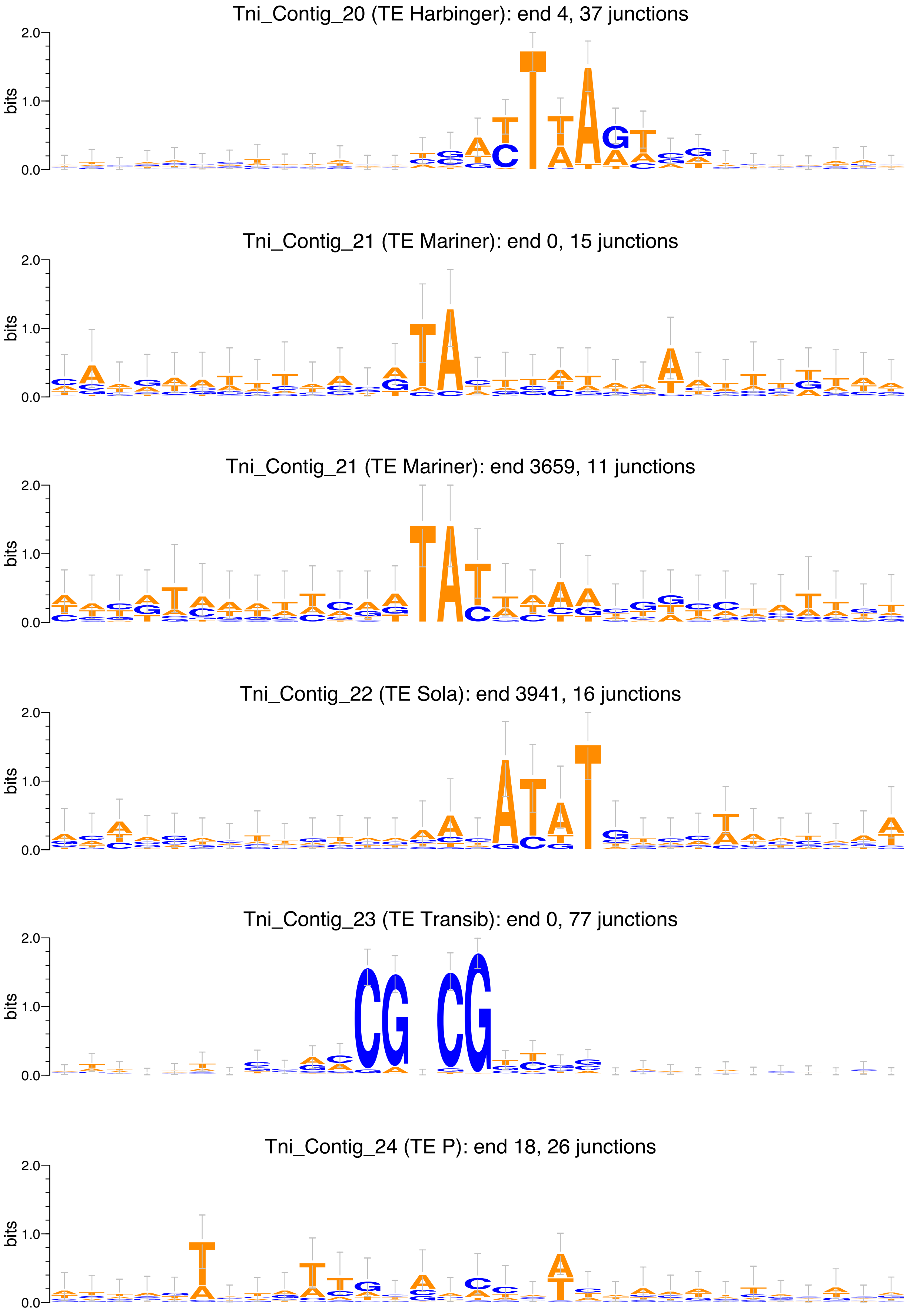

Fig. S4 (continued)

Tni_Contig_24 (TE P): end 4253, 32 junctions

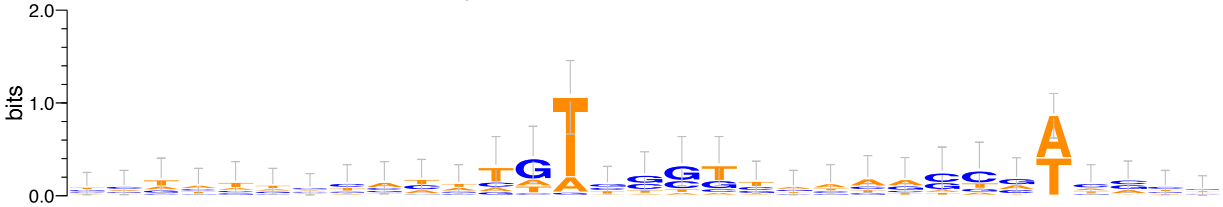

Fig. S4 (continued)
