# Supplementary material for: Continuous Influx of Genetic Material from Host to Virus Populations
Source: PLoS Genet. 2016 Feb 1;12(2):e1005838. doi: 10.1371/journal.pgen.1005838 (PMC4735498; doi:10.1371/journal.pgen.1005838)
Supplement: S5 Fig — Arrows represent genes. Insertions of the same sequence at the same position were counted only once. Insertions of Trichoplusia ni sequences are in blue while those of Spodoptera exigua are in black. The orientation of the insertions is the same as that of the genes represented on the same side of the viral genome (sense: top; antisense: bottom). (PDF) [file pgen.1005838.s010.pdf]

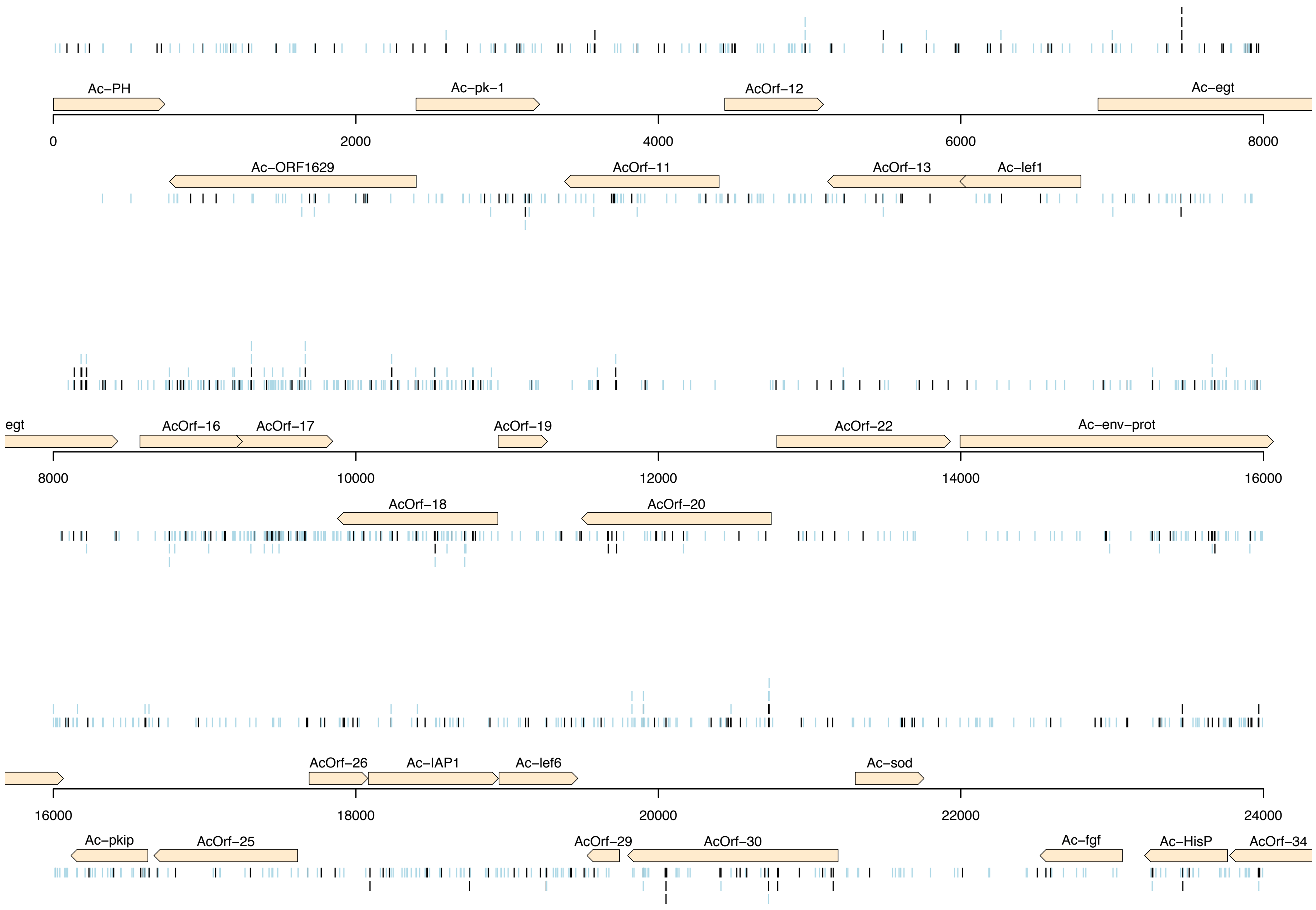

Fig. S5. Detailed map of insertions of moth DNA along the AcMNPV genome. Arrows represent genes. Insertions of the same sequence at the same position were counted only once. Insertions of *Trichoplusia ni* sequences are in blue while those of *Spodoptera exigua* are in black. Insertions shown below the coordinate axis involved the negative strand of the virus genome.

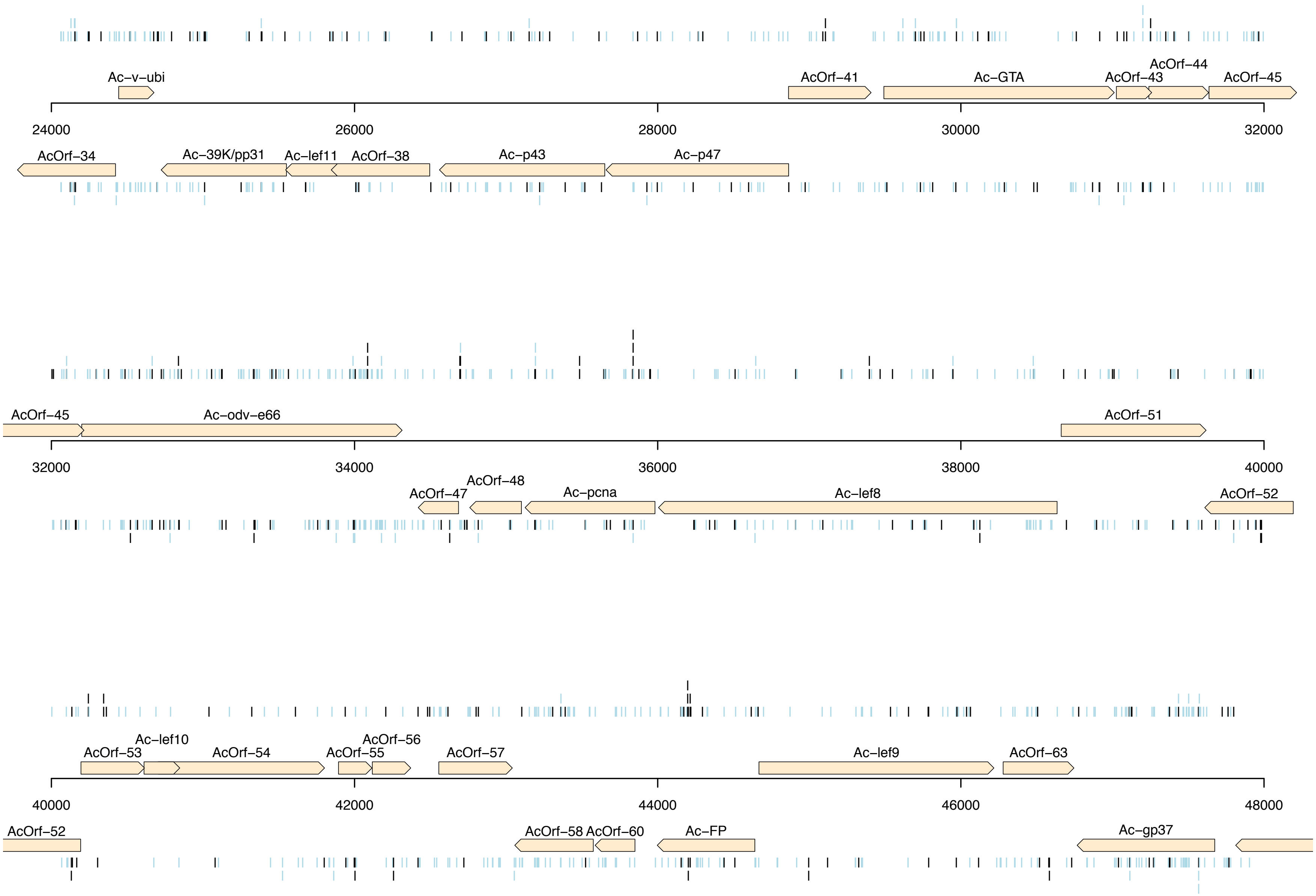

Fig. S5 (continued)

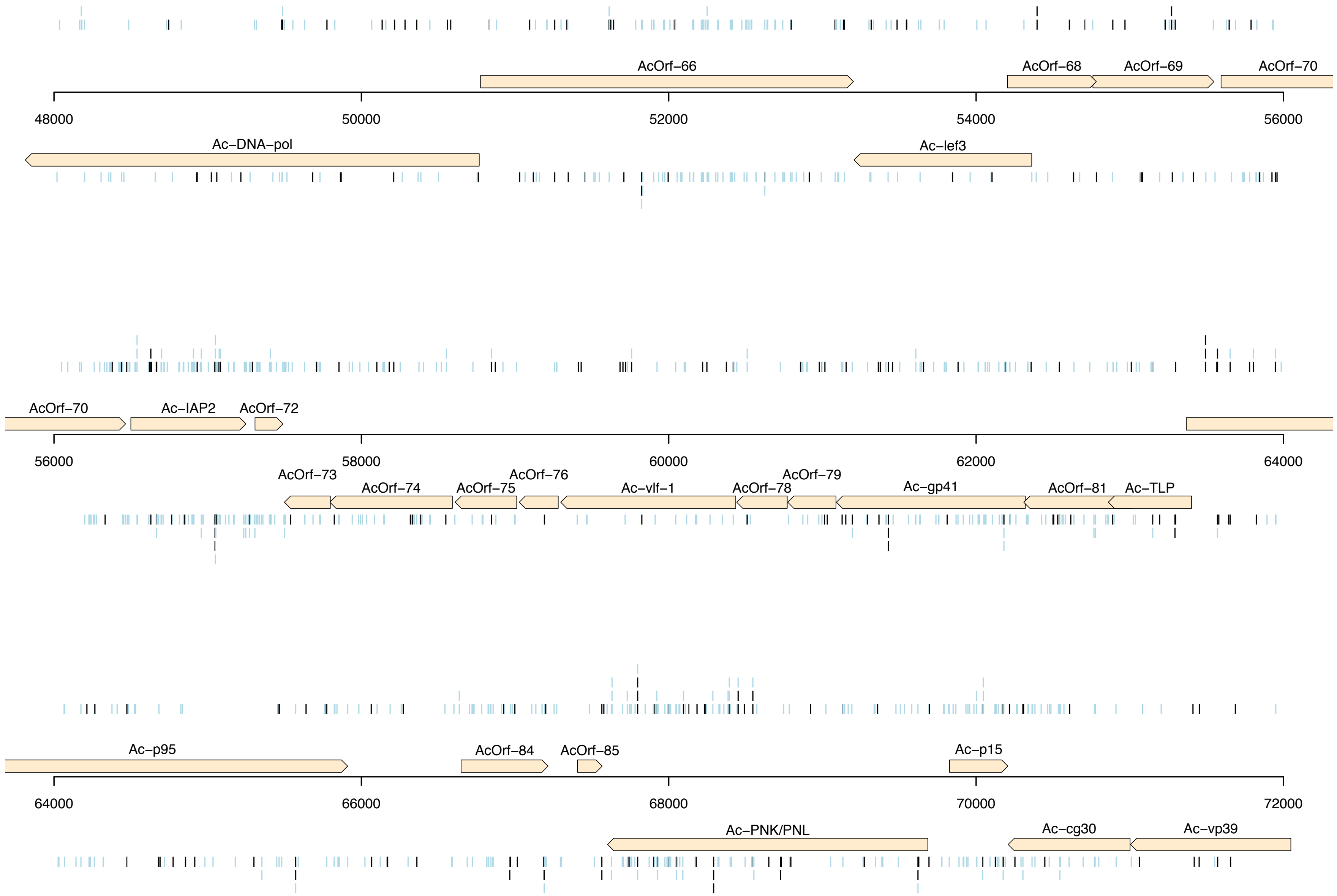

Fig. S5 (continued)

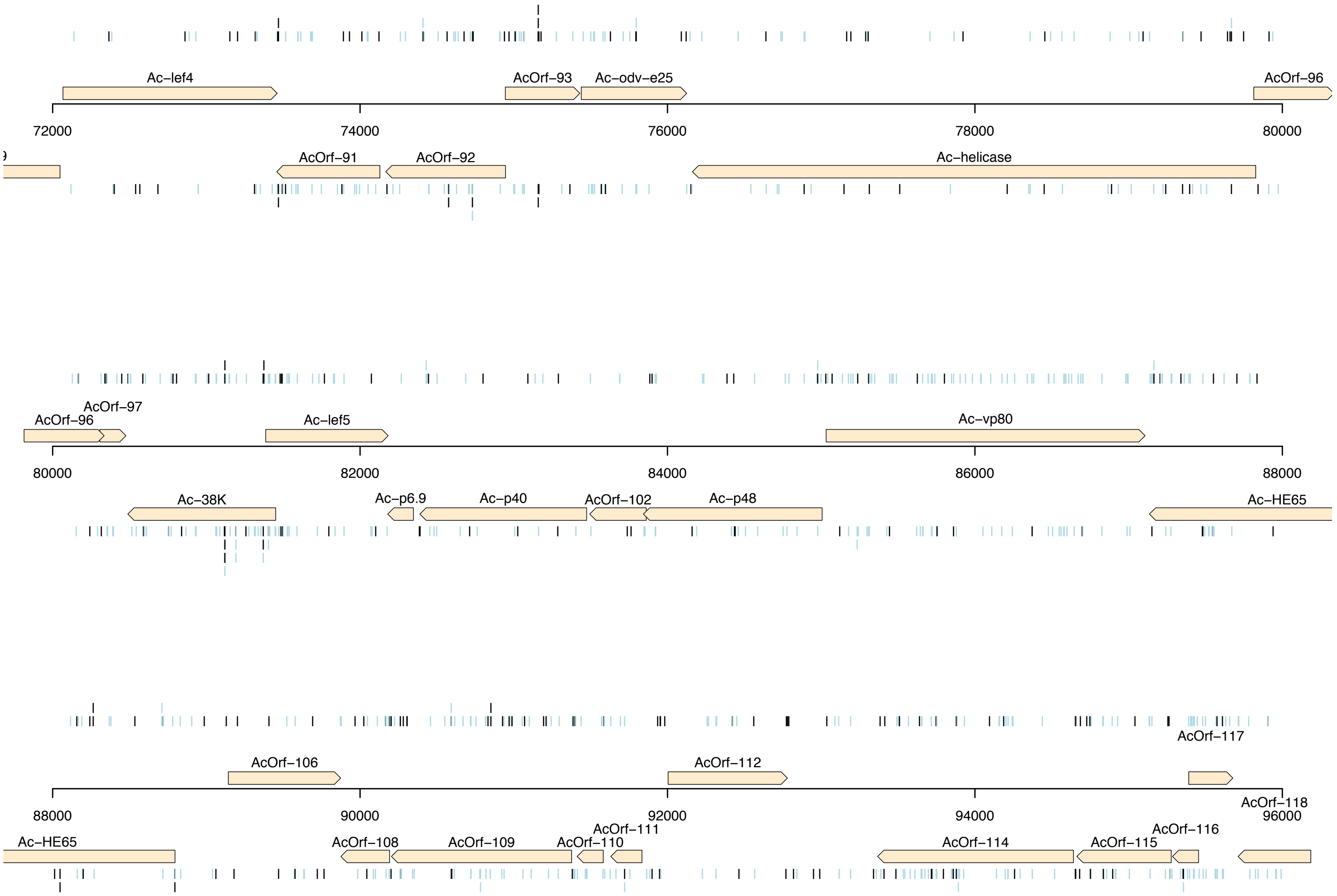

Fig. S5 (continued)

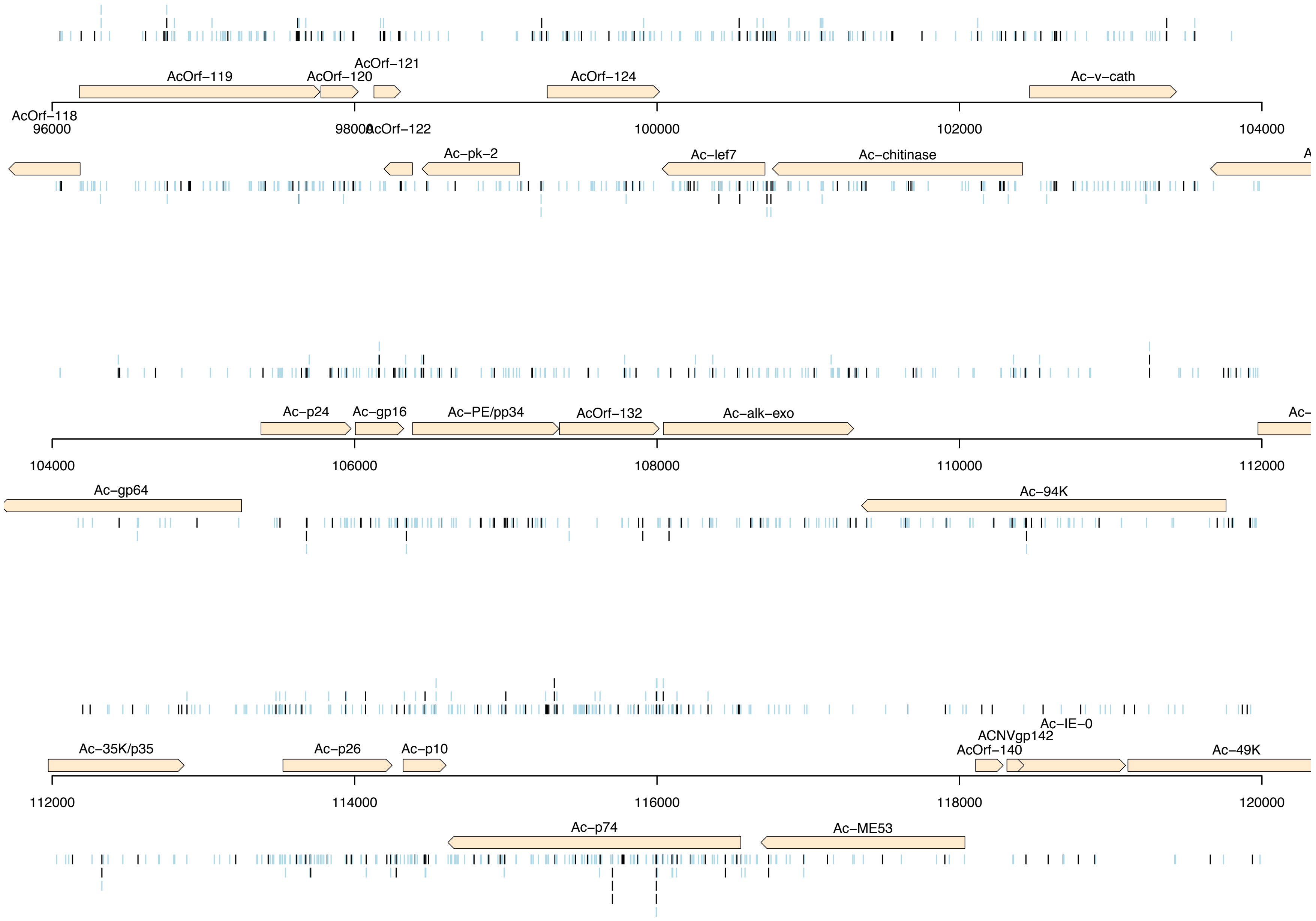

Fig. S5 (continued)

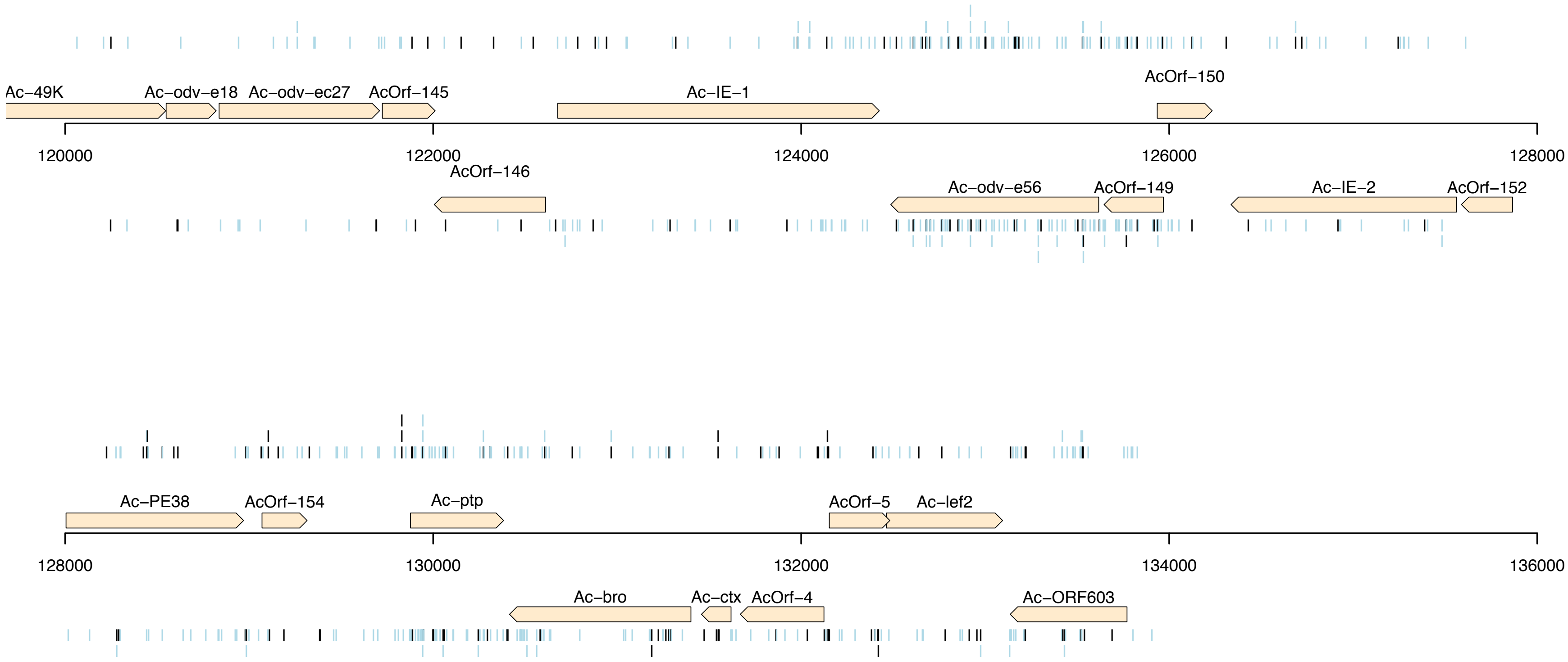

Fig. S5 (continued)
