# Supplementary material for: Continuous Influx of Genetic Material from Host to Virus Populations
Source: PLoS Genet. 2016 Feb 1;12(2):e1005838. doi: 10.1371/journal.pgen.1005838 (PMC4735498; doi:10.1371/journal.pgen.1005838)
Supplement: S6 Fig — This example shows an actual chimeric read having similarities to both the virus genome (right part of read) and a host contig (left part of read). We tolerated only minimal overlap between these similarities so as to discard reads from virus regions that happen to be similar with some host contig (in which case there would be no region aligning with the host contig only) or vice versa. Virus and host regions that have no similarity with the read are shown in grey. Above or below a type of alignment (or overlap) is a bar plot representing the numbers of reads observed from all possible alignment (overlap) lengths. The top left bar plot shows that most of the regions over which chimeric reads align only on host contig are longer than 16 bp. The top right bar plot shows that most of the regions over which chimeric reads align only to the viral genome are longer than 16 bp. The middle bar plot indicates that the regions including the host-virus junction over which the chimeric reads align both to virus and the host contig are mostly between -2 (2-bp deletion) and 20 bp long. The bottom bar plot indicates that most chimeric reads align either to the virus or to a host contig over 101 bp. These plots only include reads having similarities to both a host contig and the virus genome and belonging to genomic libraries generated from virus lines grown in S. exigua, which consist in 101-bp reads. (PDF) [file pgen.1005838.s011.pdf]

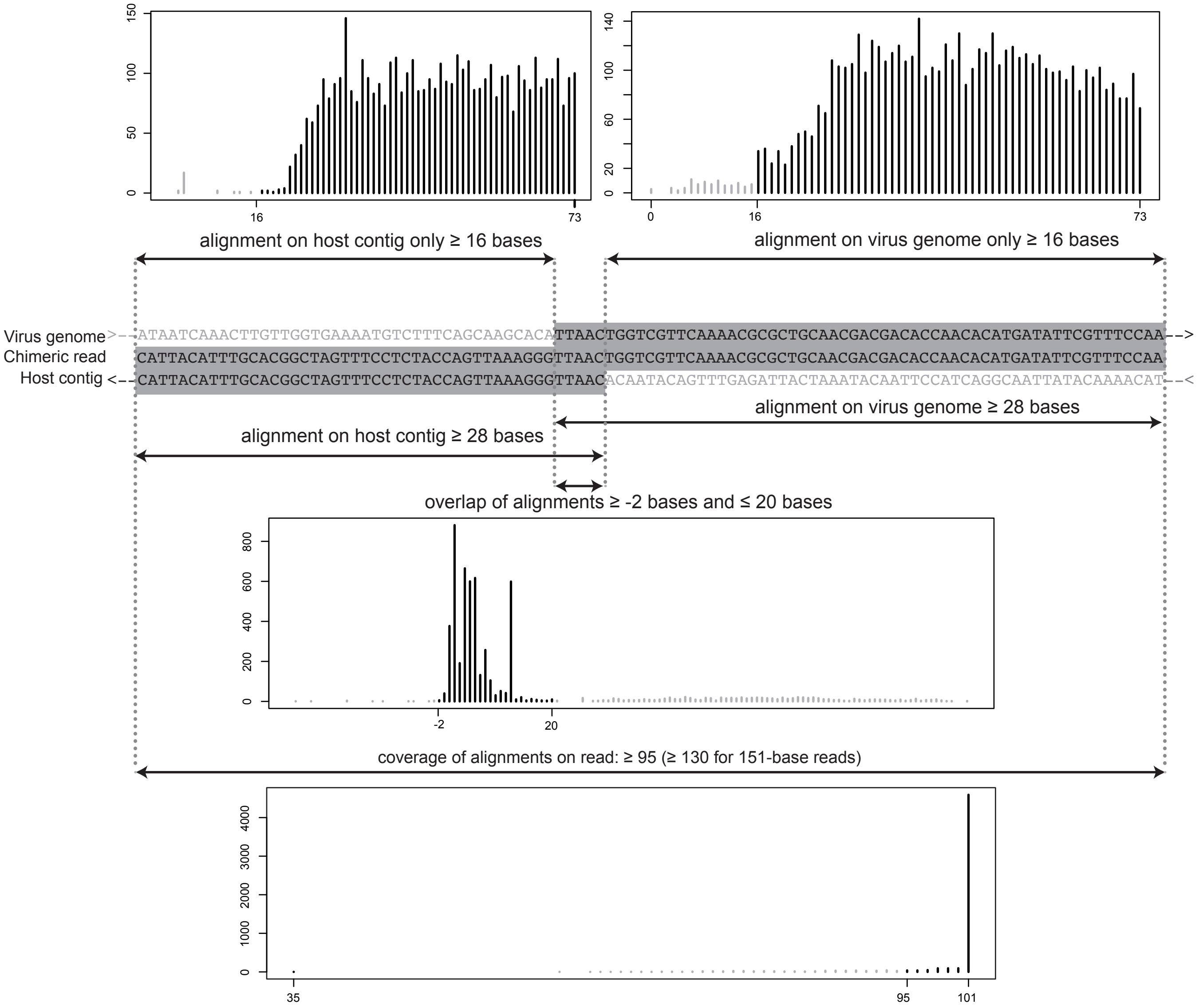

Fig. S6. Criteria used to define chimeric reads that represent junctions between host and virus DNA. This example shows an actual chimeric read having similarities to both the virus genome (right part of read) and a host contig (left part of read). We tolerated only minimal overlap between these similarities so as to discard reads from virus regions that happen to be similar with some host contig (in which case there would be no region aligning with the host contig only) or vice versa. Virus and host regions that have no similarity with the read are shown in grey. Above or below a type of alignment (or overlap) is a bar plot representing the numbers of reads observed from all possible alignment (overlap) lengths. The top left bar plot shows that the region over which the vast majority of chimeric reads align only on host contig is longer than 16 bp. The top right bar plot shows that the region over which the vast majority of chimeric reads align only to the viral genome is longer than 16 bp. The middle bar plot indicates that the region including the host-virus junction over which the chimeric reads align both to virus and the host contig are mostly between -2 (2-bp deletion) and 20 bp. The bottom bar plot indicates that most chimeric reads align either to the virus or to a host contig over 101 bp. These plots only include reads having similarities to both a host contig and the virus genome and belonging to genomic libraries generated from virus lines grown in S. exigua, which consist in 101-bp reads.
