# Supplementary material for: Continuous Influx of Genetic Material from Host to Virus Populations
Source: PLoS Genet. 2016 Feb 1;12(2):e1005838. doi: 10.1371/journal.pgen.1005838 (PMC4735498; doi:10.1371/journal.pgen.1005838)
Supplement: S7 Fig — The black segments represent regions of mismatch between the reads and the contig and grey segments represent similarities. Note the clustering of reads at the contig ends and the fact that, for each end, the same part of the reads (right OR left) aligns with the host contig. The other reads scattered between the two ends do not represent junctions by transposition. (PDF) [file pgen.1005838.s012.pdf]

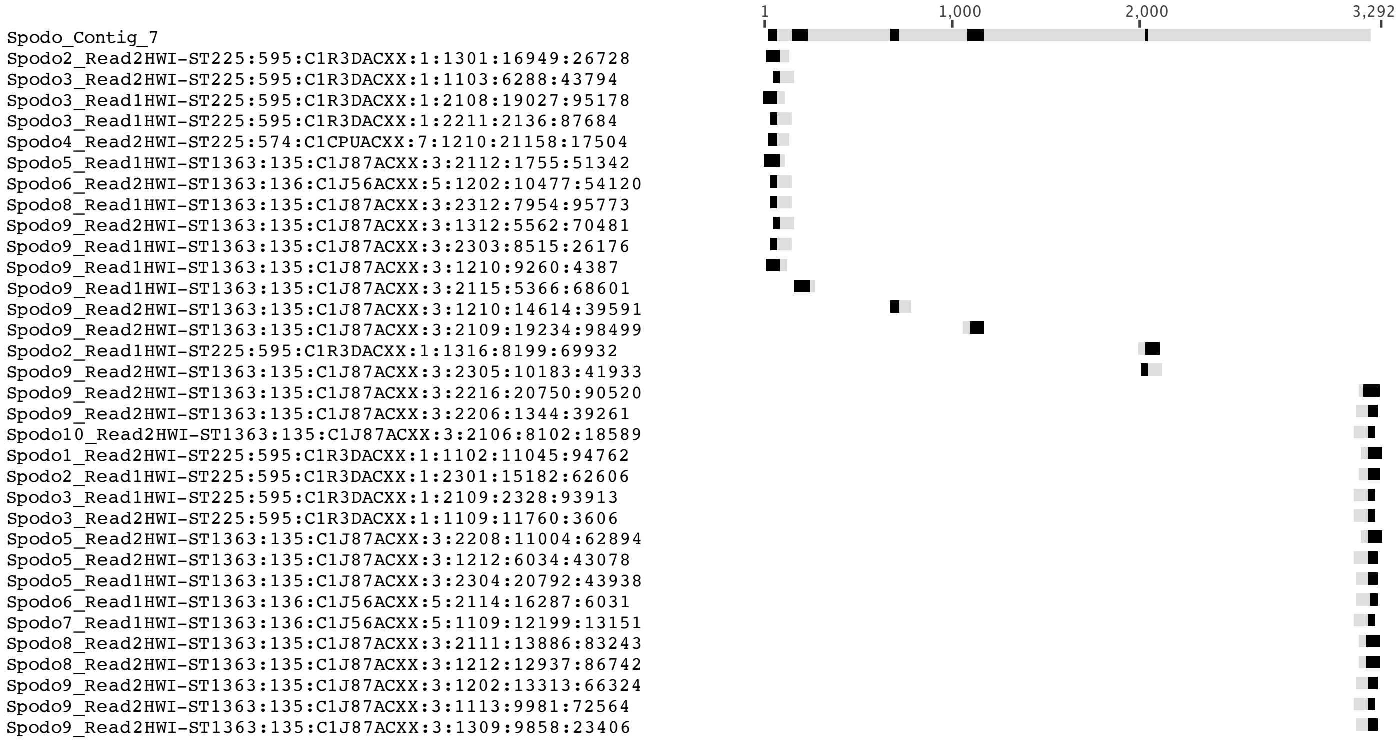

Fig. S7. Visualization of an alignment between a host contig and chimeric reads in Geneious (modified). The black segments represent regions of mismatch between the reads and the contig and grey segments represent homologies. Note the clustering of reads at the contig ends and the fact that for each end the same part of the reads (right or left) aligns with the host contig. The other reads scattered between the two ends do not represent junctions by transposition.
