# Supplementary material for: Continuous Influx of Genetic Material from Host to Virus Populations
Source: PLoS Genet. 2016 Feb 1;12(2):e1005838. doi: 10.1371/journal.pgen.1005838 (PMC4735498; doi:10.1371/journal.pgen.1005838)
Supplement: S8 Fig — These were used to compute an offset that identifies a junction between host and virus DNA (see S1 Text). (PDF) [file pgen.1005838.s013.pdf]

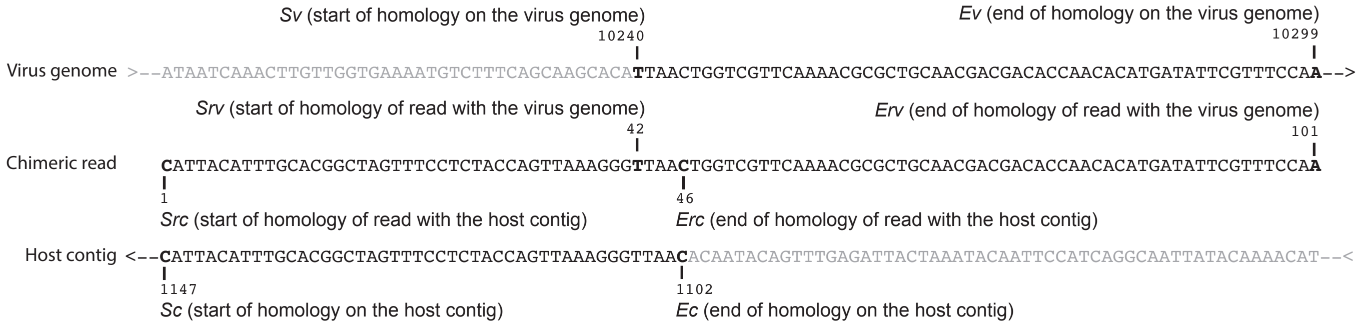

Fig. S8. Coordinates of alignments between a chimeric read and the virus genome and host transcriptome, as returned by blast. These were used to compute an offset that identifies a junction between host and virus DNA (see methods).
