# Supplementary material for: Continuous Influx of Genetic Material from Host to Virus Populations
Source: PLoS Genet. 2016 Feb 1;12(2):e1005838. doi: 10.1371/journal.pgen.1005838 (PMC4735498; doi:10.1371/journal.pgen.1005838)
Supplement: S1 Text — (DOCX) [file pgen.1005838.s017.docx]

Continuous influx of genetic material from host to virus populations

Clément Gilbert, Jean Peccoud, Aurélien Chateigner, Bouziane Moumen, Richard Cordaux, Elisabeth Herniou

# S1 Text

## Investigation of technical duplicates

Illumina-based sequencing involves PCR amplification of the source DNA during library preparation. We investigated whether several chimeric reads could result from PCR amplification of a single original junction. We delineated groups of chimeric reads having identical coordinates of alignments to the virus genome and a host sequence, and resulting from the same genomic library. To test whether these reads were sequenced from the same PCR amplicon, we compared their mates, which in this case should be identical (barring sequencing errors). Identity was estimated by comparing all mates of a read group base by base at the same positions. We did so instead of comparing alignment coordinates of mates because some mates may not be present in blast outputs (for example, if they were sequenced from host DNA fragments not present in the host transcriptome or assembled contigs). Based on this identity, we determined that 141 chimeric reads were duplicates of others.

Another source of technical duplication is the possibility for a junction of host and virus DNA to be sequenced twice in both directions and to appear in each mate of a read pair. This was the case for 543 read pairs that covered the same junctions (junctions were characterized as described below).

We removed duplicated junctions from our counts shown in S1 Table by only retaining the read with best alignment score on a host contig among duplicates or overlapping reads.

## Identification of junctions between host and virus DNA

Among junctions between the AcMNPV genome and a moth contig, some are viral replicates of the same original junction (i.e. host-virus junctions resulting from insertion of a host sequence in the viral genome followed by amplification in the viral population through viral replication) and must be characterized as such. This characterization is not possible for junctions that occurred between paired reads, as the position of the junction points cannot be precisely located in respect to the virus genome and host contig. These types of junctions were hence discarded for all analyses based on junction locations.

A junction can be identified by the host DNA sequence it involves and its inferred location in the target viral genome. The latter was considered suboptimal because (i) it may vary between viral replicates of an original junction due to mutations and sequencing errors and (ii) it may not differentiate the two junctions involving both ends of the same inserted DNA fragment and insertions into opposite orientations. In order to take these confounding factors into account, we computed an offset between the host sequence coordinates and the virus genome coordinates (S8 Fig), which is resilient to point mutations and sequencing errors, for every chimeric read as follows:

$O=\left\{ \begin{aligned} Sv+K_{1}\left( Srv-Erc \right)-K_{2}\times Ec, &homology with the host contig comes first in the read \\ Ev+K_{1}\left( Erv-Src \right)-K_{2}\times Sc, &\mathrm{otherwise} \end{aligned} \right.$ (Equation 1)

with

$$K_{1}=\left\{ \begin{aligned} 1, & virus genome and read align in opposite directions \\ -1, & \mathrm{otherwise} \end{aligned} \right.$$

$$K_{2}=\left\{ \begin{aligned} 1, & virus and host sequences align with the read in both plus or both minus\mathrm{direction} \\ -1, & otherwise. \end{aligned} \right.$$

*Sv*, *Srv*, *Erc*, *Ec*, *Ev*, *Src* and *Sc* are positions of starts and ends of alignments returned by blastn, as illustrated in S8 Fig. Note that *K*_2_ = 1 if the insertion of host DNA occurred in the positive strand of the virus genome. The name of the host contig involved, *O* and *K*_2_ were used together to identify each junction.

## Insertions of *T. ni* sequences in virus extracted from *S. exigua*

To assess whether virus carrying insertions of host DNA can be transmitted over several rounds of infection, we searched for insertions of *T. ni* sequences in viruses extracted from *S. exigua* (which descend from the G0 population of virus produced in *T. ni*, see S1 Fig).

Our filters applied to results of blastn searches of virus reads from *S. exigua* lines against the *T. ni* contigs retained 1360 chimeric reads and 472 chimeric read pairs. Among those, 27 chimeric reads and 8 chimeric read pairs were not found by blast searches against the *S. exigua* contigs, suggesting that they comprise DNA sequences from *T. ni*, the moth species on which the initial viral population was amplified (S1 Fig). Those chimeric reads and read pairs represent at most 24 independent junctions. For chimeric reads pairs, we are not able to locate the precise insertion points, so the minimum number of different junctions here represents the number of different *T. ni* contigs (here two) that have homologies with chimeric read pairs.

None of the 22 junctions that could be located in the virus genome was detected in viruses from the G0 population. This may be due to the fact that those junctions were not sequenced in the G0 and/or to the fact that they are not actually composed of *T. ni* sequences, but instead of *S. exigua* sequences not present in the *S. exigua* contigs and that happen to be homologous to *T. ni* sequences. Under the latter hypothesis, those junctions would not have been inherited from the G0 population.

## Characterization of integration mechanisms and conserved sequences at transposition sites

A transposable element that inserted many times in a target genome is expected to align with many chimeric reads at positions that correspond to the ends of the TE. Clustering of alignments of chimeric reads onto a contig (as in S7 Fig) was thus used as an indication that transposition was involved.

To automatically identify these clusters, we defined, for each chimeric read involving a given contig, the position of the junction in the contig as the coordinate at which it stops aligning with the read (*Ec* or *Sc* in S8 Fig, depending on whether the region of the chimeric read aligning to the host contig is located upstream or downstream of the region aligning to the virus genome). This position can vary between insertions of identical host DNA fragments, due to homology between the fragment end and the insertion site (leading to the overlap shown in S6 Fig and S8 Fig), mutations and sequencing errors. It was thus allowed to vary within a group of reads. Junctions that clustered together and involved the same host sequence all differed from their closest one by less than 6 bp and their chimeric reads aligned with the host contig by the same end (i.e., all chimeric reads align on the contig at the left OR right of the host fragment end). We thus used these two criteria to delineate clusters of junctions. A cluster also had to be formed by three reads or more.

We built sequence conservation logos for each cluster of at least ten junctions involving the same end of a host sequence. To do this, we fixed the position of the sequence end as the most common position among junctions (in contig coordinates). Whether this position exactly corresponds to the end of the inserted fragment is not crucial. Based on this defined end position we call *E*, we derived the corresponding insertion site in virus genomes for each junction as *K*_2_ × *E* + *O*, *O* being the offset computed with equation 1.

Among the remaining host-virus junctions, some did not form clusters according to our criteria (defined above) and were scattered along host contigs, suggesting that different fragments of the contigs were inserted. If these junctions were associated with a contig involved in six junctions or more, they were judged highly unlikely to result from transposition (otherwise they would be included in clusters). This concerned 434 junctions. Thirty-six junctions that did not form clusters and were in contigs comprising less than six junctions were not characterized in terms of insertion mechanism.

**Investigation of contamination of viral samples by host DNA**

Several lines of evidence suggest that, if present, the level of contamination of our viral samples by host DNA must be very low. First, in addition to the DNAse treatment we performed before dissolving AcMNPV occlusion bodies (Gilbert et al. 2014), we checked for the presence of contaminating host DNA using PCR on a nuclear (actin) and mitochondrial (COI) marker. These PCR were negative for all viral DNA samples. Second, if the viral DNA samples were contaminated by host DNA, one would expect to find viral reads corresponding to a large fraction of the host genome. Yet, the output of our first blastn step carried out to identify host-virus junctions (viral reads against moth transcriptomes and contigs) revealed that only 0.8% and 0.3% of the bases available in the 60-Mb *T.ni* transcriptome and 108-Mb *S. exigua* transcriptome were covered by at least one read, respectively. In addition, much like in our previous study reporting Piggybac and Mariner TE copies integrated in the AcMNPV genomes recovered from *T. ni* infections (Gilbert et al. 2014), we were able to recover by PCR several (n = 7) of the *S. exigua*-AcMNPV junctions (Dataset S1), further suggesting that the host-virus junctions detected computationally are unlikely to be technical chimeras.

Gilbert C, Chateigner A, Ernenwein L, Barbe V, Bézier A, Herniou EA, Cordaux R. 2014. Population genomics supports baculoviruses as vectors of horizontal transfer of insect transposons. *Nat Commun* **5**: 1-9.
